# Detection of candidate genes affecting milk production traits in sheep using whole‐genome sequencing analysis

**DOI:** 10.1002/vms3.731

**Published:** 2022-01-11

**Authors:** Elham Rezvannejad, Hojjat Asadollahpour Nanaei, Ali Esmailizadeh

**Affiliations:** ^1^ Department of Biotechnology Institute of Science and High Technology and Environmental Sciences Graduate University of Advanced Technology Kerman Iran; ^2^ Faculty of Agriculture Department of Animal Science Shahid Bahonar University of Kerman Kerman Iran

**Keywords:** candidate genes, milk production, sheep, whole‐genome sequencing analysis

## Abstract

**Background:**

Artificial and natural selection for important economic traits and genetic adaptation of the populations to specific environments have led to the changes on the sheep genome. Recent advances in genome sequencing methods have made it possible to use comparative genomics tools to identify genes under selection for traits of economic interest in domestic animals.

**Objectives:**

In this study, we compared the genomes of Assaf and Awassi sheep breeds with those of the Cambridge, Romanov and British du cher sheep breeds to explore positive selection signatures for milk traits using nucleotide diversity (Pi) and FST statistical methods.

**Methods:**

Genome sequences from fourteen sheep with a mean sequence depth of 9.32X per sample were analysed, and a total of 23 million single nucleotide polymorphisms (SNPs) were called and applied for this study. Genomic clustering of breeds was identified using ADMIXTURE software. The FST and Pi values for each SNP were computed between population A (Assaf and Awassi) and population B (Cambridge, British du cher, and Romanov).

**Results:**

The results of the PCA grouped two classes for these five dairy sheep breeds. The selection signatures analysis displayed 735 and 515 genes from FST and nucleotide diversity (Pi) statistical methods, respectively. Among all these, 12 genes were shared between the two approaches. The most conspicuous genes were related to milk traits, including *ST3GAL1* (the synthesis of oligosacáridos), *CSN1S1* (milk protein), *CSN2* (milk protein), *OSBPL8* (fatty acid traits), *SLC35A3* (milk fat and protein percentage), *VPS13B* (total milk production, fat yield, and protein yield), *DPY19L1* (peak yield), *CCDC152* (lactation persistency and somatic cell count), *NT5DC1* (lactation persistency), *P4HTM* (test day protein), *CYTH4* (FAT Production) and *METRNL* (somatic cell), *U1* (milk traits), *U6* (milk traits) and *5S_RRNA* (milk traits).

**Conclusions:**

The findings provide new insight into the genetic basis of sheep milk properties and can play a role in designing sheep breeding programs incorporating genomic information.

## Graphical abstract

The purpose of the present study was to identify important genomic regions and genes associated with milk production traits in dairy sheep breeds. Using whole genome sequences analysis important candidate genes related to sheep milk production were identified.

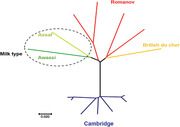

## 1 INTRODUCTION

The sheep was one of the first herbivores to be partially domesticated due to its controllable size and ability to adapt to different climates and diets with poor nutrition. After artificial selection, different breeds were formed with different morphology, coat colour or main production (milk or wool and meat). For the last few decades, reproductive breeding methods using calculated breeding values of reproductive traits from pedigree and phenotype information have significantly increased sheep performance (Goddard & Hayes 2009; Niemann & Kues 2003).

In recent years, advances in high‐throughput genome scanning methods, chiefly whole‐genome sequencing, SNP genotyping array, and comparative genomic hybridisation (CGH) arrays, have allowed identifying causal genes and variants related to different traits. Compared to the other two methods, the next‐generation sequencing method has been proven to be a valuable strategy for detecting functional genes and genetic variants related to important economic traits in sheep (Jiang et al., 2014).

Sheep milk is a notable source of income, computing for a large part of universal milk production, and is generally applied to yield many cultured dairy supplies, like cheese. Milk traits can be defined by calculating yield, fat, lactose percentage and protein. Because local multi‐purpose breeds mainly produce sheep's milk with low to moderate milk outputs, few sheep breeds have been expanded obviously to produce milk. To date, only a few studies including GWAS and selective sweep experiments have been performed to identify genomic regions affecting milk traits in sheep using the SNP array (Eydivandi et al., 2021; Garcia‐Gamez et al., 2012; Gutiérrez‐Gil et al., 2014; Moioli et al., 2013; Rupp et al., 2015; Yuan et al., 2019) and RNA‐Sequencing methods (Giordani et al., 2017; Suarez‐vega et al., 2015; Suarez‐vega et al., 2016).

A positive selection signature analysis has previously been used to detect the genes/gene regions influenced by selection using genome comparison of different phenotypic breeds. For this reason, the sequencing of the reference genome assemblies of the sheep (Ovisaries) simplified efforts to realise the genetic basis of phenotypic differences of several sheep breeds with re‐sequencing methods.

Various methods have been expanded and applied to distinguish the positive selection signatures in the genomes of different livestock species or breeds. Some are based on significantly reduced nucleotide diversity levels, long‐range haplotype homozygosity (Sabeti et al., 2007) and high population differentiation (Bahbahani et al., 2015; Porto‐Neto et al., 2014). Information on the genetic diversity of these genes in different sheep populations can be interesting. For example, evidence of selection signatures around these genes could indicate a potential direct effect of the desired gene on phenotypes of interest for the dairy sheep industry. In this research, nucleotide diversity (Pi) and FST statistical methods were applied to compare the genomes of Assaf and Awassi breeds with Cambridge, Romanov and British du Cher breeds in order to explore positive selection signatures for milk traits and to find new candidate genes in the genomic regions under selection.

## 2 MATERIALS AND METHODS

### 2.1 Sequencing data and mapping

We utilised 14 whole genomes, including an Assaf breed (accession number, ERR1163920, an Awassi breed (accession number, ERR1163918), seven Cambridge breeds (accession number, ERR1163918), four Romanov breeds (accession numbers, ERR1456968, ERR1456970, ERR1456972 and ERR1456974) and a British du Cher breed (accession number, ERR1456963). The data were downloaded from the Sequence Read Archive at the NCBI site (https://trace.ncbi.nlm.nih.gov/Traces/sra). Burrows‐Wheeler Aligner (BWA) (Li & Durbin 2009) was applied to aligned reads against the reference genome assembly V3.0 (ftp://ftp.ensembl.org/pub/release‐91/fasta/ovis_aries/dna/). By using SAMtools (Li et al., 2009), All Sequence Alignment MAP (.sam) files were changed to the Binary Alignment MAP (.bam) files, followed by sorting and indexing them. PCR duplicates were removed from the .bam files using the Picard program (https://github.com/broadinstitute/picard).

### 2.2 Studied population

The Assaf breed is the result of crossbreeding the Awassi and East Friesian from Israel and is raised for milk and meat. The Awassi originated from the Syro‐Arabian desert in South‐West Asia and primarily is raised for milk. The Cambridge breed originated in 1964 at Cambridge University, a crossbred sheep from mating between Landrace rams with many of local ewes and has high prolificacy. Romanov developed from Russia. This breed is raised primarily for meat and has solid and resourceful wool. British du Cher originated from crossbreeding three breeds of Berichon du Cher, Merino and Dishley Leicester and has high standard wool. More details about these breeds are available on http://www.sheep101.info/sheepbreedsa‐z.html and http://www.fao.org/dad‐is/en/.

### 2.3 SNP detection

To increase and improve the quality score of each base, base quality scores were re‐evaluated using the GATK3.4‐46‐gbc02625 program (McKenna et al., 2010). SNPs of all individuals were identified and filtered using GATK Unified Genotyper and GATK Variant Filtration, respectively.

### 2.4 Analyses of the regions of homozygosity

Runs of homozygosity (ROH) segments are introduced as contiguous homozygous regions of the genome, which can provide an insight into a population's history, genomic inbreeding, and signatures of selection (Ceballos et al., 2018; Purfield et al., 2012). The extent and length of these ROHs are different among individuals and populations (Curik et al., 2014; Purfield et al., 2012). We obtained ROH segments for each breed using the ‘runs of homozygosity’ function (–homozyg‐kb) for three length categories (more than 100, 200 and 300 kb) in the program PLINK v1.07 (Purcell et al., 2007). It should be noted that we used default parameters for other adjustments.

### 2.5 Genetic structure analysis

Genomic clustering of breeds was identified using ADMIXTURE software (Alexander & Lange, 2011) with a maximum likelihood approach. The admixture runs were done for *K* values of 1–4. Barplots were produced for *K* values in the dataset. In addition, the different kinds of files (bim, bed and fam files) were created in PLINK software to draw possible population/genetic structure between and within different populations under study. Then these files were handled in an R‐programming environment (http://www.R‐project.org) for eigenvector formation and PCA plotting along two axes. The first two principal components (PCs) (i.e., PC 1 and PC 2) were concurrently calculated for each dataset. In the end, these PCs were graphically plotted against each other in two axes. Also, Nei's genetic distances (Nei, 1972) among different populations were applied to build a neighbour‐joining (NJ) tree ingdsfmt and NPRelate (Zheng et al., 2012; Zheng et al., 2017) packages of R.

### 2.6 Genome‐wide selective sweep scans

To detect genomic regions harbouring footprints of positive selection in five sheep breeds, we used two tests to study selection signatures. Here, we applied the FST and Pi statistical methods. FST values for each SNP were computed between population A (Assaf and Awassi) and population B (Cambridge, British du cher and Romanov). To characterise genetic differentiation (FST) and also nucleotide diversities ((Δπ or ΔPi) = πpopA – πpopB) between two populations, VCFtools v0.1.14 software (https://github.com/vcftools/vcftools) was applied. The values were estimated in a sliding window size of 50 kb and a step size of 25 kb. Pairwise FST values for each SNP were calculated based on Weir and Cockerham method (1984). In both analyses, the top 1% was considered as selection footprints.

### 2.7 Selective sweep region annotation and gene functional enrichment analysis

Candidate selective sweeps discovered with the approaches mentioned above were annotated using the Variant Effect Predictor existing at http://asia.ensembl.org/info/docs/tools/index.html.

Functional enrichment analysis was performed using the ‘g:Profiler’ enrichment analysis tool to identify their biological functions (Reimand et al., 2011). And the *p* value of the gene enrichment was adjusted by Benjamini–Hochberg FDR (false‐discovery rate).

## 3 RESULTS

### 3.1 Output mapping of five sheep breeds

Our efforts produced a mean sequence depth of 9.32× per sample, within a range of 3.82‐ to 12.7‐fold (Table 1). A total of 23 million SNPs were detected in all individuals.

**TABLE 1:** Output mapping for fourteen samples

Sample	Number of sites	Depth
Awassi	23,641,651	12.7
Assaf	23,664,074	11.51
Romany1	23,363,383	4.09
Romany2	22,719,118	3.82
Romany3	22,439,114	4.67
Romany4	23,031,003	4.62
British Du cher	22,998,264	10.16
Cambridge1	23,536,793	12.22
Cambridge2	23,684,977	12.10
Cambridge3	23,628,113	9.34
Cambridge4	23,538,622	10.3
Cambridge5	23,657,712	10.3
Cambridge6	23,680,612	11.89
Cambridge7	23,676,266	11.30
Mean		9.32

### 3.2 Runs of homozygosity

For all breeds, shorter ROHs (100 kb) were more frequent than longer ROHs (200 and 300 kb) (Figure 5). The mean number of ROH segments was highest for the Awassi, while the Romanove breed revealed the lowest average ROH coverage in three length categories. Awassi and Assaf populations had larger long ROHs (200 and 300 kb) than the other breeds.

### 3.3 Genetic structure analysis

**FIGURE 1:**
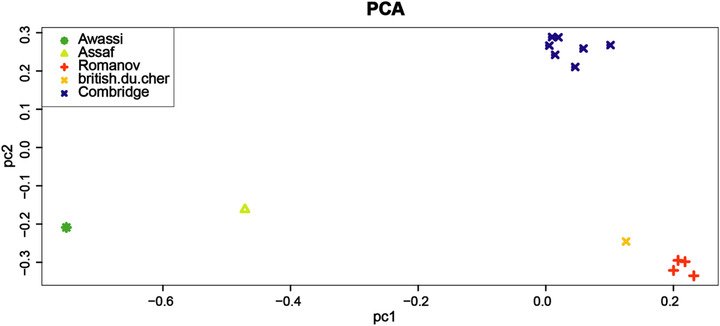
Principal comment analysis of SNPs data set from five sheep breeds

**FIGURE 2:**
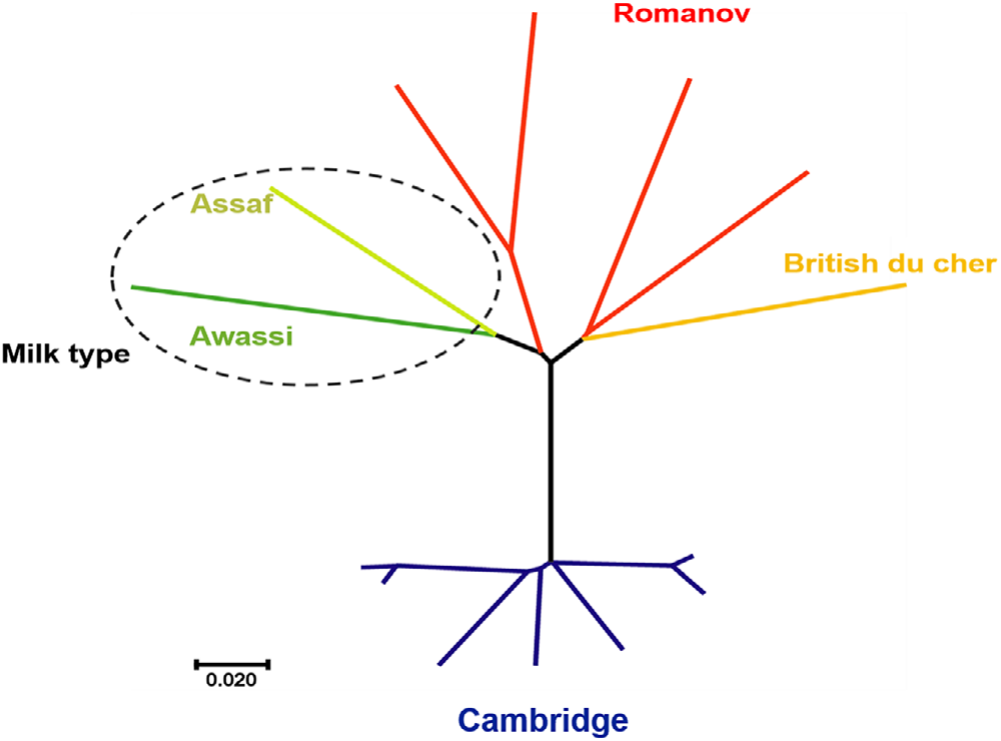
Neighbour joining (NJ) tree based on Nei's genetic distance in which five sheep breeds was separated by clusters

**FIGURE 3:**
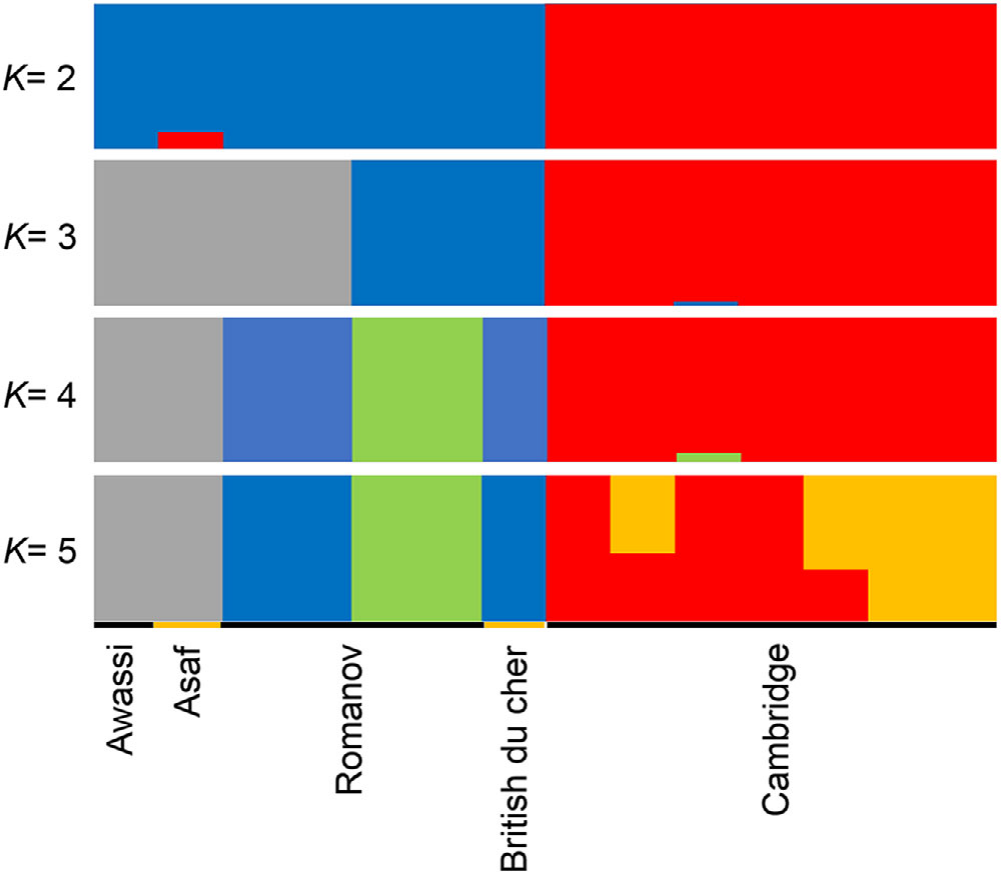
Admixture output for four value of *k* exanimated

Principal components analysis (PCA) was performed to study the genetic separation of five sheep breeds (Figure 1). PCA grouped the five introduced breeds (Cambridge, British du cher, Romanov, Assaf and Awassi). Neighbour‐joining (NJ) tree of Nei's genetic distance of these five sheep breeds acknowledged the results of the PCA; for example, two dairy sheep breeds (Assaf and Awassi) were classified together, and British du cher was genetically close to Romanov (Figure 2). Genomic breed clustering for four values of *K* in five sheep breeds is presented in Figure 3.

### 3.4 Positive selection signature

**FIGURE 4:**
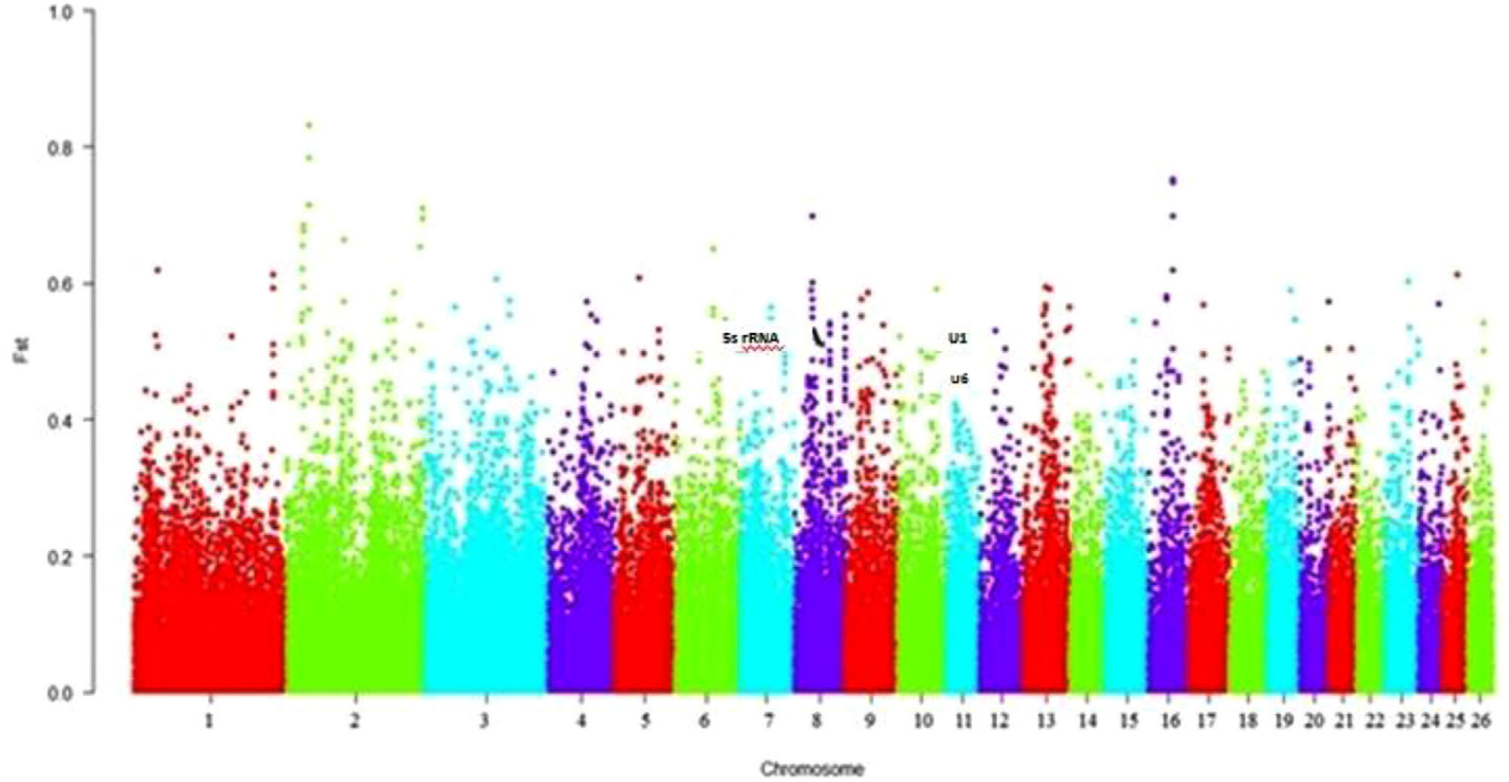
Distribution of FST values across the genome of five sheep breeds

Using nucleotide diversity (Pi) and FST statistical methods, we compared the genomes of Assaf and Awassi breeds with Cambridge, Romanov and British du cher breeds to explore positive selection signatures. The first percentile rank was applied as a threshold to detect candidates all over the analysis. Several protein‐coding genes with significant higher FST (735 genes, Table S1) and a lower value for nucleotide diversity (Pi, 515 genes, Table S2) were detected and examined as potential candidate genes that endured selection during domestication. Among all these, 12 genes were shared between two of the methods (Figure S1). Gene ontology (GO) analysis for the genes showing signatures of positive selection is reported in the table (Table S3). All the terms related to sensory perception are over‐represented (*p* < 0.01). Potential candidate genes for milk traits are listed in the table (Table S4). The most conspicuous genes related to milk traits include *ST3GAL1*, *CSN1S1*, *CSN2*, *OSBPL8*, *SLC35A3*, *VPS13B*, *DPY19L1*, *CCDC152*, *NT5DC1*, *P4HTM*, *CYTH4*, *METRNL*, *U1*, *U6* and *5S_RRNA* genes. From the genes mentioned above, *U1*, U6 and 5S_RRNA (Figure 4) were shared between the two statistical methods.

## 4 DISCUSSION

**FIGURE 5:**
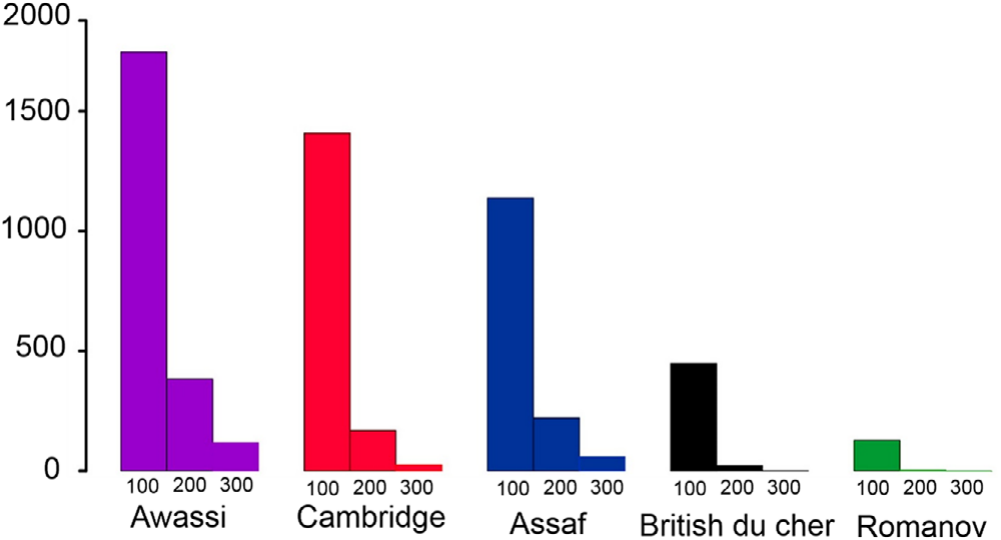
Mean number of RoHs longer than 100, 200 and 300 Kb per each population

The Cambridge breed appeared as an independent cluster for *K* values of 2–. At *K* = 3, Assaf, Awassi and British du cher breeds were clustered the same, while 75% of the Romanov population were in the separate group. At *K* = 4, the Assaf and Awassi breeds had the same cluster. In contrast, 50% of the Romanov population had the same cluster with British du cher and another 50% of the Romanov population clustered in a separate group. Analysis of genetic structure confirmed a close genetic relationship between two dairy sheep breeds, Assaf and Awassi. These results were in agreement with the fact that the Assaf sheep were created by crossing the East Friesian sheep to Awassi, which both have high milk and high lamb production capacity (Gootwine & Goot, 1996). The Cambridge breed had an independent cluster for *K* values of 2–4. It is reasonable because the Cambridge breed is a crossbred sheep from mating between Landrace rams with many local ewes in Cambridge's university. Results of admixture analysis at *K* = 3 (with the least CV error), similar to a previous study (Meadows et al., 2005), indicated that sheep have the weakest population structure and the highest intercontinental dispersal compared to other domestic animals studied to date. The frequency of ROHs across the genome can present information about the history and the management of flocks over time (Kirin et al., 2010). The mean number of RoHs above 100, 200 and 300 Kb per population is reported in Figure 5. We observed a decreasing trend from short to long lengths. Longer ROH may indicate recent inbreeding has occurred within a pedigree, while shorter segments can show the existence of more old relatedness (Bjelland et al., 2013; Kirin et al., 2010). Awassi, Assaf and Cambridge breeds had more short runs of homozygosity, while British du cher and Romanov had fewer long runs of romozygosity.. So the results of ROH analyses are indicators of the recent reduction of inbreeding in British du cher and Romanov of the other breeds.

Two genes identified simultaneously by the applied approaches, *U1* and *U6* genes, encode small nuclear RNAs (snRNAs) that play a role in RNA splicing. The 5S ribosomal RNA gene encodes 5SrRNA, a structural and functional component of the large subunit of the ribosome. It has been previously reported to be positively selected for milk traits (Taye et al., 2017).

The population differentiation analysis (FST) revealed GO terms related to different biological processes. For example, the term ‘regulation of homeostatic process’ was enriched to which genes for milk traits (*P4HTM* and *METRNL*) were involved. *P4HTM* and *METRNL* genes were formerly presented associated with the test day protein (Ibeagha‐Awemu et al., 2016) and somatic cell (Chen et al., 2015) in milk, respectively. Other genes associated with milk traits, CSN1S1 and CSN2, were found to be selection candidates (top 1% Pi). Previous studies reported that polymorphisms in these milk proteins impact milk production parameters and protein quality (Caravaca et al., 2008; Kishore et al., 2013). Casein alpha S1 protein is encoded by the *CSN1S1* gene and has an important role in the milk capacity for calcium phosphate transport. *CSN2* gene encodes beta‐casein, the main protein in milk and is the primary source of essential amino acids for infants. *ST3GAL1* gene has been previously reported to be responsible for the production of 3′‐sialylactose in colostrum and late lactation milk in goats (Crisà et al., 2016).

A polymorphism in the *SLC35A3* gene has been found to be associated with fat yield and protein production (Liu et al., 2018). It has been reported that the VPS13B gene influences total milk production, fat yield and protein production (Liu et al., 2018). *CCDC152* (Chen et al., 2015; Do et al., 2017; Zhao et al., 2015) and *NT5DC1* genes (Yodklaew et al., 2017) have previously been shown to affect lactation persistency. *OSBPL8* gene encodes Oxysterol‐binding protein‐related eight proteins, a group of intravenous lipid receptors. It is found to affect the fatty acid trait in milk (Li et al., 2014). The protein encoded by the *DPY19L1* gene has a role in transferring glycosyl groups, and a polymorphism in this gene has been reported to be associated with peak yield in dairy cattle (Yodklaew et al., 2017). *ARFGAP3* gene has been found to affect fat production (De Camargo et al., 2015).

## 5 CONCLUSION

Whole‐genome sequencing analysis is a useful method for detecting genes that functionally and globally are associated with different phenotypes in domesticated animals. The current study aimed to identify important genomic regions and genes associated with milk production traits in dairy sheep breeds, including Assaf and Awassi. The most conspicuous identified genes related to milk traits include *ST3GAL1*, *CSN1S1*, *CSN2*, *OSBPL8*, *SLC35A3*, *VPS13B*, *DPY19L1*, *CCDC152*, *NT5DC1*, *P4HTM*, *CYTH4* and *METRNL*, *U1*, *U6* and *5S_rRNA* genes. Based on the biological function of these genes in the candidate selected regions, several genes, including CSN1S1, CSN2 and SLC35A3, have possibly a major role in milk composition and production in sheep breeds. However, the identified novel candidate genes in the selected regions need to be validated in independent studies to provide a more profound insight into the genetic architecture of *Ovis aries* milk yield and composition.

## CONFLICT OF INTEREST

There is no conflict of interest for this research

## ETHICAL STATEMENT

We used the already available data and based on our institute policy in this case, no ethics review was required. Based on the policy of the animal ethics committee, Shahid Bahonar University of Kerman, this study did not require ethical approval because the study used the datasets freely available in the public.

## AUTHOR CONTRIBUTIONS

Elham Rezvannejad: conceptualisation; funding acquisition; writing – original draft; writing – review & editing. Hojjat Asadollahpour Nanaei: analysed and interpreted the data and drafted the manuscript. Ali Esmailizadeh: conceptualisation; data curation; formal analysis; methodology; project administration; supervision; writing – review & editing.

### PEER REVIEW

The peer review history for this article is available at https://publons.com/publon/10.1002/vms3.731

## Supporting information


**FIGURE S1** The number of positively selected genes detected with the two approaches listed in each Venn diagram component

**TABLE S1** Positively selected genes extracted using the FST method and output of g: profiler related to them

**TABLE S2** Positively selected genes extracted with nucleotide diversity (Pi) statistical method and output of g: profiler related to them

**TABLE S3** Gene functional enrichment categories found in positively selected genes detected by the methods Pi and FST methods

**TABLE S4** Candidate gene putatively selected by two statistical methods affecting milk production

## Data Availability

The Illumina whole‐genome sequences from Romanov sheep (accession numbers, ERR1456968, ERR1456970, ERR1456972 and ERR1456974), Assaf sheep (accession number, ERR1163920) (*n* = 1), Awassi sheep (accession number, ERR1163918), Cambridge sheep (accession numbers, ERR1419201‐ERR1419207) and British du cher sheep (accession number, ERR1456963) were downloaded from the Sequence Read Archive at the NCBI site (https://trace.ncbi.nlm.nih.gov/Traces/sra).
