# Supplementary material for: Detection of candidate genes affecting milk production traits in sheep using whole‐genome sequencing analysis
Source: Vet Med Sci. 2022 Jan 11;8(3):1197–204. doi: 10.1002/vms3.731 (PMC9122411; doi:10.1002/vms3.731)
Supplement: Supplementary file 1 — FIGURE S1 The number of positively selected genes detected with the two approaches listed in each Venn diagram component [file VMS3-8-1197-s003.docx]

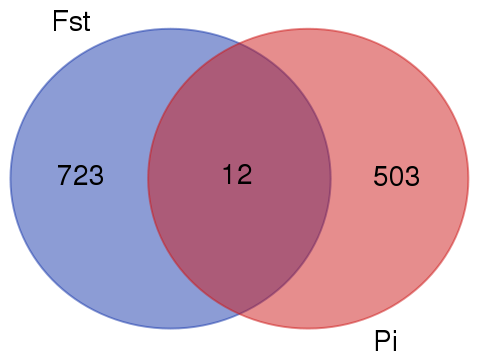


**Figure S1.** The number of positively selected genes detected with the two approaches listed in each Venn diagram component.
