# Supplementary material for: Detection of candidate genes affecting milk production traits in sheep using whole‐genome sequencing analysis
Source: Vet Med Sci. 2022 Jan 11;8(3):1197–204. doi: 10.1002/vms3.731 (PMC9122411; doi:10.1002/vms3.731)
Supplement: Supplementary file 2 — TABLE S1 Positively selected genes extracted using the FST method and output of g: profiler related to them [file VMS3-8-1197-s001.docx]

**Table S1.** Positively selected genes extracted using the FST method and output of g: profiler related to them

| # | signf | p-value | T | Q | Q&T | Q&T/Q | Q&T/T | term ID | t type | t group | t name | t depth | Q&T list |
| --- | --- | --- | --- | --- | --- | --- | --- | --- | --- | --- | --- | --- | --- |
| # |  |  |  |  |  |  |  |  |  |  |  |  |  |
| 1 | ! | 0.00947 | 301 | 541 | 26 | 0.048 | 0.086 | GO:0032844 | BP | 1 | regulation of homeostatic process | 1 | ENSOARG00000000044,ENSOARG00000002034,ENSOARG00000003466,ENSOARG00000003604,ENSOARG00000003718,ENSOARG00000005108,ENSOARG00000005847,ENSOARG00000005858,ENSOARG00000005903,ENSOARG00000010070,ENSOARG00000010142,ENSOARG00000010368,ENSOARG00000011199,ENSOARG00000011552,ENSOARG00000011759,ENSOARG00000013678,ENSOARG00000014111,ENSOARG00000015819,ENSOARG00000015842,ENSOARG00000015857,ENSOARG00000016240,ENSOARG00000016611,ENSOARG00000016668,ENSOARG00000017850,ENSOARG00000019970,ENSOARG00000020369 |
| #INFO: | PARAMETERS: fdr = 1 | |  |  |  |  |  |  |  |  |  |  |  |
| #INFO: | PARAMETERS: significant = 1 | |  |  |  |  |  |  |  |  |  |  |  |
| #INFO: | PARAMETERS: sort_by_structure = 1 | |  |  |  |  |  |  |  |  |  |  |  |
| #INFO: | PARAMETERS: user_thr = 1.00 | |  |  |  |  |  |  |  |  |  |  |  |
| #INFO: | PARAMETERS: organism = oaries | |  |  |  |  |  |  |  |  |  |  |  |
| #INFO: | User: http | |  |  |  |  |  |  |  |  |  |  |  |
| #INFO: | Host: arak-prod | |  |  |  |  |  |  |  |  |  |  |  |
| #INFO: | Time: 2017-12-11 17:3:4 | |  |  |  |  |  |  |  |  |  |  |  |
| #INFO: | Version: r1741_e90_eg37 | |  |  |  |  |  |  |  |  |  |  |  |
| #INFO: | Effective domain size for GO: 17974, threshold 0.05 | | |  |  |  |  |  |  |  |  |  |  |
| #INFO: |  |  |  |  |  |  |  |  |  |  |  |  |  |
| #INFO: | --- Gene names and descriptions | |  |  |  |  |  |  |  |  |  |  |  |
| #INFO: |  |  |  |  |  |  |  |  |  |  |  |  |  |
| #INFO: | QUERY: | ENSOARG00000000044 | IL2 | interleukin 2 [Source:HGNC Symbol;Acc:HGNC:6001] | | | | | |  |  |  |  |
| #INFO: | QUERY: | ENSOARG00000000045 | N/A | N/A |  |  |  |  |  |  |  |  |  |
| #INFO: | QUERY: | ENSOARG00000000050 | N/A | N/A |  |  |  |  |  |  |  |  |  |
| #INFO: | QUERY: | ENSOARG00000000116 | ADAD1 | adenosine deaminase domain containing 1 [Source:HGNC Symbol;Acc:HGNC:30713] | | | | | | | | |  |
| #INFO: | QUERY: | ENSOARG00000000223 | DRAXIN | dorsal inhibitory axon guidance protein [Source:HGNC Symbol;Acc:HGNC:25054] | | | | | | | |  |  |
| #INFO: | QUERY: | ENSOARG00000000224 | OTUD7A | OTU deubiquitinase 7A [Source:HGNC Symbol;Acc:HGNC:20718] | | | | | | |  |  |  |
| #INFO: | QUERY: | ENSOARG00000000238 | N/A | N/A |  |  |  |  |  |  |  |  |  |
| #INFO: | QUERY: | ENSOARG00000000290 | N/A | N/A |  |  |  |  |  |  |  |  |  |
| #INFO: | QUERY: | ENSOARG00000000316 | KIAA1109 | KIAA1109 [Source:HGNC Symbol;Acc:HGNC:26953] | | | | |  |  |  |  |  |
| #INFO: | QUERY: | ENSOARG00000000337 | MAP3K5 | mitogen-activated protein kinase kinase kinase 5 [Source:HGNC Symbol;Acc:HGNC:6857] | | | | | | | | |  |
| #INFO: | QUERY: | ENSOARG00000000401 | SETD3 | SET domain containing 3 [Source:HGNC Symbol;Acc:HGNC:20493] | | | | | | |  |  |  |
| #INFO: | QUERY: | ENSOARG00000000412 | PAPLN | papilin, proteoglycan like sulfated glycoprotein [Source:HGNC Symbol;Acc:HGNC:19262] | | | | | | | | |  |
| #INFO: | QUERY: | ENSOARG00000000430 | SLC13A1 | solute carrier family 13 member 1 [Source:HGNC Symbol;Acc:HGNC:10916] | | | | | | | |  |  |
| #INFO: | QUERY: | ENSOARG00000000438 | GRID1 | glutamate ionotropic receptor delta type subunit 1 [Source:HGNC Symbol;Acc:HGNC:4575] | | | | | | | | |  |
| #INFO: | QUERY: | ENSOARG00000000462 | NUMB | NUMB, endocytic adaptor protein [Source:HGNC Symbol;Acc:HGNC:8060] | | | | | | | |  |  |
| #INFO: | QUERY: | ENSOARG00000000495 | CCNK | cyclin K [Source:HGNC Symbol;Acc:HGNC:1596] | | | | |  |  |  |  |  |
| #INFO: | QUERY: | ENSOARG00000000569 | TNFAIP3 | TNF alpha induced protein 3 [Source:HGNC Symbol;Acc:HGNC:11896] | | | | | | |  |  |  |
| #INFO: | QUERY: | ENSOARG00000000612 | PERP | PERP, TP53 apoptosis effector [Source:HGNC Symbol;Acc:HGNC:17637] | | | | | | |  |  |  |
| #INFO: | QUERY: | ENSOARG00000000654 | MAD2L2 | mitotic arrest deficient 2 like 2 [Source:HGNC Symbol;Acc:HGNC:6764] | | | | | | |  |  |  |
| #INFO: | QUERY: | ENSOARG00000000669 | SLC25A35 | solute carrier family 25 member 35 [Source:HGNC Symbol;Acc:HGNC:31921] | | | | | | | |  |  |
| #INFO: | QUERY: | ENSOARG00000000675 | ARFGEF3 | ARFGEF family member 3 [Source:HGNC Symbol;Acc:HGNC:21213] | | | | | | |  |  |  |
| #INFO: | QUERY: | ENSOARG00000000761 | ZNF410 | zinc finger protein 410 [Source:HGNC Symbol;Acc:HGNC:20144] | | | | | | |  |  |  |
| #INFO: | QUERY: | ENSOARG00000000788 | BAIAP2L1 | BAI1 associated protein 2 like 1 [Source:HGNC Symbol;Acc:HGNC:21649] | | | | | | |  |  |  |
| #INFO: | QUERY: | ENSOARG00000000796 | FAM161B | family with sequence similarity 161 member B [Source:HGNC Symbol;Acc:HGNC:19854] | | | | | | | | |  |
| #INFO: | QUERY: | ENSOARG00000000826 | COQ6 | coenzyme Q6, monooxygenase [Source:HGNC Symbol;Acc:HGNC:20233] | | | | | | |  |  |  |
| #INFO: | QUERY: | ENSOARG00000000848 | ARHGEF15 | Rho guanine nucleotide exchange factor 15 [Source:HGNC Symbol;Acc:HGNC:15590] | | | | | | | | |  |
| #INFO: | QUERY: | ENSOARG00000000860 | ENTPD5 | ectonucleoside triphosphate diphosphohydrolase 5 [Source:HGNC Symbol;Acc:HGNC:3367] | | | | | | | | |  |
| #INFO: | QUERY: | ENSOARG00000000874 | FBXO6 | F-box protein 6 [Source:HGNC Symbol;Acc:HGNC:13585] | | | | | |  |  |  |  |
| #INFO: | QUERY: | ENSOARG00000000894 | NFYA | nuclear transcription factor Y subunit alpha [Source:HGNC Symbol;Acc:HGNC:7804] | | | | | | | |  |  |
| #INFO: | QUERY: | ENSOARG00000000898 | BBOF1 | basal body orientation factor 1 [Source:HGNC Symbol;Acc:HGNC:19855] | | | | | | |  |  |  |
| #INFO: | QUERY: | ENSOARG00000000944 | TRIM21 | tripartite motif containing 21 [Source:HGNC Symbol;Acc:HGNC:11312] | | | | | | |  |  |  |
| #INFO: | QUERY: | ENSOARG00000001044 | ODF4 | outer dense fiber of sperm tails 4 [Source:HGNC Symbol;Acc:HGNC:19056] | | | | | | | |  |  |
| #INFO: | QUERY: | ENSOARG00000001163 | N/A | N/A |  |  |  |  |  |  |  |  |  |
| #INFO: | QUERY: | ENSOARG00000001182 | SMKR1 | small lysine rich protein 1 [Source:HGNC Symbol;Acc:HGNC:43561] | | | | | | |  |  |  |
| #INFO: | QUERY: | ENSOARG00000001195 | N/A | N/A |  |  |  |  |  |  |  |  |  |
| #INFO: | QUERY: | ENSOARG00000001221 | N/A | N/A |  |  |  |  |  |  |  |  |  |
| #INFO: | QUERY: | ENSOARG00000001232 | N/A | N/A |  |  |  |  |  |  |  |  |  |
| #INFO: | QUERY: | ENSOARG00000001285 | N/A | N/A |  |  |  |  |  |  |  |  |  |
| #INFO: | QUERY: | ENSOARG00000001315 | N/A | N/A |  |  |  |  |  |  |  |  |  |
| #INFO: | QUERY: | ENSOARG00000001392 | CATHL1B | cathelicidin-1 precursor [Source:RefSeq peptide;Acc:NP_001009772] | | | | | | |  |  |  |
| #INFO: | QUERY: | ENSOARG00000001439 | TMEM266 | transmembrane protein 266 [Source:HGNC Symbol;Acc:HGNC:26763] | | | | | | |  |  |  |
| #INFO: | QUERY: | ENSOARG00000001481 | N/A | N/A |  |  |  |  |  |  |  |  |  |
| #INFO: | QUERY: | ENSOARG00000001482 | N/A | N/A |  |  |  |  |  |  |  |  |  |
| #INFO: | QUERY: | ENSOARG00000001507 | SETD6 | SET domain containing 6 [Source:HGNC Symbol;Acc:HGNC:26116] | | | | | | |  |  |  |
| #INFO: | QUERY: | ENSOARG00000001534 | PIEZO2 | piezo type mechanosensitive ion channel component 2 [Source:HGNC Symbol;Acc:HGNC:26270] | | | | | | | | | |
| #INFO: | QUERY: | ENSOARG00000001543 | N/A | N/A |  |  |  |  |  |  |  |  |  |
| #INFO: | QUERY: | ENSOARG00000001553 | N/A | N/A |  |  |  |  |  |  |  |  |  |
| #INFO: | QUERY: | ENSOARG00000001557 | MEFV | MEFV, pyrin innate immunity regulator [Source:HGNC Symbol;Acc:HGNC:6998] | | | | | | | |  |  |
| #INFO: | QUERY: | ENSOARG00000001567 | CNOT1 | CCR4-NOT transcription complex subunit 1 [Source:HGNC Symbol;Acc:HGNC:7877] | | | | | | | |  |  |
| #INFO: | QUERY: | ENSOARG00000001594 | N/A | N/A |  |  |  |  |  |  |  |  |  |
| #INFO: | QUERY: | ENSOARG00000001600 | TREML1 | triggering receptor expressed on myeloid cells like 1 [Source:HGNC Symbol;Acc:HGNC:20434] | | | | | | | | |  |
| #INFO: | QUERY: | ENSOARG00000001605 | N/A | N/A |  |  |  |  |  |  |  |  |  |
| #INFO: | QUERY: | ENSOARG00000001672 | N/A | N/A |  |  |  |  |  |  |  |  |  |
| #INFO: | QUERY: | ENSOARG00000001703 | SLC38A7 | solute carrier family 38 member 7 [Source:HGNC Symbol;Acc:HGNC:25582] | | | | | | | |  |  |
| #INFO: | QUERY: | ENSOARG00000001744 | TREM2 | triggering receptor expressed on myeloid cells 2 [Source:HGNC Symbol;Acc:HGNC:17761] | | | | | | | | |  |
| #INFO: | QUERY: | ENSOARG00000001754 | MDFIC | MyoD family inhibitor domain containing [Source:HGNC Symbol;Acc:HGNC:28870] | | | | | | | |  |  |
| #INFO: | QUERY: | ENSOARG00000001790 | TREML2 | triggering receptor expressed on myeloid cells like 2 [Source:HGNC Symbol;Acc:HGNC:21092] | | | | | | | | |  |
| #INFO: | QUERY: | ENSOARG00000001802 | CATHL3 | cathelicidin-3 precursor [Source:RefSeq peptide;Acc:NP_001009301] | | | | | | |  |  |  |
| #INFO: | QUERY: | ENSOARG00000001866 | N/A | N/A |  |  |  |  |  |  |  |  |  |
| #INFO: | QUERY: | ENSOARG00000001894 | PSMG2 | proteasome assembly chaperone 2 [Source:HGNC Symbol;Acc:HGNC:24929] | | | | | | | |  |  |
| #INFO: | QUERY: | ENSOARG00000001918 | TK2 | thymidine kinase 2, mitochondrial [Source:HGNC Symbol;Acc:HGNC:11831] | | | | | | | |  |  |
| #INFO: | QUERY: | ENSOARG00000001923 | COPS3 | COP9 signalosome subunit 3 [Source:HGNC Symbol;Acc:HGNC:2239] | | | | | | |  |  |  |
| #INFO: | QUERY: | ENSOARG00000001935 | HEPHL1 | hephaestin like 1 [Source:HGNC Symbol;Acc:HGNC:30477] | | | | | |  |  |  |  |
| #INFO: | QUERY: | ENSOARG00000001962 | CKLF | chemokine like factor [Source:HGNC Symbol;Acc:HGNC:13253] | | | | | | |  |  |  |
| #INFO: | QUERY: | ENSOARG00000001969 | BAC5 | cathelicidin-2 precursor [Source:RefSeq peptide;Acc:NP_001009787] | | | | | | |  |  |  |
| #INFO: | QUERY: | ENSOARG00000002005 | CMTM2 | CKLF like MARVEL transmembrane domain containing 2 [Source:HGNC Symbol;Acc:HGNC:19173] | | | | | | | | | |
| #INFO: | QUERY: | ENSOARG00000002034 | N/A | N/A |  |  |  |  |  |  |  |  |  |
| #INFO: | QUERY: | ENSOARG00000002043 | PLD6 | phospholipase D family member 6 [Source:HGNC Symbol;Acc:HGNC:30447] | | | | | | | |  |  |
| #INFO: | QUERY: | ENSOARG00000002045 | NR3C1 | nuclear receptor subfamily 3 group C member 1 [Source:HGNC Symbol;Acc:HGNC:7978] | | | | | | | | |  |
| #INFO: | QUERY: | ENSOARG00000002062 | N/A | N/A |  |  |  |  |  |  |  |  |  |
| #INFO: | QUERY: | ENSOARG00000002081 | MAST2 | microtubule associated serine/threonine kinase 2 [Source:HGNC Symbol;Acc:HGNC:19035] | | | | | | | | |  |
| #INFO: | QUERY: | ENSOARG00000002144 | LDLRAD4 | low density lipoprotein receptor class A domain containing 4 [Source:HGNC Symbol;Acc:HGNC:1224] | | | | | | | | | |
| #INFO: | QUERY: | ENSOARG00000002180 | N/A | N/A |  |  |  |  |  |  |  |  |  |
| #INFO: | QUERY: | ENSOARG00000002183 | FAM210A | family with sequence similarity 210 member A [Source:HGNC Symbol;Acc:HGNC:28346] | | | | | | | | |  |
| #INFO: | QUERY: | ENSOARG00000002190 | N/A | N/A |  |  |  |  |  |  |  |  |  |
| #INFO: | QUERY: | ENSOARG00000002195 | RNMT | RNA guanine-7 methyltransferase [Source:HGNC Symbol;Acc:HGNC:10075] | | | | | | | |  |  |
| #INFO: | QUERY: | ENSOARG00000002206 | MPRIP | myosin phosphatase Rho interacting protein [Source:HGNC Symbol;Acc:HGNC:30321] | | | | | | | | |  |
| #INFO: | QUERY: | ENSOARG00000002215 | CDC42BPA | CDC42 binding protein kinase alpha [Source:HGNC Symbol;Acc:HGNC:1737] | | | | | | | |  |  |
| #INFO: | QUERY: | ENSOARG00000002239 | MC5R | Ovis aries melanocortin 5 receptor (MC5R), mRNA. [Source:RefSeq mRNA;Acc:NM_001078656] | | | | | | | | | |
| #INFO: | QUERY: | ENSOARG00000002259 | COASY | Coenzyme A synthase [Source:HGNC Symbol;Acc:HGNC:29932] | | | | | | |  |  |  |
| #INFO: | QUERY: | ENSOARG00000002269 | IRF2BPL | interferon regulatory factor 2 binding protein like [Source:HGNC Symbol;Acc:HGNC:14282] | | | | | | | | |  |
| #INFO: | QUERY: | ENSOARG00000002277 | N/A | N/A |  |  |  |  |  |  |  |  |  |
| #INFO: | QUERY: | ENSOARG00000002300 | VPS13B | vacuolar protein sorting 13 homolog B [Source:HGNC Symbol;Acc:HGNC:2183] | | | | | | | |  |  |
| #INFO: | QUERY: | ENSOARG00000002337 | N/A | N/A |  |  |  |  |  |  |  |  |  |
| #INFO: | QUERY: | ENSOARG00000002343 | MLX | MLX, MAX dimerization protein [Source:HGNC Symbol;Acc:HGNC:11645] | | | | | | |  |  |  |
| #INFO: | QUERY: | ENSOARG00000002369 | PSMC3IP | PSMC3 interacting protein [Source:HGNC Symbol;Acc:HGNC:17928] | | | | | | |  |  |  |
| #INFO: | QUERY: | ENSOARG00000002388 | N/A | N/A |  |  |  |  |  |  |  |  |  |
| #INFO: | QUERY: | ENSOARG00000002485 | SSNA1 | SS nuclear autoantigen 1 [Source:HGNC Symbol;Acc:HGNC:11321] | | | | | | |  |  |  |
| #INFO: | QUERY: | ENSOARG00000002508 | N/A | N/A |  |  |  |  |  |  |  |  |  |
| #INFO: | QUERY: | ENSOARG00000002519 | N/A | N/A |  |  |  |  |  |  |  |  |  |
| #INFO: | QUERY: | ENSOARG00000002561 | CFAP61 | cilia and flagella associated protein 61 [Source:HGNC Symbol;Acc:HGNC:15872] | | | | | | | |  |  |
| #INFO: | QUERY: | ENSOARG00000002610 | RETREG3 | reticulophagy regulator family member 3 [Source:HGNC Symbol;Acc:HGNC:27258] | | | | | | | |  |  |
| #INFO: | QUERY: | ENSOARG00000002687 | TUBG2 | tubulin gamma 2 [Source:HGNC Symbol;Acc:HGNC:12419] | | | | | |  |  |  |  |
| #INFO: | QUERY: | ENSOARG00000002799 | IRAK3 | interleukin 1 receptor associated kinase 3 [Source:HGNC Symbol;Acc:HGNC:17020] | | | | | | | |  |  |
| #INFO: | QUERY: | ENSOARG00000002818 | N/A | N/A |  |  |  |  |  |  |  |  |  |
| #INFO: | QUERY: | ENSOARG00000002819 | ALOX5 | arachidonate 5-lipoxygenase [Source:HGNC Symbol;Acc:HGNC:435] | | | | | | |  |  |  |
| #INFO: | QUERY: | ENSOARG00000002858 | OR13A1 | olfactory receptor family 13 subfamily A member 1 [Source:HGNC Symbol;Acc:HGNC:14772] | | | | | | | | |  |
| #INFO: | QUERY: | ENSOARG00000002903 | N/A | N/A |  |  |  |  |  |  |  |  |  |
| #INFO: | QUERY: | ENSOARG00000002919 | LLPH | LLP homolog, long-term synaptic facilitation [Source:HGNC Symbol;Acc:HGNC:28229] | | | | | | | | |  |
| #INFO: | QUERY: | ENSOARG00000003093 | N/A | N/A |  |  |  |  |  |  |  |  |  |
| #INFO: | QUERY: | ENSOARG00000003154 | ARHGAP21 | Rho GTPase activating protein 21 [Source:HGNC Symbol;Acc:HGNC:23725] | | | | | | | |  |  |
| #INFO: | QUERY: | ENSOARG00000003183 | N/A | N/A |  |  |  |  |  |  |  |  |  |
| #INFO: | QUERY: | ENSOARG00000003203 | GUCA1A | guanylate cyclase activator 1A [Source:HGNC Symbol;Acc:HGNC:4678] | | | | | | |  |  |  |
| #INFO: | QUERY: | ENSOARG00000003210 | CCL25 | C-C motif chemokine 25 precursor [Source:RefSeq peptide;Acc:NP_001035380] | | | | | | | |  |  |
| #INFO: | QUERY: | ENSOARG00000003223 | GUCA1B | guanylate cyclase activator 1B [Source:HGNC Symbol;Acc:HGNC:4679] | | | | | | |  |  |  |
| #INFO: | QUERY: | ENSOARG00000003226 | N/A | N/A |  |  |  |  |  |  |  |  |  |
| #INFO: | QUERY: | ENSOARG00000003276 | N/A | N/A |  |  |  |  |  |  |  |  |  |
| #INFO: | QUERY: | ENSOARG00000003277 | MRPS10 | mitochondrial ribosomal protein S10 [Source:HGNC Symbol;Acc:HGNC:14502] | | | | | | | |  |  |
| #INFO: | QUERY: | ENSOARG00000003342 | NFATC3 | nuclear factor of activated T-cells 3 [Source:HGNC Symbol;Acc:HGNC:7777] | | | | | | | |  |  |
| #INFO: | QUERY: | ENSOARG00000003360 | ESRP2 | epithelial splicing regulatory protein 2 [Source:HGNC Symbol;Acc:HGNC:26152] | | | | | | | |  |  |
| #INFO: | QUERY: | ENSOARG00000003361 | N/A | N/A |  |  |  |  |  |  |  |  |  |
| #INFO: | QUERY: | ENSOARG00000003375 | N/A | N/A |  |  |  |  |  |  |  |  |  |
| #INFO: | QUERY: | ENSOARG00000003389 | SLC7A6 | solute carrier family 7 member 6 [Source:HGNC Symbol;Acc:HGNC:11064] | | | | | | | |  |  |
| #INFO: | QUERY: | ENSOARG00000003397 | SLC7A6OS | solute carrier family 7 member 6 opposite strand [Source:HGNC Symbol;Acc:HGNC:25807] | | | | | | | | |  |
| #INFO: | QUERY: | ENSOARG00000003400 | RALGAPA2 | Ral GTPase activating protein catalytic alpha subunit 2 [Source:HGNC Symbol;Acc:HGNC:16207] | | | | | | | | | |
| #INFO: | QUERY: | ENSOARG00000003409 | PRMT7 | protein arginine methyltransferase 7 [Source:HGNC Symbol;Acc:HGNC:25557] | | | | | | | |  |  |
| #INFO: | QUERY: | ENSOARG00000003422 | SMPD3 | sphingomyelin phosphodiesterase 3 [Source:HGNC Symbol;Acc:HGNC:14240] | | | | | | | |  |  |
| #INFO: | QUERY: | ENSOARG00000003428 | FBN3 | fibrillin 3 [Source:HGNC Symbol;Acc:HGNC:18794] | | | | |  |  |  |  |  |
| #INFO: | QUERY: | ENSOARG00000003466 | N/A | N/A |  |  |  |  |  |  |  |  |  |
| #INFO: | QUERY: | ENSOARG00000003492 | CALM1 | calmodulin 1 [Source:HGNC Symbol;Acc:HGNC:1442] | | | | | |  |  |  |  |
| #INFO: | QUERY: | ENSOARG00000003532 | C16ORF96 | chromosome 16 open reading frame 96 [Source:HGNC Symbol;Acc:HGNC:40031] | | | | | | | |  |  |
| #INFO: | QUERY: | ENSOARG00000003599 | N/A | N/A |  |  |  |  |  |  |  |  |  |
| #INFO: | QUERY: | ENSOARG00000003604 | CORO1A | coronin 1A [Source:HGNC Symbol;Acc:HGNC:2252] | | | | |  |  |  |  |  |
| #INFO: | QUERY: | ENSOARG00000003612 | ABCC8 | ATP binding cassette subfamily C member 8 [Source:HGNC Symbol;Acc:HGNC:59] | | | | | | | |  |  |
| #INFO: | QUERY: | ENSOARG00000003653 | MGRN1 | mahogunin ring finger 1 [Source:HGNC Symbol;Acc:HGNC:20254] | | | | | | |  |  |  |
| #INFO: | QUERY: | ENSOARG00000003703 | AOAH | acyloxyacyl hydrolase [Source:HGNC Symbol;Acc:HGNC:548] | | | | | |  |  |  |  |
| #INFO: | QUERY: | ENSOARG00000003718 | MAPK3 | mitogen-activated protein kinase 3 [Source:HGNC Symbol;Acc:HGNC:6877] | | | | | | | |  |  |
| #INFO: | QUERY: | ENSOARG00000003771 | GDPD3 | glycerophosphodiester phosphodiesterase domain containing 3 [Source:HGNC Symbol;Acc:HGNC:28638] | | | | | | | | | |
| #INFO: | QUERY: | ENSOARG00000003779 | NCOA2 | nuclear receptor coactivator 2 [Source:HGNC Symbol;Acc:HGNC:7669] | | | | | | |  |  |  |
| #INFO: | QUERY: | ENSOARG00000003882 | YPEL3 | yippee like 3 [Source:HGNC Symbol;Acc:HGNC:18327] | | | | | |  |  |  |  |
| #INFO: | QUERY: | ENSOARG00000003884 | N/A | N/A |  |  |  |  |  |  |  |  |  |
| #INFO: | QUERY: | ENSOARG00000003950 | MC2R | adrenocorticotropic hormone receptor [Source:RefSeq peptide;Acc:NP_001009442] | | | | | | | | |  |
| #INFO: | QUERY: | ENSOARG00000003979 | RABEP1 | rabaptin, RAB GTPase binding effector protein 1 [Source:HGNC Symbol;Acc:HGNC:17677] | | | | | | | | |  |
| #INFO: | QUERY: | ENSOARG00000004116 | KIZ | kizuna centrosomal protein [Source:HGNC Symbol;Acc:HGNC:15865] | | | | | | |  |  |  |
| #INFO: | QUERY: | ENSOARG00000004144 | N/A | N/A |  |  |  |  |  |  |  |  |  |
| #INFO: | QUERY: | ENSOARG00000004243 | SDCCAG3 | serologically defined colon cancer antigen 3 [Source:HGNC Symbol;Acc:HGNC:10667] | | | | | | | | |  |
| #INFO: | QUERY: | ENSOARG00000004245 | ARID1B | AT-rich interaction domain 1B [Source:HGNC Symbol;Acc:HGNC:18040] | | | | | | |  |  |  |
| #INFO: | QUERY: | ENSOARG00000004287 | TOPAZ1 | testis and ovary specific PAZ domain containing 1 [Source:HGNC Symbol;Acc:HGNC:24746] | | | | | | | | |  |
| #INFO: | QUERY: | ENSOARG00000004351 | N/A | N/A |  |  |  |  |  |  |  |  |  |
| #INFO: | QUERY: | ENSOARG00000004360 | SNAPC4 | small nuclear RNA activating complex polypeptide 4 [Source:HGNC Symbol;Acc:HGNC:11137] | | | | | | | | |  |
| #INFO: | QUERY: | ENSOARG00000004412 | N/A | N/A |  |  |  |  |  |  |  |  |  |
| #INFO: | QUERY: | ENSOARG00000004443 | XRN2 | 5'-3' exoribonuclease 2 [Source:HGNC Symbol;Acc:HGNC:12836] | | | | | | |  |  |  |
| #INFO: | QUERY: | ENSOARG00000004481 | N/A | N/A |  |  |  |  |  |  |  |  |  |
| #INFO: | QUERY: | ENSOARG00000004489 | EGR2 | early growth response 2 [Source:HGNC Symbol;Acc:HGNC:3239] | | | | | | |  |  |  |
| #INFO: | QUERY: | ENSOARG00000004519 | CARD9 | caspase recruitment domain family member 9 [Source:HGNC Symbol;Acc:HGNC:16391] | | | | | | | | |  |
| #INFO: | QUERY: | ENSOARG00000004532 | N/A | N/A |  |  |  |  |  |  |  |  |  |
| #INFO: | QUERY: | ENSOARG00000004552 | GPSM1 | G protein signaling modulator 1 [Source:HGNC Symbol;Acc:HGNC:17858] | | | | | | |  |  |  |
| #INFO: | QUERY: | ENSOARG00000004769 | N/A | N/A |  |  |  |  |  |  |  |  |  |
| #INFO: | QUERY: | ENSOARG00000004777 | FAM217B | family with sequence similarity 217 member B [Source:HGNC Symbol;Acc:HGNC:16170] | | | | | | | | |  |
| #INFO: | QUERY: | ENSOARG00000004792 | PPP1R3D | protein phosphatase 1 regulatory subunit 3D [Source:HGNC Symbol;Acc:HGNC:9294] | | | | | | | | |  |
| #INFO: | QUERY: | ENSOARG00000004869 | UTP15 | UTP15, small subunit processome component [Source:HGNC Symbol;Acc:HGNC:25758] | | | | | | | | |  |
| #INFO: | QUERY: | ENSOARG00000004876 | NKX2-2 | NK2 homeobox 2 [Source:HGNC Symbol;Acc:HGNC:7835] | | | | | |  |  |  |  |
| #INFO: | QUERY: | ENSOARG00000004890 | N/A | N/A |  |  |  |  |  |  |  |  |  |
| #INFO: | QUERY: | ENSOARG00000004900 | N/A | N/A |  |  |  |  |  |  |  |  |  |
| #INFO: | QUERY: | ENSOARG00000004903 | ANKRA2 | ankyrin repeat family A member 2 [Source:HGNC Symbol;Acc:HGNC:13208] | | | | | | | |  |  |
| #INFO: | QUERY: | ENSOARG00000004908 | NRDC | nardilysin convertase [Source:HGNC Symbol;Acc:HGNC:7995] | | | | | |  |  |  |  |
| #INFO: | QUERY: | ENSOARG00000004927 | SEPT7 | septin 7 [Source:HGNC Symbol;Acc:HGNC:1717] | | | | |  |  |  |  |  |
| #INFO: | QUERY: | ENSOARG00000004955 | PTPRA | protein tyrosine phosphatase, receptor type A [Source:HGNC Symbol;Acc:HGNC:9664] | | | | | | | | |  |
| #INFO: | QUERY: | ENSOARG00000004967 | DOK6 | docking protein 6 [Source:HGNC Symbol;Acc:HGNC:28301] | | | | | |  |  |  |  |
| #INFO: | QUERY: | ENSOARG00000005108 | DLL1 | delta like canonical Notch ligand 1 [Source:HGNC Symbol;Acc:HGNC:2908] | | | | | | | |  |  |
| #INFO: | QUERY: | ENSOARG00000005122 | FAM120B | family with sequence similarity 120B [Source:HGNC Symbol;Acc:HGNC:21109] | | | | | | | |  |  |
| #INFO: | QUERY: | ENSOARG00000005131 | PSMB1 | proteasome subunit beta 1 [Source:HGNC Symbol;Acc:HGNC:9537] | | | | | | |  |  |  |
| #INFO: | QUERY: | ENSOARG00000005134 | N/A | N/A |  |  |  |  |  |  |  |  |  |
| #INFO: | QUERY: | ENSOARG00000005230 | STOX1 | storkhead box 1 [Source:HGNC Symbol;Acc:HGNC:23508] | | | | | |  |  |  |  |
| #INFO: | QUERY: | ENSOARG00000005264 | DDX50 | DExD-box helicase 50 [Source:HGNC Symbol;Acc:HGNC:17906] | | | | | |  |  |  |  |
| #INFO: | QUERY: | ENSOARG00000005460 | DDB2 | damage specific DNA binding protein 2 [Source:HGNC Symbol;Acc:HGNC:2718] | | | | | | | |  |  |
| #INFO: | QUERY: | ENSOARG00000005603 | KHDRBS2 | KH RNA binding domain containing, signal transduction associated 2 [Source:HGNC Symbol;Acc:HGNC:18114] | | | | | | | | | |
| #INFO: | QUERY: | ENSOARG00000005614 | ACP2 | acid phosphatase 2, lysosomal [Source:HGNC Symbol;Acc:HGNC:123] | | | | | | |  |  |  |
| #INFO: | QUERY: | ENSOARG00000005711 | COL13A1 | collagen type XIII alpha 1 chain [Source:HGNC Symbol;Acc:HGNC:2190] | | | | | | |  |  |  |
| #INFO: | QUERY: | ENSOARG00000005715 | DPY19L1 | dpy-19 like 1 [Source:HGNC Symbol;Acc:HGNC:22205] | | | | | |  |  |  |  |
| #INFO: | QUERY: | ENSOARG00000005733 | CDK5RAP2 | CDK5 regulatory subunit associated protein 2 [Source:HGNC Symbol;Acc:HGNC:18672] | | | | | | | | |  |
| #INFO: | QUERY: | ENSOARG00000005762 | TMEM144 | transmembrane protein 144 [Source:HGNC Symbol;Acc:HGNC:25633] | | | | | | |  |  |  |
| #INFO: | QUERY: | ENSOARG00000005801 | SLC25A41 | solute carrier family 25 member 41 [Source:HGNC Symbol;Acc:HGNC:28533] | | | | | | | |  |  |
| #INFO: | QUERY: | ENSOARG00000005819 | SOX6 | SRY-box 6 [Source:HGNC Symbol;Acc:HGNC:16421] | | | | |  |  |  |  |  |
| #INFO: | QUERY: | ENSOARG00000005847 | PRKCE | protein kinase C epsilon [Source:HGNC Symbol;Acc:HGNC:9401] | | | | | | |  |  |  |
| #INFO: | QUERY: | ENSOARG00000005858 | NR1H3 | nuclear receptor subfamily 1 group H member 3 [Source:HGNC Symbol;Acc:HGNC:7966] | | | | | | | | |  |
| #INFO: | QUERY: | ENSOARG00000005870 | PIK3R1 | phosphoinositide-3-kinase regulatory subunit 1 [Source:HGNC Symbol;Acc:HGNC:8979] | | | | | | | | |  |
| #INFO: | QUERY: | ENSOARG00000005881 | KHSRP | KH-type splicing regulatory protein [Source:HGNC Symbol;Acc:HGNC:6316] | | | | | | | |  |  |
| #INFO: | QUERY: | ENSOARG00000005903 | NPSR1 | neuropeptide S receptor 1 [Source:HGNC Symbol;Acc:HGNC:23631] | | | | | | |  |  |  |
| #INFO: | QUERY: | ENSOARG00000005904 | CCDC178 | coiled-coil domain containing 178 [Source:HGNC Symbol;Acc:HGNC:29588] | | | | | | | |  |  |
| #INFO: | QUERY: | ENSOARG00000005922 | PPA1 | pyrophosphatase (inorganic) 1 [Source:HGNC Symbol;Acc:HGNC:9226] | | | | | | |  |  |  |
| #INFO: | QUERY: | ENSOARG00000005957 | N/A | N/A |  |  |  |  |  |  |  |  |  |
| #INFO: | QUERY: | ENSOARG00000005988 | BPIFB4 | BPI fold containing family B member 4 [Source:HGNC Symbol;Acc:HGNC:16179] | | | | | | | |  |  |
| #INFO: | QUERY: | ENSOARG00000005998 | MADD | MAP kinase activating death domain [Source:HGNC Symbol;Acc:HGNC:6766] | | | | | | | |  |  |
| #INFO: | QUERY: | ENSOARG00000006011 | STAU2 | staufen double-stranded RNA binding protein 2 [Source:HGNC Symbol;Acc:HGNC:11371] | | | | | | | | |  |
| #INFO: | QUERY: | ENSOARG00000006018 | NPFFR1 | neuropeptide FF receptor 1 [Source:HGNC Symbol;Acc:HGNC:17425] | | | | | | |  |  |  |
| #INFO: | QUERY: | ENSOARG00000006051 | GTF2F1 | general transcription factor IIF subunit 1 [Source:HGNC Symbol;Acc:HGNC:4652] | | | | | | | |  |  |
| #INFO: | QUERY: | ENSOARG00000006054 | N/A | N/A |  |  |  |  |  |  |  |  |  |
| #INFO: | QUERY: | ENSOARG00000006067 | TMEM57 | transmembrane protein 57 [Source:HGNC Symbol;Acc:HGNC:25572] | | | | | | |  |  |  |
| #INFO: | QUERY: | ENSOARG00000006125 | N/A | N/A |  |  |  |  |  |  |  |  |  |
| #INFO: | QUERY: | ENSOARG00000006130 | N/A | N/A |  |  |  |  |  |  |  |  |  |
| #INFO: | QUERY: | ENSOARG00000006147 | PSPN | persephin [Source:HGNC Symbol;Acc:HGNC:9579] | | | | |  |  |  |  |  |
| #INFO: | QUERY: | ENSOARG00000006148 | N/A | N/A |  |  |  |  |  |  |  |  |  |
| #INFO: | QUERY: | ENSOARG00000006150 | N/A | N/A |  |  |  |  |  |  |  |  |  |
| #INFO: | QUERY: | ENSOARG00000006163 | BBS9 | Bardet-Biedl syndrome 9 [Source:HGNC Symbol;Acc:HGNC:30000] | | | | | | |  |  |  |
| #INFO: | QUERY: | ENSOARG00000006195 | ALKBH7 | alkB homolog 7 [Source:HGNC Symbol;Acc:HGNC:21306] | | | | | |  |  |  |  |
| #INFO: | QUERY: | ENSOARG00000006219 | TRAPPC8 | trafficking protein particle complex 8 [Source:HGNC Symbol;Acc:HGNC:29169] | | | | | | | |  |  |
| #INFO: | QUERY: | ENSOARG00000006233 | CLPP | caseinolytic mitochondrial matrix peptidase proteolytic subunit [Source:HGNC Symbol;Acc:HGNC:2084] | | | | | | | | | |
| #INFO: | QUERY: | ENSOARG00000006261 | LRP8 | LDL receptor related protein 8 [Source:HGNC Symbol;Acc:HGNC:6700] | | | | | | |  |  |  |
| #INFO: | QUERY: | ENSOARG00000006290 | N/A | N/A |  |  |  |  |  |  |  |  |  |
| #INFO: | QUERY: | ENSOARG00000006357 | NUBPL | nucleotide binding protein like [Source:HGNC Symbol;Acc:HGNC:20278] | | | | | | |  |  |  |
| #INFO: | QUERY: | ENSOARG00000006373 | CLEC16A | C-type lectin domain containing 16A [Source:HGNC Symbol;Acc:HGNC:29013] | | | | | | | |  |  |
| #INFO: | QUERY: | ENSOARG00000006414 | DIAPH3 | diaphanous related formin 3 [Source:HGNC Symbol;Acc:HGNC:15480] | | | | | | |  |  |  |
| #INFO: | QUERY: | ENSOARG00000006489 | RFX2 | regulatory factor X2 [Source:HGNC Symbol;Acc:HGNC:9983] | | | | | |  |  |  |  |
| #INFO: | QUERY: | ENSOARG00000006541 | LONP1 | lon peptidase 1, mitochondrial [Source:HGNC Symbol;Acc:HGNC:9479] | | | | | | |  |  |  |
| #INFO: | QUERY: | ENSOARG00000006628 | N/A | N/A |  |  |  |  |  |  |  |  |  |
| #INFO: | QUERY: | ENSOARG00000006661 | N/A | N/A |  |  |  |  |  |  |  |  |  |
| #INFO: | QUERY: | ENSOARG00000006759 | N/A | N/A |  |  |  |  |  |  |  |  |  |
| #INFO: | QUERY: | ENSOARG00000006856 | ZCCHC4 | zinc finger CCHC-type containing 4 [Source:HGNC Symbol;Acc:HGNC:22917] | | | | | | | |  |  |
| #INFO: | QUERY: | ENSOARG00000006876 | BAZ1A | bromodomain adjacent to zinc finger domain 1A [Source:HGNC Symbol;Acc:HGNC:960] | | | | | | | | |  |
| #INFO: | QUERY: | ENSOARG00000006883 | VSIR | V-set immunoregulatory receptor [Source:HGNC Symbol;Acc:HGNC:30085] | | | | | | | |  |  |
| #INFO: | QUERY: | ENSOARG00000006951 | CAMK2A | calcium/calmodulin dependent protein kinase II alpha [Source:HGNC Symbol;Acc:HGNC:1460] | | | | | | | | | |
| #INFO: | QUERY: | ENSOARG00000006957 | PSAP | prosaposin [Source:HGNC Symbol;Acc:HGNC:9498] | | | | |  |  |  |  |  |
| #INFO: | QUERY: | ENSOARG00000006995 | FCHSD2 | FCH and double SH3 domains 2 [Source:HGNC Symbol;Acc:HGNC:29114] | | | | | | |  |  |  |
| #INFO: | QUERY: | ENSOARG00000007172 | ANAPC4 | anaphase promoting complex subunit 4 [Source:HGNC Symbol;Acc:HGNC:19990] | | | | | | | |  |  |
| #INFO: | QUERY: | ENSOARG00000007198 | N/A | N/A |  |  |  |  |  |  |  |  |  |
| #INFO: | QUERY: | ENSOARG00000007271 | HAAO | 3-hydroxyanthranilate 3,4-dioxygenase [Source:HGNC Symbol;Acc:HGNC:4796] | | | | | | | |  |  |
| #INFO: | QUERY: | ENSOARG00000007346 | CEP70 | centrosomal protein 70 [Source:HGNC Symbol;Acc:HGNC:29972] | | | | | | |  |  |  |
| #INFO: | QUERY: | ENSOARG00000007373 | N/A | N/A |  |  |  |  |  |  |  |  |  |
| #INFO: | QUERY: | ENSOARG00000007397 | FAR1 | fatty acyl-CoA reductase 1 [Source:HGNC Symbol;Acc:HGNC:26222] | | | | | | |  |  |  |
| #INFO: | QUERY: | ENSOARG00000007415 | N/A | N/A |  |  |  |  |  |  |  |  |  |
| #INFO: | QUERY: | ENSOARG00000007431 | N/A | N/A |  |  |  |  |  |  |  |  |  |
| #INFO: | QUERY: | ENSOARG00000007445 | RAD18 | RAD18, E3 ubiquitin protein ligase [Source:HGNC Symbol;Acc:HGNC:18278] | | | | | | | |  |  |
| #INFO: | QUERY: | ENSOARG00000007450 | N/A | N/A |  |  |  |  |  |  |  |  |  |
| #INFO: | QUERY: | ENSOARG00000007463 | N/A | N/A |  |  |  |  |  |  |  |  |  |
| #INFO: | QUERY: | ENSOARG00000007466 | N/A | N/A |  |  |  |  |  |  |  |  |  |
| #INFO: | QUERY: | ENSOARG00000007479 | N/A | N/A |  |  |  |  |  |  |  |  |  |
| #INFO: | QUERY: | ENSOARG00000007536 | N/A | N/A |  |  |  |  |  |  |  |  |  |
| #INFO: | QUERY: | ENSOARG00000007572 | N/A | N/A |  |  |  |  |  |  |  |  |  |
| #INFO: | QUERY: | ENSOARG00000007583 | N/A | N/A |  |  |  |  |  |  |  |  |  |
| #INFO: | QUERY: | ENSOARG00000007623 | GCSAML | germinal center associated signaling and motility like [Source:HGNC Symbol;Acc:HGNC:29583] | | | | | | | | | |
| #INFO: | QUERY: | ENSOARG00000007625 | IL10RA | interleukin 10 receptor subunit alpha [Source:HGNC Symbol;Acc:HGNC:5964] | | | | | | | |  |  |
| #INFO: | QUERY: | ENSOARG00000007748 | TMPRSS4-AS1 | TMPRSS4 antisense RNA 1 [Source:HGNC Symbol;Acc:HGNC:44179] | | | | | | |  |  |  |
| #INFO: | QUERY: | ENSOARG00000007781 | MEGF8 | multiple EGF like domains 8 [Source:HGNC Symbol;Acc:HGNC:3233] | | | | | | |  |  |  |
| #INFO: | QUERY: | ENSOARG00000007819 | CALD1 | caldesmon 1 [Source:HGNC Symbol;Acc:HGNC:1441] | | | | | |  |  |  |  |
| #INFO: | QUERY: | ENSOARG00000007843 | N/A | N/A |  |  |  |  |  |  |  |  |  |
| #INFO: | QUERY: | ENSOARG00000007870 | SPTLC1 | serine palmitoyltransferase long chain base subunit 1 [Source:HGNC Symbol;Acc:HGNC:11277] | | | | | | | | | |
| #INFO: | QUERY: | ENSOARG00000007898 | PRR19 | proline rich 19 [Source:HGNC Symbol;Acc:HGNC:33728] | | | | | |  |  |  |  |
| #INFO: | QUERY: | ENSOARG00000007904 | TMPRSS4 | transmembrane protease, serine 4 [Source:HGNC Symbol;Acc:HGNC:11878] | | | | | | | |  |  |
| #INFO: | QUERY: | ENSOARG00000007907 | SEC14L6 | SEC14 like lipid binding 6 [Source:HGNC Symbol;Acc:HGNC:40047] | | | | | | |  |  |  |
| #INFO: | QUERY: | ENSOARG00000007926 | PAFAH1B3 | platelet activating factor acetylhydrolase 1b catalytic subunit 3 [Source:HGNC Symbol;Acc:HGNC:8576] | | | | | | | | | |
| #INFO: | QUERY: | ENSOARG00000007980 | MSL2 | MSL complex subunit 2 [Source:HGNC Symbol;Acc:HGNC:25544] | | | | | | |  |  |  |
| #INFO: | QUERY: | ENSOARG00000007985 | N/A | N/A |  |  |  |  |  |  |  |  |  |
| #INFO: | QUERY: | ENSOARG00000007998 | DGKB | diacylglycerol kinase beta [Source:HGNC Symbol;Acc:HGNC:2850] | | | | | | |  |  |  |
| #INFO: | QUERY: | ENSOARG00000008005 | N/A | N/A |  |  |  |  |  |  |  |  |  |
| #INFO: | QUERY: | ENSOARG00000008030 | PPP2R3A | protein phosphatase 2 regulatory subunit B''alpha [Source:HGNC Symbol;Acc:HGNC:9307] | | | | | | | | |  |
| #INFO: | QUERY: | ENSOARG00000008110 | N/A | N/A |  |  |  |  |  |  |  |  |  |
| #INFO: | QUERY: | ENSOARG00000008184 | N/A | N/A |  |  |  |  |  |  |  |  |  |
| #INFO: | QUERY: | ENSOARG00000008189 | N/A | N/A |  |  |  |  |  |  |  |  |  |
| #INFO: | QUERY: | ENSOARG00000008190 | BICD2 | BICD cargo adaptor 2 [Source:HGNC Symbol;Acc:HGNC:17208] | | | | | |  |  |  |  |
| #INFO: | QUERY: | ENSOARG00000008204 | IPPK | inositol-pentakisphosphate 2-kinase [Source:HGNC Symbol;Acc:HGNC:14645] | | | | | | | |  |  |
| #INFO: | QUERY: | ENSOARG00000008212 | ITGA2 | integrin subunit alpha 2 [Source:HGNC Symbol;Acc:HGNC:6137] | | | | | | |  |  |  |
| #INFO: | QUERY: | ENSOARG00000008214 | PES1 | pescadillo ribosomal biogenesis factor 1 [Source:HGNC Symbol;Acc:HGNC:8848] | | | | | | | |  |  |
| #INFO: | QUERY: | ENSOARG00000008230 | ITM2B | integral membrane protein 2B [Source:HGNC Symbol;Acc:HGNC:6174] | | | | | | |  |  |  |
| #INFO: | QUERY: | ENSOARG00000008273 | N/A | N/A |  |  |  |  |  |  |  |  |  |
| #INFO: | QUERY: | ENSOARG00000008362 | N/A | N/A |  |  |  |  |  |  |  |  |  |
| #INFO: | QUERY: | ENSOARG00000008373 | FAM240B | family with sequence similarity 240 member B [Source:HGNC Symbol;Acc:HGNC:53430] | | | | | | | | |  |
| #INFO: | QUERY: | ENSOARG00000008451 | PRR15 | proline rich 15 [Source:HGNC Symbol;Acc:HGNC:22310] | | | | | |  |  |  |  |
| #INFO: | QUERY: | ENSOARG00000008487 | SETDB2 | SET domain bifurcated 2 [Source:HGNC Symbol;Acc:HGNC:20263] | | | | | | |  |  |  |
| #INFO: | QUERY: | ENSOARG00000008531 | N/A | N/A |  |  |  |  |  |  |  |  |  |
| #INFO: | QUERY: | ENSOARG00000008578 | PTCH1 | patched 1 [Source:HGNC Symbol;Acc:HGNC:9585] | | | | |  |  |  |  |  |
| #INFO: | QUERY: | ENSOARG00000008593 | N/A | N/A |  |  |  |  |  |  |  |  |  |
| #INFO: | QUERY: | ENSOARG00000008739 | DCBLD1 | discoidin, CUB and LCCL domain containing 1 [Source:HGNC Symbol;Acc:HGNC:21479] | | | | | | | | |  |
| #INFO: | QUERY: | ENSOARG00000008775 | N/A | N/A |  |  |  |  |  |  |  |  |  |
| #INFO: | QUERY: | ENSOARG00000008801 | CCDC152 | coiled-coil domain containing 152 [Source:HGNC Symbol;Acc:HGNC:34438] | | | | | | | |  |  |
| #INFO: | QUERY: | ENSOARG00000008903 | CHN2 | chimerin 2 [Source:HGNC Symbol;Acc:HGNC:1944] | | | | |  |  |  |  |  |
| #INFO: | QUERY: | ENSOARG00000008923 | OXCT1 | 3-oxoacid CoA-transferase 1 [Source:HGNC Symbol;Acc:HGNC:8527] | | | | | | |  |  |  |
| #INFO: | QUERY: | ENSOARG00000008934 | TSPAN13 | tetraspanin 13 [Source:HGNC Symbol;Acc:HGNC:21643] | | | | | |  |  |  |  |
| #INFO: | QUERY: | ENSOARG00000008996 | N/A | N/A |  |  |  |  |  |  |  |  |  |
| #INFO: | QUERY: | ENSOARG00000009105 | N/A | N/A |  |  |  |  |  |  |  |  |  |
| #INFO: | QUERY: | ENSOARG00000009262 | CARD6 | caspase recruitment domain family member 6 [Source:HGNC Symbol;Acc:HGNC:16394] | | | | | | | | |  |
| #INFO: | QUERY: | ENSOARG00000009317 | PRKAA1 | protein kinase AMP-activated catalytic subunit alpha 1 [Source:HGNC Symbol;Acc:HGNC:9376] | | | | | | | | | |
| #INFO: | QUERY: | ENSOARG00000009355 | N/A | N/A |  |  |  |  |  |  |  |  |  |
| #INFO: | QUERY: | ENSOARG00000009358 | TTC33 | tetratricopeptide repeat domain 33 [Source:HGNC Symbol;Acc:HGNC:29959] | | | | | | | |  |  |
| #INFO: | QUERY: | ENSOARG00000009385 | NT5DC1 | 5'-nucleotidase domain containing 1 [Source:HGNC Symbol;Acc:HGNC:21556] | | | | | | | |  |  |
| #INFO: | QUERY: | ENSOARG00000009420 | LYPD6B | LY6/PLAUR domain containing 6B [Source:HGNC Symbol;Acc:HGNC:27018] | | | | | | | |  |  |
| #INFO: | QUERY: | ENSOARG00000009451 | HS3ST5 | heparan sulfate-glucosamine 3-sulfotransferase 5 [Source:HGNC Symbol;Acc:HGNC:19419] | | | | | | | | |  |
| #INFO: | QUERY: | ENSOARG00000009502 | N/A | N/A |  |  |  |  |  |  |  |  |  |
| #INFO: | QUERY: | ENSOARG00000009527 | N/A | N/A |  |  |  |  |  |  |  |  |  |
| #INFO: | QUERY: | ENSOARG00000009576 | PLA2G2E | phospholipase A2 group IIE [Source:HGNC Symbol;Acc:HGNC:13414] | | | | | | |  |  |  |
| #INFO: | QUERY: | ENSOARG00000009592 | OTUD3 | OTU deubiquitinase 3 [Source:HGNC Symbol;Acc:HGNC:29038] | | | | | | |  |  |  |
| #INFO: | QUERY: | ENSOARG00000009786 | N/A | N/A |  |  |  |  |  |  |  |  |  |
| #INFO: | QUERY: | ENSOARG00000009801 | ADAL | adenosine deaminase like [Source:HGNC Symbol;Acc:HGNC:31853] | | | | | | |  |  |  |
| #INFO: | QUERY: | ENSOARG00000009802 | PPP2R2A | protein phosphatase 2 regulatory subunit Balpha [Source:HGNC Symbol;Acc:HGNC:9304] | | | | | | | | |  |
| #INFO: | QUERY: | ENSOARG00000009856 | HEXIM2 | hexamethylene bisacetamide inducible 2 [Source:HGNC Symbol;Acc:HGNC:28591] | | | | | | | |  |  |
| #INFO: | QUERY: | ENSOARG00000009877 | SLC16A10 | solute carrier family 16 member 10 [Source:HGNC Symbol;Acc:HGNC:17027] | | | | | | | |  |  |
| #INFO: | QUERY: | ENSOARG00000009883 | ZSCAN29 | zinc finger and SCAN domain containing 29 [Source:HGNC Symbol;Acc:HGNC:26673] | | | | | | | | |  |
| #INFO: | QUERY: | ENSOARG00000009910 | N/A | N/A |  |  |  |  |  |  |  |  |  |
| #INFO: | QUERY: | ENSOARG00000009948 | ZBTB46 | zinc finger and BTB domain containing 46 [Source:HGNC Symbol;Acc:HGNC:16094] | | | | | | | |  |  |
| #INFO: | QUERY: | ENSOARG00000009972 | FMNL1 | formin like 1 [Source:HGNC Symbol;Acc:HGNC:1212] | | | | | |  |  |  |  |
| #INFO: | QUERY: | ENSOARG00000009985 | TUBGCP4 | tubulin gamma complex associated protein 4 [Source:HGNC Symbol;Acc:HGNC:16691] | | | | | | | | |  |
| #INFO: | QUERY: | ENSOARG00000010070 | N/A | N/A |  |  |  |  |  |  |  |  |  |
| #INFO: | QUERY: | ENSOARG00000010127 | CRIM1 | cysteine rich transmembrane BMP regulator 1 [Source:HGNC Symbol;Acc:HGNC:2359] | | | | | | | | |  |
| #INFO: | QUERY: | ENSOARG00000010128 | ZGPAT | zinc finger CCCH-type and G-patch domain containing [Source:HGNC Symbol;Acc:HGNC:15948] | | | | | | | | | |
| #INFO: | QUERY: | ENSOARG00000010142 | TNKS | tankyrase [Source:HGNC Symbol;Acc:HGNC:11941] | | | | |  |  |  |  |  |
| #INFO: | QUERY: | ENSOARG00000010147 | TP53BP1 | tumor protein p53 binding protein 1 [Source:HGNC Symbol;Acc:HGNC:11999] | | | | | | | |  |  |
| #INFO: | QUERY: | ENSOARG00000010188 | ARFRP1 | ADP ribosylation factor related protein 1 [Source:HGNC Symbol;Acc:HGNC:662] | | | | | | | |  |  |
| #INFO: | QUERY: | ENSOARG00000010214 | N/A | N/A |  |  |  |  |  |  |  |  |  |
| #INFO: | QUERY: | ENSOARG00000010247 | ATAD2 | ATPase family, AAA domain containing 2 [Source:HGNC Symbol;Acc:HGNC:30123] | | | | | | | |  |  |
| #INFO: | QUERY: | ENSOARG00000010296 | SPATA32 | spermatogenesis associated 32 [Source:HGNC Symbol;Acc:HGNC:26349] | | | | | | |  |  |  |
| #INFO: | QUERY: | ENSOARG00000010322 | TNFRSF6B | TNF receptor superfamily member 6b [Source:HGNC Symbol;Acc:HGNC:11921] | | | | | | | |  |  |
| #INFO: | QUERY: | ENSOARG00000010344 | LTBP1 | latent transforming growth factor beta binding protein 1 [Source:HGNC Symbol;Acc:HGNC:6714] | | | | | | | | | |
| #INFO: | QUERY: | ENSOARG00000010357 | PIWIL2 | piwi like RNA-mediated gene silencing 2 [Source:HGNC Symbol;Acc:HGNC:17644] | | | | | | | |  |  |
| #INFO: | QUERY: | ENSOARG00000010368 | N/A | N/A |  |  |  |  |  |  |  |  |  |
| #INFO: | QUERY: | ENSOARG00000010372 | N/A | N/A |  |  |  |  |  |  |  |  |  |
| #INFO: | QUERY: | ENSOARG00000010382 | THSD7B | thrombospondin type 1 domain containing 7B [Source:HGNC Symbol;Acc:HGNC:29348] | | | | | | | | |  |
| #INFO: | QUERY: | ENSOARG00000010384 | PHYHIP | phytanoyl-CoA 2-hydroxylase interacting protein [Source:HGNC Symbol;Acc:HGNC:16865] | | | | | | | | |  |
| #INFO: | QUERY: | ENSOARG00000010396 | MAP3K14 | mitogen-activated protein kinase kinase kinase 14 [Source:HGNC Symbol;Acc:HGNC:6853] | | | | | | | | |  |
| #INFO: | QUERY: | ENSOARG00000010424 | FAM19A1 | family with sequence similarity 19 member A1, C-C motif chemokine like [Source:HGNC Symbol;Acc:HGNC:21587] | | | | | | | | | |
| #INFO: | QUERY: | ENSOARG00000010433 | SUCLG2 | succinate-CoA ligase GDP-forming beta subunit [Source:HGNC Symbol;Acc:HGNC:11450] | | | | | | | | |  |
| #INFO: | QUERY: | ENSOARG00000010458 | BATF3 | basic leucine zipper ATF-like transcription factor 3 [Source:HGNC Symbol;Acc:HGNC:28915] | | | | | | | | |  |
| #INFO: | QUERY: | ENSOARG00000010486 | ZHX1 | zinc fingers and homeoboxes 1 [Source:HGNC Symbol;Acc:HGNC:12871] | | | | | | |  |  |  |
| #INFO: | QUERY: | ENSOARG00000010512 | SGCD | sarcoglycan delta [Source:HGNC Symbol;Acc:HGNC:10807] | | | | | |  |  |  |  |
| #INFO: | QUERY: | ENSOARG00000010544 | DARS | aspartyl-tRNA synthetase [Source:HGNC Symbol;Acc:HGNC:2678] | | | | | | |  |  |  |
| #INFO: | QUERY: | ENSOARG00000010554 | N/A | N/A |  |  |  |  |  |  |  |  |  |
| #INFO: | QUERY: | ENSOARG00000010555 | STMN3 | stathmin 3 [Source:HGNC Symbol;Acc:HGNC:15926] | | | | |  |  |  |  |  |
| #INFO: | QUERY: | ENSOARG00000010596 | GMEB2 | glucocorticoid modulatory element binding protein 2 [Source:HGNC Symbol;Acc:HGNC:4371] | | | | | | | | |  |
| #INFO: | QUERY: | ENSOARG00000010621 | N/A | N/A |  |  |  |  |  |  |  |  |  |
| #INFO: | QUERY: | ENSOARG00000010689 | LCT | lactase [Source:HGNC Symbol;Acc:HGNC:6530] | | | | |  |  |  |  |  |
| #INFO: | QUERY: | ENSOARG00000010768 | AFG1L | AFG1 like ATPase [Source:HGNC Symbol;Acc:HGNC:16411] | | | | | |  |  |  |  |
| #INFO: | QUERY: | ENSOARG00000010773 | FAM83A | family with sequence similarity 83 member A [Source:HGNC Symbol;Acc:HGNC:28210] | | | | | | | | |  |
| #INFO: | QUERY: | ENSOARG00000010788 | R3HDM1 | R3H domain containing 1 [Source:HGNC Symbol;Acc:HGNC:9757] | | | | | | |  |  |  |
| #INFO: | QUERY: | ENSOARG00000010832 | TBC1D31 | TBC1 domain family member 31 [Source:HGNC Symbol;Acc:HGNC:30888] | | | | | | |  |  |  |
| #INFO: | QUERY: | ENSOARG00000010843 | DTL | denticleless E3 ubiquitin protein ligase homolog [Source:HGNC Symbol;Acc:HGNC:30288] | | | | | | | | |  |
| #INFO: | QUERY: | ENSOARG00000010989 | SOBP | sine oculis binding protein homolog [Source:HGNC Symbol;Acc:HGNC:29256] | | | | | | | |  |  |
| #INFO: | QUERY: | ENSOARG00000011000 | DNAJC11 | DnaJ heat shock protein family (Hsp40) member C11 [Source:HGNC Symbol;Acc:HGNC:25570] | | | | | | | | |  |
| #INFO: | QUERY: | ENSOARG00000011034 | N/A | N/A |  |  |  |  |  |  |  |  |  |
| #INFO: | QUERY: | ENSOARG00000011053 | HAS2 | hyaluronan synthase 2 [Source:HGNC Symbol;Acc:HGNC:4819] | | | | | |  |  |  |  |
| #INFO: | QUERY: | ENSOARG00000011055 | PADI1 | peptidyl arginine deiminase 1 [Source:HGNC Symbol;Acc:HGNC:18367] | | | | | | |  |  |  |
| #INFO: | QUERY: | ENSOARG00000011173 | IL12RB2 | interleukin-12 receptor subunit beta-2 precursor [Source:RefSeq peptide;Acc:NP_001239104] | | | | | | | | | |
| #INFO: | QUERY: | ENSOARG00000011199 | ATG5 | autophagy related 5 [Source:HGNC Symbol;Acc:HGNC:589] | | | | | |  |  |  |  |
| #INFO: | QUERY: | ENSOARG00000011227 | N/A | N/A |  |  |  |  |  |  |  |  |  |
| #INFO: | QUERY: | ENSOARG00000011230 | THAP3 | THAP domain containing 3 [Source:HGNC Symbol;Acc:HGNC:20855] | | | | | | |  |  |  |
| #INFO: | QUERY: | ENSOARG00000011279 | PHF13 | PHD finger protein 13 [Source:HGNC Symbol;Acc:HGNC:22983] | | | | | | |  |  |  |
| #INFO: | QUERY: | ENSOARG00000011282 | SERBP1 | SERPINE1 mRNA binding protein 1 [Source:HGNC Symbol;Acc:HGNC:17860] | | | | | | | |  |  |
| #INFO: | QUERY: | ENSOARG00000011331 | PREP | prolyl endopeptidase [Source:HGNC Symbol;Acc:HGNC:9358] | | | | | |  |  |  |  |
| #INFO: | QUERY: | ENSOARG00000011354 | KLHL21 | kelch like family member 21 [Source:HGNC Symbol;Acc:HGNC:29041] | | | | | | |  |  |  |
| #INFO: | QUERY: | ENSOARG00000011375 | N/A | N/A |  |  |  |  |  |  |  |  |  |
| #INFO: | QUERY: | ENSOARG00000011416 | ADAM22 | ADAM metallopeptidase domain 22 [Source:HGNC Symbol;Acc:HGNC:201] | | | | | | | |  |  |
| #INFO: | QUERY: | ENSOARG00000011426 | ZBTB48 | zinc finger and BTB domain containing 48 [Source:HGNC Symbol;Acc:HGNC:4930] | | | | | | | |  |  |
| #INFO: | QUERY: | ENSOARG00000011435 | HACE1 | HECT domain and ankyrin repeat containing E3 ubiquitin protein ligase 1 [Source:HGNC Symbol;Acc:HGNC:21033] | | | | | | | | | |
| #INFO: | QUERY: | ENSOARG00000011520 | N/A | N/A |  |  |  |  |  |  |  |  |  |
| #INFO: | QUERY: | ENSOARG00000011552 | DRD1 | dopamine receptor D1 [Source:HGNC Symbol;Acc:HGNC:3020] | | | | | |  |  |  |  |
| #INFO: | QUERY: | ENSOARG00000011614 | N/A | N/A |  |  |  |  |  |  |  |  |  |
| #INFO: | QUERY: | ENSOARG00000011616 | N/A | N/A |  |  |  |  |  |  |  |  |  |
| #INFO: | QUERY: | ENSOARG00000011622 | C1ORF158 | chromosome 1 open reading frame 158 [Source:HGNC Symbol;Acc:HGNC:28567] | | | | | | | |  |  |
| #INFO: | QUERY: | ENSOARG00000011653 | RXFP2 | relaxin/insulin like family peptide receptor 2 [Source:HGNC Symbol;Acc:HGNC:17318] | | | | | | | | |  |
| #INFO: | QUERY: | ENSOARG00000011688 | NOC4L | nucleolar complex associated 4 homolog [Source:HGNC Symbol;Acc:HGNC:28461] | | | | | | | |  |  |
| #INFO: | QUERY: | ENSOARG00000011703 | VPS53 | VPS53, GARP complex subunit [Source:HGNC Symbol;Acc:HGNC:25608] | | | | | | |  |  |  |
| #INFO: | QUERY: | ENSOARG00000011716 | DDB1 | damage specific DNA binding protein 1 [Source:HGNC Symbol;Acc:HGNC:2717] | | | | | | | |  |  |
| #INFO: | QUERY: | ENSOARG00000011723 | AADACL3 | arylacetamide deacetylase like 3 [Source:HGNC Symbol;Acc:HGNC:32037] | | | | | | | |  |  |
| #INFO: | QUERY: | ENSOARG00000011759 | SRI | sorcin [Source:RefSeq peptide;Acc:NP_001157075] | | | | |  |  |  |  |  |
| #INFO: | QUERY: | ENSOARG00000011771 | TESPA1 | thymocyte expressed, positive selection associated 1 [Source:HGNC Symbol;Acc:HGNC:29109] | | | | | | | | | |
| #INFO: | QUERY: | ENSOARG00000011824 | SERPINH1 | serpin peptidase inhibitor, clade H (heat shock protein 47), member 1, (collagen binding protein 1) precursor [Source:RefSeq peptide;Acc:NP_001295522] | | | | | | | | | |
| #INFO: | QUERY: | ENSOARG00000011871 | N/A | N/A |  |  |  |  |  |  |  |  |  |
| #INFO: | QUERY: | ENSOARG00000011875 | MAP6 | microtubule associated protein 6 [Source:HGNC Symbol;Acc:HGNC:6868] | | | | | | | |  |  |
| #INFO: | QUERY: | ENSOARG00000011929 | N/A | N/A |  |  |  |  |  |  |  |  |  |
| #INFO: | QUERY: | ENSOARG00000011968 | RPH3AL | rabphilin 3A like (without C2 domains) [Source:HGNC Symbol;Acc:HGNC:10296] | | | | | | | |  |  |
| #INFO: | QUERY: | ENSOARG00000012030 | N/A | N/A |  |  |  |  |  |  |  |  |  |
| #INFO: | QUERY: | ENSOARG00000012073 | N/A | N/A |  |  |  |  |  |  |  |  |  |
| #INFO: | QUERY: | ENSOARG00000012094 | FHL5 | four and a half LIM domains 5 [Source:HGNC Symbol;Acc:HGNC:17371] | | | | | | |  |  |  |
| #INFO: | QUERY: | ENSOARG00000012124 | N/A | N/A |  |  |  |  |  |  |  |  |  |
| #INFO: | QUERY: | ENSOARG00000012334 | PXK | PX domain containing serine/threonine kinase like [Source:HGNC Symbol;Acc:HGNC:23326] | | | | | | | | |  |
| #INFO: | QUERY: | ENSOARG00000012340 | C1QL2 | complement C1q like 2 [Source:HGNC Symbol;Acc:HGNC:24181] | | | | | | |  |  |  |
| #INFO: | QUERY: | ENSOARG00000012424 | N/A | N/A |  |  |  |  |  |  |  |  |  |
| #INFO: | QUERY: | ENSOARG00000012448 | ZNF469 | zinc finger protein 469 [Source:HGNC Symbol;Acc:HGNC:23216] | | | | | | |  |  |  |
| #INFO: | QUERY: | ENSOARG00000012475 | ABHD6 | abhydrolase domain containing 6 [Source:HGNC Symbol;Acc:HGNC:21398] | | | | | | | |  |  |
| #INFO: | QUERY: | ENSOARG00000012485 | VWCE | von Willebrand factor C and EGF domains [Source:HGNC Symbol;Acc:HGNC:26487] | | | | | | | |  |  |
| #INFO: | QUERY: | ENSOARG00000012564 | ERC1 | ELKS/RAB6-interacting/CAST family member 1 [Source:HGNC Symbol;Acc:HGNC:17072] | | | | | | | | |  |
| #INFO: | QUERY: | ENSOARG00000012659 | RORB | nuclear receptor ROR-beta [Source:RefSeq peptide;Acc:NP_001123209] | | | | | | |  |  |  |
| #INFO: | QUERY: | ENSOARG00000012675 | TKFC | triokinase and FMN cyclase [Source:HGNC Symbol;Acc:HGNC:24552] | | | | | | |  |  |  |
| #INFO: | QUERY: | ENSOARG00000012693 | GPR153 | G protein-coupled receptor 153 [Source:HGNC Symbol;Acc:HGNC:23618] | | | | | | |  |  |  |
| #INFO: | QUERY: | ENSOARG00000012739 | TANC2 | tetratricopeptide repeat, ankyrin repeat and coiled-coil containing 2 [Source:HGNC Symbol;Acc:HGNC:30212] | | | | | | | | | |
| #INFO: | QUERY: | ENSOARG00000012812 | CYB561A3 | cytochrome b561 family member A3 [Source:HGNC Symbol;Acc:HGNC:23014] | | | | | | | |  |  |
| #INFO: | QUERY: | ENSOARG00000012843 | TMEM138 | transmembrane protein 138 [Source:HGNC Symbol;Acc:HGNC:26944] | | | | | | |  |  |  |
| #INFO: | QUERY: | ENSOARG00000012873 | TMEM216 | transmembrane protein 216 [Source:HGNC Symbol;Acc:HGNC:25018] | | | | | | |  |  |  |
| #INFO: | QUERY: | ENSOARG00000012888 | EPB41L5 | erythrocyte membrane protein band 4.1 like 5 [Source:HGNC Symbol;Acc:HGNC:19819] | | | | | | | | |  |
| #INFO: | QUERY: | ENSOARG00000012910 | RNF207 | ring finger protein 207 [Source:HGNC Symbol;Acc:HGNC:32947] | | | | | | |  |  |  |
| #INFO: | QUERY: | ENSOARG00000012937 | CPSF7 | cleavage and polyadenylation specific factor 7 [Source:HGNC Symbol;Acc:HGNC:30098] | | | | | | | | |  |
| #INFO: | QUERY: | ENSOARG00000013014 | SLC9A8 | solute carrier family 9 member A8 [Source:HGNC Symbol;Acc:HGNC:20728] | | | | | | | |  |  |
| #INFO: | QUERY: | ENSOARG00000013037 | N/A | N/A |  |  |  |  |  |  |  |  |  |
| #INFO: | QUERY: | ENSOARG00000013041 | N/A | N/A |  |  |  |  |  |  |  |  |  |
| #INFO: | QUERY: | ENSOARG00000013051 | N/A | N/A |  |  |  |  |  |  |  |  |  |
| #INFO: | QUERY: | ENSOARG00000013093 | TMEM252 | transmembrane protein 252 [Source:HGNC Symbol;Acc:HGNC:28537] | | | | | | |  |  |  |
| #INFO: | QUERY: | ENSOARG00000013106 | N/A | N/A |  |  |  |  |  |  |  |  |  |
| #INFO: | QUERY: | ENSOARG00000013159 | IL1R2 | interleukin 1 receptor type 2 [Source:HGNC Symbol;Acc:HGNC:5994] | | | | | | |  |  |  |
| #INFO: | QUERY: | ENSOARG00000013332 | LONRF2 | LON peptidase N-terminal domain and ring finger 2 [Source:HGNC Symbol;Acc:HGNC:24788] | | | | | | | | |  |
| #INFO: | QUERY: | ENSOARG00000013378 | GLIS3 | GLIS family zinc finger 3 [Source:HGNC Symbol;Acc:HGNC:28510] | | | | | | |  |  |  |
| #INFO: | QUERY: | ENSOARG00000013425 | CHD5 | chromodomain helicase DNA binding protein 5 [Source:HGNC Symbol;Acc:HGNC:16816] | | | | | | | | |  |
| #INFO: | QUERY: | ENSOARG00000013463 | RCL1 | RNA terminal phosphate cyclase like 1 [Source:HGNC Symbol;Acc:HGNC:17687] | | | | | | | |  |  |
| #INFO: | QUERY: | ENSOARG00000013480 | JAK2 | Janus kinase 2 [Source:HGNC Symbol;Acc:HGNC:6192] | | | | | |  |  |  |  |
| #INFO: | QUERY: | ENSOARG00000013492 | INSL6 | insulin like 6 [Source:HGNC Symbol;Acc:HGNC:6089] | | | | | |  |  |  |  |
| #INFO: | QUERY: | ENSOARG00000013500 | PLGRKT | plasminogen receptor with a C-terminal lysine [Source:HGNC Symbol;Acc:HGNC:23633] | | | | | | | | |  |
| #INFO: | QUERY: | ENSOARG00000013507 | ZCCHC10 | zinc finger CCHC-type containing 10 [Source:HGNC Symbol;Acc:HGNC:25954] | | | | | | | |  |  |
| #INFO: | QUERY: | ENSOARG00000013512 | CDT1 | chromatin licensing and DNA replication factor 1 [Source:HGNC Symbol;Acc:HGNC:24576] | | | | | | | | |  |
| #INFO: | QUERY: | ENSOARG00000013556 | APRT | adenine phosphoribosyltransferase [Source:HGNC Symbol;Acc:HGNC:626] | | | | | | | |  |  |
| #INFO: | QUERY: | ENSOARG00000013587 | GALNS | galactosamine (N-acetyl)-6-sulfatase [Source:HGNC Symbol;Acc:HGNC:4122] | | | | | | | |  |  |
| #INFO: | QUERY: | ENSOARG00000013588 | MED25 | mediator complex subunit 25 [Source:HGNC Symbol;Acc:HGNC:28845] | | | | | | |  |  |  |
| #INFO: | QUERY: | ENSOARG00000013626 | TMEM131 | transmembrane protein 131 [Source:HGNC Symbol;Acc:HGNC:30366] | | | | | | |  |  |  |
| #INFO: | QUERY: | ENSOARG00000013640 | TRAPPC2L | trafficking protein particle complex 2 like [Source:HGNC Symbol;Acc:HGNC:30887] | | | | | | | |  |  |
| #INFO: | QUERY: | ENSOARG00000013644 | PTOV1 | prostate tumor overexpressed 1 [Source:HGNC Symbol;Acc:HGNC:9632] | | | | | | |  |  |  |
| #INFO: | QUERY: | ENSOARG00000013653 | N/A | N/A |  |  |  |  |  |  |  |  |  |
| #INFO: | QUERY: | ENSOARG00000013656 | AKT1S1 | AKT1 substrate 1 [Source:HGNC Symbol;Acc:HGNC:28426] | | | | | |  |  |  |  |
| #INFO: | QUERY: | ENSOARG00000013667 | TBC1D17 | TBC1 domain family member 17 [Source:HGNC Symbol;Acc:HGNC:25699] | | | | | | |  |  |  |
| #INFO: | QUERY: | ENSOARG00000013677 | IL4I1 | interleukin 4 induced 1 [Source:HGNC Symbol;Acc:HGNC:19094] | | | | | | |  |  |  |
| #INFO: | QUERY: | ENSOARG00000013678 | RAMP3 | receptor activity modifying protein 3 [Source:HGNC Symbol;Acc:HGNC:9845] | | | | | | | |  |  |
| #INFO: | QUERY: | ENSOARG00000013682 | PABPN1L | poly(A) binding protein nuclear 1 like, cytoplasmic [Source:HGNC Symbol;Acc:HGNC:37237] | | | | | | | | |  |
| #INFO: | QUERY: | ENSOARG00000013686 | NUP62 | nucleoporin 62 [Source:HGNC Symbol;Acc:HGNC:8066] | | | | | |  |  |  |  |
| #INFO: | QUERY: | ENSOARG00000013723 | CBFA2T3 | CBFA2/RUNX1 translocation partner 3 [Source:HGNC Symbol;Acc:HGNC:1537] | | | | | | | |  |  |
| #INFO: | QUERY: | ENSOARG00000013742 | LHX2 | LIM homeobox 2 [Source:HGNC Symbol;Acc:HGNC:6594] | | | | | |  |  |  |  |
| #INFO: | QUERY: | ENSOARG00000013753 | EGFR | epidermal growth factor receptor [Source:HGNC Symbol;Acc:HGNC:3236] | | | | | | | |  |  |
| #INFO: | QUERY: | ENSOARG00000013754 | N/A | N/A |  |  |  |  |  |  |  |  |  |
| #INFO: | QUERY: | ENSOARG00000013794 | N/A | N/A |  |  |  |  |  |  |  |  |  |
| #INFO: | QUERY: | ENSOARG00000013862 | TLCD2 | TLC domain containing 2 [Source:HGNC Symbol;Acc:HGNC:33522] | | | | | | |  |  |  |
| #INFO: | QUERY: | ENSOARG00000013919 | N/A | N/A |  |  |  |  |  |  |  |  |  |
| #INFO: | QUERY: | ENSOARG00000013920 | WDR81 | WD repeat domain 81 [Source:HGNC Symbol;Acc:HGNC:26600] | | | | | | |  |  |  |
| #INFO: | QUERY: | ENSOARG00000013922 | TAF3 | TATA-box binding protein associated factor 3 [Source:HGNC Symbol;Acc:HGNC:17303] | | | | | | | | |  |
| #INFO: | QUERY: | ENSOARG00000013960 | USP4 | ubiquitin specific peptidase 4 [Source:HGNC Symbol;Acc:HGNC:12627] | | | | | | |  |  |  |
| #INFO: | QUERY: | ENSOARG00000013970 | RAB11FIP4 | RAB11 family interacting protein 4 [Source:HGNC Symbol;Acc:HGNC:30267] | | | | | | | |  |  |
| #INFO: | QUERY: | ENSOARG00000013971 | ZEB1 | zinc finger E-box binding homeobox 1 [Source:HGNC Symbol;Acc:HGNC:11642] | | | | | | | |  |  |
| #INFO: | QUERY: | ENSOARG00000014059 | BNC2 | basonuclin 2 [Source:HGNC Symbol;Acc:HGNC:30988] | | | | | |  |  |  |  |
| #INFO: | QUERY: | ENSOARG00000014081 | SERPINF2 | serpin family F member 2 [Source:HGNC Symbol;Acc:HGNC:9075] | | | | | | |  |  |  |
| #INFO: | QUERY: | ENSOARG00000014111 | SEC24A | SEC24 homolog A, COPII coat complex component [Source:HGNC Symbol;Acc:HGNC:10703] | | | | | | | | |  |
| #INFO: | QUERY: | ENSOARG00000014133 | ADAMTSL1 | ADAMTS like 1 [Source:HGNC Symbol;Acc:HGNC:14632] | | | | | |  |  |  |  |
| #INFO: | QUERY: | ENSOARG00000014135 | HTD2 | hydroxyacyl-thioester dehydratase type 2 [Source:HGNC Symbol;Acc:HGNC:53111] | | | | | | | |  |  |
| #INFO: | QUERY: | ENSOARG00000014174 | CCM2 | CCM2 scaffolding protein [Source:HGNC Symbol;Acc:HGNC:21708] | | | | | | |  |  |  |
| #INFO: | QUERY: | ENSOARG00000014215 | C3ORF62 | chromosome 3 open reading frame 62 [Source:HGNC Symbol;Acc:HGNC:24771] | | | | | | | |  |  |
| #INFO: | QUERY: | ENSOARG00000014216 | MYO1G | myosin IG [Source:HGNC Symbol;Acc:HGNC:13880] | | | | |  |  |  |  |  |
| #INFO: | QUERY: | ENSOARG00000014231 | SAR1B | secretion associated Ras related GTPase 1B [Source:HGNC Symbol;Acc:HGNC:10535] | | | | | | | | |  |
| #INFO: | QUERY: | ENSOARG00000014232 | SERPINF1 | serpin family F member 1 [Source:HGNC Symbol;Acc:HGNC:8824] | | | | | | |  |  |  |
| #INFO: | QUERY: | ENSOARG00000014273 | CCDC36 | coiled-coil domain containing 36 [Source:HGNC Symbol;Acc:HGNC:27945] | | | | | | | |  |  |
| #INFO: | QUERY: | ENSOARG00000014296 | KLHDC8B | kelch domain containing 8B [Source:HGNC Symbol;Acc:HGNC:28557] | | | | | | |  |  |  |
| #INFO: | QUERY: | ENSOARG00000014306 | CAMLG | calcium modulating ligand [Source:HGNC Symbol;Acc:HGNC:1471] | | | | | | |  |  |  |
| #INFO: | QUERY: | ENSOARG00000014348 | DDX46 | DEAD-box helicase 46 [Source:HGNC Symbol;Acc:HGNC:18681] | | | | | | |  |  |  |
| #INFO: | QUERY: | ENSOARG00000014388 | CCDC71 | coiled-coil domain containing 71 [Source:HGNC Symbol;Acc:HGNC:25760] | | | | | | | |  |  |
| #INFO: | QUERY: | ENSOARG00000014452 | EFL1 | elongation factor like GTPase 1 [Source:HGNC Symbol;Acc:HGNC:25789] | | | | | | |  |  |  |
| #INFO: | QUERY: | ENSOARG00000014461 | COL24A1 | collagen type XXIV alpha 1 chain [Source:HGNC Symbol;Acc:HGNC:20821] | | | | | | | |  |  |
| #INFO: | QUERY: | ENSOARG00000014551 | SH2B2 | SH2B adaptor protein 2 [Source:HGNC Symbol;Acc:HGNC:17381] | | | | | | |  |  |  |
| #INFO: | QUERY: | ENSOARG00000014559 | LAMB2 | laminin subunit beta 2 [Source:HGNC Symbol;Acc:HGNC:6487] | | | | | |  |  |  |  |
| #INFO: | QUERY: | ENSOARG00000014594 | N/A | N/A |  |  |  |  |  |  |  |  |  |
| #INFO: | QUERY: | ENSOARG00000014629 | CUX1 | cut like homeobox 1 [Source:HGNC Symbol;Acc:HGNC:2557] | | | | | |  |  |  |  |
| #INFO: | QUERY: | ENSOARG00000014786 | EIF3E | eukaryotic translation initiation factor 3 subunit E [Source:HGNC Symbol;Acc:HGNC:3277] | | | | | | | | |  |
| #INFO: | QUERY: | ENSOARG00000014803 | N/A | N/A |  |  |  |  |  |  |  |  |  |
| #INFO: | QUERY: | ENSOARG00000014867 | MSH3 | mutS homolog 3 [Source:HGNC Symbol;Acc:HGNC:7326] | | | | | |  |  |  |  |
| #INFO: | QUERY: | ENSOARG00000014927 | USP19 | ubiquitin specific peptidase 19 [Source:HGNC Symbol;Acc:HGNC:12617] | | | | | | |  |  |  |
| #INFO: | QUERY: | ENSOARG00000015005 | N/A | N/A |  |  |  |  |  |  |  |  |  |
| #INFO: | QUERY: | ENSOARG00000015066 | RASGRF2 | Ras protein specific guanine nucleotide releasing factor 2 [Source:HGNC Symbol;Acc:HGNC:9876] | | | | | | | | | |
| #INFO: | QUERY: | ENSOARG00000015151 | ZFAND3 | zinc finger AN1-type containing 3 [Source:HGNC Symbol;Acc:HGNC:18019] | | | | | | | |  |  |
| #INFO: | QUERY: | ENSOARG00000015175 | MTMR9 | myotubularin related protein 9 [Source:HGNC Symbol;Acc:HGNC:14596] | | | | | | |  |  |  |
| #INFO: | QUERY: | ENSOARG00000015180 | BAMBI | BMP and activin membrane-bound inhibitor homolog precursor [Source:RefSeq peptide;Acc:NP_001009761] | | | | | | | | | |
| #INFO: | QUERY: | ENSOARG00000015190 | N/A | N/A |  |  |  |  |  |  |  |  |  |
| #INFO: | QUERY: | ENSOARG00000015218 | N/A | N/A |  |  |  |  |  |  |  |  |  |
| #INFO: | QUERY: | ENSOARG00000015249 | CDH26 | cadherin 26 [Source:HGNC Symbol;Acc:HGNC:15902] | | | | | |  |  |  |  |
| #INFO: | QUERY: | ENSOARG00000015363 | QRICH1 | glutamine rich 1 [Source:HGNC Symbol;Acc:HGNC:24713] | | | | | |  |  |  |  |
| #INFO: | QUERY: | ENSOARG00000015447 | N/A | N/A |  |  |  |  |  |  |  |  |  |
| #INFO: | QUERY: | ENSOARG00000015458 | SYCP2 | synaptonemal complex protein 2 [Source:HGNC Symbol;Acc:HGNC:11490] | | | | | | | |  |  |
| #INFO: | QUERY: | ENSOARG00000015616 | ABCA13 | ATP binding cassette subfamily A member 13 [Source:HGNC Symbol;Acc:HGNC:14638] | | | | | | | | |  |
| #INFO: | QUERY: | ENSOARG00000015657 | NDUFAF3 | NADH:ubiquinone oxidoreductase complex assembly factor 3 [Source:HGNC Symbol;Acc:HGNC:29918] | | | | | | | | | |
| #INFO: | QUERY: | ENSOARG00000015667 | N/A | N/A |  |  |  |  |  |  |  |  |  |
| #INFO: | QUERY: | ENSOARG00000015672 | DALRD3 | DALR anticodon binding domain containing 3 [Source:HGNC Symbol;Acc:HGNC:25536] | | | | | | | | |  |
| #INFO: | QUERY: | ENSOARG00000015694 | N/A | N/A |  |  |  |  |  |  |  |  |  |
| #INFO: | QUERY: | ENSOARG00000015699 | TRBV2 | T-cell receptor beta variable 2 [Source:HGNC Symbol;Acc:HGNC:12195] | | | | | | |  |  |  |
| #INFO: | QUERY: | ENSOARG00000015724 | EDIL3 | EGF like repeats and discoidin domains 3 [Source:HGNC Symbol;Acc:HGNC:3173] | | | | | | | |  |  |
| #INFO: | QUERY: | ENSOARG00000015725 | CAMK2B | calcium/calmodulin dependent protein kinase II beta [Source:HGNC Symbol;Acc:HGNC:1461] | | | | | | | | |  |
| #INFO: | QUERY: | ENSOARG00000015742 | IL16 | interleukin 16 [Source:HGNC Symbol;Acc:HGNC:5980] | | | | | |  |  |  |  |
| #INFO: | QUERY: | ENSOARG00000015769 | N/A | N/A |  |  |  |  |  |  |  |  |  |
| #INFO: | QUERY: | ENSOARG00000015774 | DIP2C | disco interacting protein 2 homolog C [Source:HGNC Symbol;Acc:HGNC:29150] | | | | | | | |  |  |
| #INFO: | QUERY: | ENSOARG00000015797 | N/A | N/A |  |  |  |  |  |  |  |  |  |
| #INFO: | QUERY: | ENSOARG00000015805 | WDR6 | WD repeat domain 6 [Source:HGNC Symbol;Acc:HGNC:12758] | | | | | |  |  |  |  |
| #INFO: | QUERY: | ENSOARG00000015819 | LETM1 | leucine zipper and EF-hand containing transmembrane protein 1 [Source:HGNC Symbol;Acc:HGNC:6556] | | | | | | | | | |
| #INFO: | QUERY: | ENSOARG00000015842 | P4HTM | prolyl 4-hydroxylase, transmembrane [Source:HGNC Symbol;Acc:HGNC:28858] | | | | | | | |  |  |
| #INFO: | QUERY: | ENSOARG00000015843 | PHACTR3 | phosphatase and actin regulator 3 [Source:HGNC Symbol;Acc:HGNC:15833] | | | | | | | |  |  |
| #INFO: | QUERY: | ENSOARG00000015845 | N/A | N/A |  |  |  |  |  |  |  |  |  |
| #INFO: | QUERY: | ENSOARG00000015849 | MRPL42 | mitochondrial ribosomal protein L42 [Source:HGNC Symbol;Acc:HGNC:14493] | | | | | | | |  |  |
| #INFO: | QUERY: | ENSOARG00000015854 | N/A | N/A |  |  |  |  |  |  |  |  |  |
| #INFO: | QUERY: | ENSOARG00000015857 | METRNL | meteorin like, glial cell differentiation regulator [Source:HGNC Symbol;Acc:HGNC:27584] | | | | | | | | |  |
| #INFO: | QUERY: | ENSOARG00000015864 | SOCS2 | suppressor of cytokine signaling 2 [Source:HGNC Symbol;Acc:HGNC:19382] | | | | | | | |  |  |
| #INFO: | QUERY: | ENSOARG00000015868 | N/A | N/A |  |  |  |  |  |  |  |  |  |
| #INFO: | QUERY: | ENSOARG00000015871 | CRADD | CASP2 and RIPK1 domain containing adaptor with death domain [Source:HGNC Symbol;Acc:HGNC:2340] | | | | | | | | | |
| #INFO: | QUERY: | ENSOARG00000015880 | N/A | N/A |  |  |  |  |  |  |  |  |  |
| #INFO: | QUERY: | ENSOARG00000015885 | TOX | thymocyte selection associated high mobility group box [Source:HGNC Symbol;Acc:HGNC:18988] | | | | | | | | | |
| #INFO: | QUERY: | ENSOARG00000015904 | FGFR3 | fibroblast growth factor receptor 3 [Source:HGNC Symbol;Acc:HGNC:3690] | | | | | | | |  |  |
| #INFO: | QUERY: | ENSOARG00000015906 | N/A | N/A |  |  |  |  |  |  |  |  |  |
| #INFO: | QUERY: | ENSOARG00000015913 | N/A | N/A |  |  |  |  |  |  |  |  |  |
| #INFO: | QUERY: | ENSOARG00000015984 | ARIH2 | ariadne RBR E3 ubiquitin protein ligase 2 [Source:HGNC Symbol;Acc:HGNC:690] | | | | | | | |  |  |
| #INFO: | QUERY: | ENSOARG00000016013 | TMEM161B | transmembrane protein 161B [Source:HGNC Symbol;Acc:HGNC:28483] | | | | | | |  |  |  |
| #INFO: | QUERY: | ENSOARG00000016046 | RAB2A | RAB2A, member RAS oncogene family [Source:HGNC Symbol;Acc:HGNC:9763] | | | | | | | |  |  |
| #INFO: | QUERY: | ENSOARG00000016066 | N/A | N/A |  |  |  |  |  |  |  |  |  |
| #INFO: | QUERY: | ENSOARG00000016097 | IWS1 | IWS1, SUPT6H interacting protein [Source:HGNC Symbol;Acc:HGNC:25467] | | | | | | | |  |  |
| #INFO: | QUERY: | ENSOARG00000016130 | APC | APC, WNT signaling pathway regulator [Source:HGNC Symbol;Acc:HGNC:583] | | | | | | | |  |  |
| #INFO: | QUERY: | ENSOARG00000016240 | HNRNPA1 | heterogeneous nuclear ribonucleoprotein A1 [Source:HGNC Symbol;Acc:HGNC:5031] | | | | | | | | |  |
| #INFO: | QUERY: | ENSOARG00000016251 | CBX5 | chromobox 5 [Source:HGNC Symbol;Acc:HGNC:1555] | | | | | |  |  |  |  |
| #INFO: | QUERY: | ENSOARG00000016261 | N/A | N/A |  |  |  |  |  |  |  |  |  |
| #INFO: | QUERY: | ENSOARG00000016267 | OXER1 | oxoeicosanoid receptor 1 [Source:HGNC Symbol;Acc:HGNC:24884] | | | | | | |  |  |  |
| #INFO: | QUERY: | ENSOARG00000016282 | DGKQ | diacylglycerol kinase theta [Source:HGNC Symbol;Acc:HGNC:2856] | | | | | | |  |  |  |
| #INFO: | QUERY: | ENSOARG00000016360 | GAK | cyclin G associated kinase [Source:HGNC Symbol;Acc:HGNC:4113] | | | | | | |  |  |  |
| #INFO: | QUERY: | ENSOARG00000016372 | ART3 | ADP-ribosyltransferase 3 [Source:HGNC Symbol;Acc:HGNC:725] | | | | | | |  |  |  |
| #INFO: | QUERY: | ENSOARG00000016611 | CXCL10 | C-X-C motif chemokine ligand 10 [Source:HGNC Symbol;Acc:HGNC:10637] | | | | | | | |  |  |
| #INFO: | QUERY: | ENSOARG00000016668 | CXCL11 | C-X-C motif chemokine ligand 11 [Source:HGNC Symbol;Acc:HGNC:10638] | | | | | | | |  |  |
| #INFO: | QUERY: | ENSOARG00000016671 | CPEB3 | cytoplasmic polyadenylation element binding protein 3 [Source:HGNC Symbol;Acc:HGNC:21746] | | | | | | | | | |
| #INFO: | QUERY: | ENSOARG00000016672 | ATXN7L3B | ataxin 7 like 3B [Source:HGNC Symbol;Acc:HGNC:37931] | | | | | |  |  |  |  |
| #INFO: | QUERY: | ENSOARG00000016708 | NKAIN3 | sodium/potassium transporting ATPase interacting 3 [Source:HGNC Symbol;Acc:HGNC:26829] | | | | | | | | |  |
| #INFO: | QUERY: | ENSOARG00000016755 | N/A | N/A |  |  |  |  |  |  |  |  |  |
| #INFO: | QUERY: | ENSOARG00000016793 | GGH | gamma-glutamyl hydrolase [Source:HGNC Symbol;Acc:HGNC:4248] | | | | | | |  |  |  |
| #INFO: | QUERY: | ENSOARG00000016795 | OR2C3 | olfactory receptor family 2 subfamily C member 3 [Source:HGNC Symbol;Acc:HGNC:15005] | | | | | | | | |  |
| #INFO: | QUERY: | ENSOARG00000016832 | ATP6V1C1 | ATPase H+ transporting V1 subunit C1 [Source:HGNC Symbol;Acc:HGNC:856] | | | | | | | |  |  |
| #INFO: | QUERY: | ENSOARG00000016843 | TTPA | alpha tocopherol transfer protein [Source:HGNC Symbol;Acc:HGNC:12404] | | | | | | | |  |  |
| #INFO: | QUERY: | ENSOARG00000016854 | CREM | cAMP responsive element modulator [Source:HGNC Symbol;Acc:HGNC:2352] | | | | | | | |  |  |
| #INFO: | QUERY: | ENSOARG00000016882 | YTHDF3 | YTH N6-methyladenosine RNA binding protein 3 [Source:HGNC Symbol;Acc:HGNC:26465] | | | | | | | | |  |
| #INFO: | QUERY: | ENSOARG00000016890 | N/A | N/A |  |  |  |  |  |  |  |  |  |
| #INFO: | QUERY: | ENSOARG00000017029 | CUL2 | cullin 2 [Source:HGNC Symbol;Acc:HGNC:2552] | | | | |  |  |  |  |  |
| #INFO: | QUERY: | ENSOARG00000017077 | ZNF654 | zinc finger protein 654 [Source:HGNC Symbol;Acc:HGNC:25612] | | | | | | |  |  |  |
| #INFO: | QUERY: | ENSOARG00000017138 | C3ORF38 | chromosome 3 open reading frame 38 [Source:HGNC Symbol;Acc:HGNC:28384] | | | | | | | |  |  |
| #INFO: | QUERY: | ENSOARG00000017143 | VAPB | VAMP associated protein B and C [Source:HGNC Symbol;Acc:HGNC:12649] | | | | | | | |  |  |
| #INFO: | QUERY: | ENSOARG00000017222 | SPATA5 | spermatogenesis associated 5 [Source:HGNC Symbol;Acc:HGNC:18119] | | | | | | |  |  |  |
| #INFO: | QUERY: | ENSOARG00000017268 | N/A | N/A |  |  |  |  |  |  |  |  |  |
| #INFO: | QUERY: | ENSOARG00000017275 | RFESD | Rieske Fe-S domain containing [Source:HGNC Symbol;Acc:HGNC:29587] | | | | | | |  |  |  |
| #INFO: | QUERY: | ENSOARG00000017304 | SPATA9 | spermatogenesis associated 9 [Source:HGNC Symbol;Acc:HGNC:22988] | | | | | | |  |  |  |
| #INFO: | QUERY: | ENSOARG00000017392 | TTC19 | tetratricopeptide repeat domain 19 [Source:HGNC Symbol;Acc:HGNC:26006] | | | | | | | |  |  |
| #INFO: | QUERY: | ENSOARG00000017433 | ZSWIM7 | zinc finger SWIM-type containing 7 [Source:HGNC Symbol;Acc:HGNC:26993] | | | | | | | |  |  |
| #INFO: | QUERY: | ENSOARG00000017455 | NUDT6 | nudix hydrolase 6 [Source:HGNC Symbol;Acc:HGNC:8053] | | | | | |  |  |  |  |
| #INFO: | QUERY: | ENSOARG00000017486 | ADORA2B | adenosine A2b receptor [Source:HGNC Symbol;Acc:HGNC:264] | | | | | | |  |  |  |
| #INFO: | QUERY: | ENSOARG00000017548 | KIAA2012 | KIAA2012 [Source:HGNC Symbol;Acc:HGNC:51250] | | | | |  |  |  |  |  |
| #INFO: | QUERY: | ENSOARG00000017553 | N/A | N/A |  |  |  |  |  |  |  |  |  |
| #INFO: | QUERY: | ENSOARG00000017557 | SPECC1 | sperm antigen with calponin homology and coiled-coil domains 1 [Source:HGNC Symbol;Acc:HGNC:30615] | | | | | | | | | |
| #INFO: | QUERY: | ENSOARG00000017563 | N/A | N/A |  |  |  |  |  |  |  |  |  |
| #INFO: | QUERY: | ENSOARG00000017564 | N/A | N/A |  |  |  |  |  |  |  |  |  |
| #INFO: | QUERY: | ENSOARG00000017670 | PLPPR4 | phospholipid phosphatase related 4 [Source:HGNC Symbol;Acc:HGNC:23496] | | | | | | | |  |  |
| #INFO: | QUERY: | ENSOARG00000017726 | RIC8B | RIC8 guanine nucleotide exchange factor B [Source:HGNC Symbol;Acc:HGNC:25555] | | | | | | | | |  |
| #INFO: | QUERY: | ENSOARG00000017750 | NCBP3 | nuclear cap binding subunit 3 [Source:HGNC Symbol;Acc:HGNC:24612] | | | | | | |  |  |  |
| #INFO: | QUERY: | ENSOARG00000017826 | CAMKK1 | calcium/calmodulin dependent protein kinase kinase 1 [Source:HGNC Symbol;Acc:HGNC:1469] | | | | | | | | | |
| #INFO: | QUERY: | ENSOARG00000017828 | N/A | N/A |  |  |  |  |  |  |  |  |  |
| #INFO: | QUERY: | ENSOARG00000017839 | LIMA1 | LIM domain and actin binding 1 [Source:HGNC Symbol;Acc:HGNC:24636] | | | | | | |  |  |  |
| #INFO: | QUERY: | ENSOARG00000017850 | DRD2 | dopamine receptor D2 [Source:HGNC Symbol;Acc:HGNC:3023] | | | | | |  |  |  |  |
| #INFO: | QUERY: | ENSOARG00000017934 | N/A | N/A |  |  |  |  |  |  |  |  |  |
| #INFO: | QUERY: | ENSOARG00000017942 | CHGB | chromogranin B [Source:HGNC Symbol;Acc:HGNC:1930] | | | | | |  |  |  |  |
| #INFO: | QUERY: | ENSOARG00000017981 | N/A | N/A |  |  |  |  |  |  |  |  |  |
| #INFO: | QUERY: | ENSOARG00000017993 | TRMT6 | tRNA methyltransferase 6 [Source:HGNC Symbol;Acc:HGNC:20900] | | | | | | |  |  |  |
| #INFO: | QUERY: | ENSOARG00000018015 | TANGO2 | transport and golgi organization 2 homolog [Source:HGNC Symbol;Acc:HGNC:25439] | | | | | | | | |  |
| #INFO: | QUERY: | ENSOARG00000018040 | NCOA1 | nuclear receptor coactivator 1 [Source:HGNC Symbol;Acc:HGNC:7668] | | | | | | |  |  |  |
| #INFO: | QUERY: | ENSOARG00000018063 | DGCR8 | DGCR8, microprocessor complex subunit [Source:HGNC Symbol;Acc:HGNC:2847] | | | | | | | |  |  |
| #INFO: | QUERY: | ENSOARG00000018145 | MCM8 | minichromosome maintenance 8 homologous recombination repair factor [Source:HGNC Symbol;Acc:HGNC:16147] | | | | | | | | | |
| #INFO: | QUERY: | ENSOARG00000018168 | RREB1 | ras responsive element binding protein 1 [Source:HGNC Symbol;Acc:HGNC:10449] | | | | | | | |  |  |
| #INFO: | QUERY: | ENSOARG00000018195 | TRMT2A | tRNA methyltransferase 2 homolog A [Source:HGNC Symbol;Acc:HGNC:24974] | | | | | | | |  |  |
| #INFO: | QUERY: | ENSOARG00000018318 | RANBP1 | RAN binding protein 1 [Source:HGNC Symbol;Acc:HGNC:9847] | | | | | |  |  |  |  |
| #INFO: | QUERY: | ENSOARG00000018342 | CCSER1 | coiled-coil serine rich protein 1 [Source:HGNC Symbol;Acc:HGNC:29349] | | | | | | |  |  |  |
| #INFO: | QUERY: | ENSOARG00000018357 | CRLS1 | cardiolipin synthase 1 [Source:HGNC Symbol;Acc:HGNC:16148] | | | | | | |  |  |  |
| #INFO: | QUERY: | ENSOARG00000018361 | N/A | N/A |  |  |  |  |  |  |  |  |  |
| #INFO: | QUERY: | ENSOARG00000018440 | LRRN4 | leucine rich repeat neuronal 4 [Source:HGNC Symbol;Acc:HGNC:16208] | | | | | | |  |  |  |
| #INFO: | QUERY: | ENSOARG00000018446 | PTPRZ1 | protein tyrosine phosphatase, receptor type Z1 [Source:HGNC Symbol;Acc:HGNC:9685] | | | | | | | | |  |
| #INFO: | QUERY: | ENSOARG00000018703 | N/A | N/A |  |  |  |  |  |  |  |  |  |
| #INFO: | QUERY: | ENSOARG00000018724 | CUL1 | cullin 1 [Source:HGNC Symbol;Acc:HGNC:2551] | | | | |  |  |  |  |  |
| #INFO: | QUERY: | ENSOARG00000018760 | MYH9 | myosin heavy chain 9 [Source:HGNC Symbol;Acc:HGNC:7579] | | | | | |  |  |  |  |
| #INFO: | QUERY: | ENSOARG00000018841 | N/A | N/A |  |  |  |  |  |  |  |  |  |
| #INFO: | QUERY: | ENSOARG00000018856 | EZH2 | enhancer of zeste 2 polycomb repressive complex 2 subunit [Source:HGNC Symbol;Acc:HGNC:3527] | | | | | | | | | |
| #INFO: | QUERY: | ENSOARG00000018884 | N/A | N/A |  |  |  |  |  |  |  |  |  |
| #INFO: | QUERY: | ENSOARG00000018920 | LMBR1L | limb development membrane protein 1 like [Source:HGNC Symbol;Acc:HGNC:18268] | | | | | | | | |  |
| #INFO: | QUERY: | ENSOARG00000018925 | RHEBL1 | Ras homolog enriched in brain like 1 [Source:HGNC Symbol;Acc:HGNC:21166] | | | | | | | |  |  |
| #INFO: | QUERY: | ENSOARG00000018968 | WDR36 | WD repeat domain 36 [Source:HGNC Symbol;Acc:HGNC:30696] | | | | | | |  |  |  |
| #INFO: | QUERY: | ENSOARG00000019124 | N/A | N/A |  |  |  |  |  |  |  |  |  |
| #INFO: | QUERY: | ENSOARG00000019218 | CYTH4 | cytohesin 4 [Source:HGNC Symbol;Acc:HGNC:9505] | | | | |  |  |  |  |  |
| #INFO: | QUERY: | ENSOARG00000019231 | ELFN2 | extracellular leucine rich repeat and fibronectin type III domain containing 2 [Source:HGNC Symbol;Acc:HGNC:29396] | | | | | | | | | |
| #INFO: | QUERY: | ENSOARG00000019244 | GNAI3 | G protein subunit alpha i3 [Source:HGNC Symbol;Acc:HGNC:4387] | | | | | | |  |  |  |
| #INFO: | QUERY: | ENSOARG00000019262 | GNAT2 | G protein subunit alpha transducin 2 [Source:HGNC Symbol;Acc:HGNC:4394] | | | | | | | |  |  |
| #INFO: | QUERY: | ENSOARG00000019266 | AMPD2 | adenosine monophosphate deaminase 2 [Source:HGNC Symbol;Acc:HGNC:469] | | | | | | | |  |  |
| #INFO: | QUERY: | ENSOARG00000019276 | SLC9C1 | solute carrier family 9 member C1 [Source:HGNC Symbol;Acc:HGNC:31401] | | | | | | | |  |  |
| #INFO: | QUERY: | ENSOARG00000019295 | N/A | N/A |  |  |  |  |  |  |  |  |  |
| #INFO: | QUERY: | ENSOARG00000019298 | CD200 | CD200 molecule [Source:HGNC Symbol;Acc:HGNC:7203] | | | | | |  |  |  |  |
| #INFO: | QUERY: | ENSOARG00000019379 | BICD1 | BICD cargo adaptor 1 [Source:HGNC Symbol;Acc:HGNC:1049] | | | | | |  |  |  |  |
| #INFO: | QUERY: | ENSOARG00000019476 | N/A | N/A |  |  |  |  |  |  |  |  |  |
| #INFO: | QUERY: | ENSOARG00000019478 | N/A | N/A |  |  |  |  |  |  |  |  |  |
| #INFO: | QUERY: | ENSOARG00000019482 | N/A | N/A |  |  |  |  |  |  |  |  |  |
| #INFO: | QUERY: | ENSOARG00000019485 | N/A | N/A |  |  |  |  |  |  |  |  |  |
| #INFO: | QUERY: | ENSOARG00000019487 | N/A | N/A |  |  |  |  |  |  |  |  |  |
| #INFO: | QUERY: | ENSOARG00000019489 | N/A | N/A |  |  |  |  |  |  |  |  |  |
| #INFO: | QUERY: | ENSOARG00000019554 | ARID2 | AT-rich interaction domain 2 [Source:HGNC Symbol;Acc:HGNC:18037] | | | | | | |  |  |  |
| #INFO: | QUERY: | ENSOARG00000019660 | CCDC91 | coiled-coil domain containing 91 [Source:HGNC Symbol;Acc:HGNC:24855] | | | | | | | |  |  |
| #INFO: | QUERY: | ENSOARG00000019926 | INPP5B | inositol polyphosphate-5-phosphatase B [Source:HGNC Symbol;Acc:HGNC:6077] | | | | | | | |  |  |
| #INFO: | QUERY: | ENSOARG00000019950 | SF3A3 | splicing factor 3a subunit 3 [Source:HGNC Symbol;Acc:HGNC:10767] | | | | | | |  |  |  |
| #INFO: | QUERY: | ENSOARG00000019970 | LRRK2 | leucine rich repeat kinase 2 [Source:HGNC Symbol;Acc:HGNC:18618] | | | | | | |  |  |  |
| #INFO: | QUERY: | ENSOARG00000019974 | TSPYL4 | TSPY like 4 [Source:HGNC Symbol;Acc:HGNC:21559] | | | | |  |  |  |  |  |
| #INFO: | QUERY: | ENSOARG00000020011 | DPH6 | diphthamine biosynthesis 6 [Source:HGNC Symbol;Acc:HGNC:30543] | | | | | | |  |  |  |
| #INFO: | QUERY: | ENSOARG00000020017 | MEIS2 | Meis homeobox 2 [Source:HGNC Symbol;Acc:HGNC:7001] | | | | | |  |  |  |  |
| #INFO: | QUERY: | ENSOARG00000020083 | N/A | N/A |  |  |  |  |  |  |  |  |  |
| #INFO: | QUERY: | ENSOARG00000020110 | SOX5 | SRY-box 5 [Source:HGNC Symbol;Acc:HGNC:11201] | | | | |  |  |  |  |  |
| #INFO: | QUERY: | ENSOARG00000020117 | ETNK1 | ethanolamine kinase 1 [Source:HGNC Symbol;Acc:HGNC:24649] | | | | | | |  |  |  |
| #INFO: | QUERY: | ENSOARG00000020148 | RESP18 | regulated endocrine specific protein 18 [Source:HGNC Symbol;Acc:HGNC:33762] | | | | | | | |  |  |
| #INFO: | QUERY: | ENSOARG00000020161 | DNPEP | aspartyl aminopeptidase [Source:HGNC Symbol;Acc:HGNC:2981] | | | | | | |  |  |  |
| #INFO: | QUERY: | ENSOARG00000020178 | ST8SIA1 | ST8 alpha-N-acetyl-neuraminide alpha-2,8-sialyltransferase 1 [Source:HGNC Symbol;Acc:HGNC:10869] | | | | | | | | | |
| #INFO: | QUERY: | ENSOARG00000020343 | RECQL | RecQ like helicase [Source:HGNC Symbol;Acc:HGNC:9948] | | | | | |  |  |  |  |
| #INFO: | QUERY: | ENSOARG00000020366 | PYROXD1 | pyridine nucleotide-disulphide oxidoreductase domain 1 [Source:HGNC Symbol;Acc:HGNC:26162] | | | | | | | | | |
| #INFO: | QUERY: | ENSOARG00000020369 | IAPP | islet amyloid polypeptide [Source:HGNC Symbol;Acc:HGNC:5329] | | | | | | |  |  |  |
| #INFO: | QUERY: | ENSOARG00000020377 | DLG1 | discs large MAGUK scaffold protein 1 [Source:HGNC Symbol;Acc:HGNC:2900] | | | | | | | |  |  |
| #INFO: | QUERY: | ENSOARG00000020425 | N/A | N/A |  |  |  |  |  |  |  |  |  |
| #INFO: | QUERY: | ENSOARG00000020431 | N/A | N/A |  |  |  |  |  |  |  |  |  |
| #INFO: | QUERY: | ENSOARG00000020442 | NOTCH2 | notch 2 [Source:HGNC Symbol;Acc:HGNC:7882] | | | | |  |  |  |  |  |
| #INFO: | QUERY: | ENSOARG00000020460 | SEC22B | SEC22 homolog B, vesicle trafficking protein (gene/pseudogene) [Source:HGNC Symbol;Acc:HGNC:10700] | | | | | | | | | |
| #INFO: | QUERY: | ENSOARG00000020464 | N/A | N/A |  |  |  |  |  |  |  |  |  |
| #INFO: | QUERY: | ENSOARG00000020470 | N/A | N/A |  |  |  |  |  |  |  |  |  |
| #INFO: | QUERY: | ENSOARG00000020478 | TP63 | tumor protein p63 [Source:HGNC Symbol;Acc:HGNC:15979] | | | | | |  |  |  |  |
| #INFO: | QUERY: | ENSOARG00000020488 | VPS39 | VPS39, HOPS complex subunit [Source:HGNC Symbol;Acc:HGNC:20593] | | | | | | |  |  |  |
| #INFO: | QUERY: | ENSOARG00000020503 | COL4A4 | collagen type IV alpha 4 chain [Source:HGNC Symbol;Acc:HGNC:2206] | | | | | | |  |  |  |
| #INFO: | QUERY: | ENSOARG00000020510 | ZNF106 | zinc finger protein 106 [Source:HGNC Symbol;Acc:HGNC:12886] | | | | | | |  |  |  |
| #INFO: | QUERY: | ENSOARG00000020521 | GANC | glucosidase alpha, neutral C [Source:HGNC Symbol;Acc:HGNC:4139] | | | | | | |  |  |  |
| #INFO: | QUERY: | ENSOARG00000020525 | COL4A3 | collagen type IV alpha 3 chain [Source:HGNC Symbol;Acc:HGNC:2204] | | | | | | |  |  |  |
| #INFO: | QUERY: | ENSOARG00000020604 | ECE2 | endothelin converting enzyme 2 [Source:HGNC Symbol;Acc:HGNC:13275] | | | | | | | |  |  |
| #INFO: | QUERY: | ENSOARG00000020606 | ALG3 | ALG3, alpha-1,3- mannosyltransferase [Source:HGNC Symbol;Acc:HGNC:23056] | | | | | | | |  |  |
| #INFO: | QUERY: | ENSOARG00000020609 | VWA5B2 | von Willebrand factor A domain containing 5B2 [Source:HGNC Symbol;Acc:HGNC:25144] | | | | | | | | |  |
| #INFO: | QUERY: | ENSOARG00000020612 | ABCF3 | ATP binding cassette subfamily F member 3 [Source:HGNC Symbol;Acc:HGNC:72] | | | | | | | |  |  |
| #INFO: | QUERY: | ENSOARG00000020675 | SPATA3 | spermatogenesis associated 3 [Source:HGNC Symbol;Acc:HGNC:17884] | | | | | | |  |  |  |
| #INFO: | QUERY: | ENSOARG00000020699 | FRMD6 | FERM domain containing 6 [Source:HGNC Symbol;Acc:HGNC:19839] | | | | | | |  |  |  |
| #INFO: | QUERY: | ENSOARG00000020802 | GRIN2B | glutamate ionotropic receptor NMDA type subunit 2B [Source:HGNC Symbol;Acc:HGNC:4586] | | | | | | | | |  |
| #INFO: | QUERY: | ENSOARG00000020869 | CTSK | cathepsin K [Source:HGNC Symbol;Acc:HGNC:2536] | | | | |  |  |  |  |  |
| #INFO: | QUERY: | ENSOARG00000020870 | ARNT | aryl hydrocarbon receptor nuclear translocator [Source:RefSeq peptide;Acc:NP_001274394] | | | | | | | | |  |
| #INFO: | QUERY: | ENSOARG00000020954 | MAPK6 | mitogen-activated protein kinase 6 [Source:HGNC Symbol;Acc:HGNC:6879] | | | | | | | |  |  |
| #INFO: | QUERY: | ENSOARG00000020981 | SPPL2A | signal peptide peptidase like 2A [Source:HGNC Symbol;Acc:HGNC:30227] | | | | | | | |  |  |
| #INFO: | QUERY: | ENSOARG00000020984 | TRPM7 | transient receptor potential cation channel subfamily M member 7 [Source:RefSeq peptide;Acc:NP_001087254] | | | | | | | | | |
| #INFO: | QUERY: | ENSOARG00000021205 | U6 | U6 spliceosomal RNA [Source:RFAM;Acc:RF00026] | | | | |  |  |  |  |  |
| #INFO: | QUERY: | ENSOARG00000021252 | U4 | U4 spliceosomal RNA [Source:RFAM;Acc:RF00015] | | | | |  |  |  |  |  |
| #INFO: | QUERY: | ENSOARG00000021266 | N/A | N/A |  |  |  |  |  |  |  |  |  |
| #INFO: | QUERY: | ENSOARG00000021294 | 5S_RRNA | 5S ribosomal RNA [Source:RFAM;Acc:RF00001] | | | | |  |  |  |  |  |
| #INFO: | QUERY: | ENSOARG00000021301 | SNORA70 | Small nucleolar RNA SNORA70 [Source:RFAM;Acc:RF00156] | | | | | |  |  |  |  |
| #INFO: | QUERY: | ENSOARG00000021309 | 5S_RRNA | 5S ribosomal RNA [Source:RFAM;Acc:RF00001] | | | | |  |  |  |  |  |
| #INFO: | QUERY: | ENSOARG00000021378 | U4 | U4 spliceosomal RNA [Source:RFAM;Acc:RF00015] | | | | |  |  |  |  |  |
| #INFO: | QUERY: | ENSOARG00000021387 | SNORA46 | Small nucleolar RNA SNORA46 [Source:RFAM;Acc:RF00404] | | | | | |  |  |  |  |
| #INFO: | QUERY: | ENSOARG00000021493 | N/A | N/A |  |  |  |  |  |  |  |  |  |
| #INFO: | QUERY: | ENSOARG00000021595 | 5S_RRNA | 5S ribosomal RNA [Source:RFAM;Acc:RF00001] | | | | |  |  |  |  |  |
| #INFO: | QUERY: | ENSOARG00000021600 | SNORA9 | Small nucleolar RNA SNORA9 [Source:RFAM;Acc:RF00411] | | | | | |  |  |  |  |
| #INFO: | QUERY: | ENSOARG00000021647 | PVT1_2 | Pvt1 oncogene conserved region 2 [Source:RFAM;Acc:RF02165] | | | | | | |  |  |  |
| #INFO: | QUERY: | ENSOARG00000021681 | U6 | U6 spliceosomal RNA [Source:RFAM;Acc:RF00026] | | | | |  |  |  |  |  |
| #INFO: | QUERY: | ENSOARG00000021745 | U6 | U6 spliceosomal RNA [Source:RFAM;Acc:RF00026] | | | | |  |  |  |  |  |
| #INFO: | QUERY: | ENSOARG00000021766 | N/A | N/A |  |  |  |  |  |  |  |  |  |
| #INFO: | QUERY: | ENSOARG00000021971 | 7SK | 7SK RNA [Source:RFAM;Acc:RF00100] | | | |  |  |  |  |  |  |
| #INFO: | QUERY: | ENSOARG00000021987 | U4 | U4 spliceosomal RNA [Source:RFAM;Acc:RF00015] | | | | |  |  |  |  |  |
| #INFO: | QUERY: | ENSOARG00000021998 | SNORA40 | Small nucleolar RNA SNORA40 [Source:RFAM;Acc:RF00561] | | | | | |  |  |  |  |
| #INFO: | QUERY: | ENSOARG00000022003 | N/A | N/A |  |  |  |  |  |  |  |  |  |
| #INFO: | QUERY: | ENSOARG00000022097 | U6 | U6 spliceosomal RNA [Source:RFAM;Acc:RF00026] | | | | |  |  |  |  |  |
| #INFO: | QUERY: | ENSOARG00000022139 | N/A | N/A |  |  |  |  |  |  |  |  |  |
| #INFO: | QUERY: | ENSOARG00000022167 | 5S_RRNA | 5S ribosomal RNA [Source:RFAM;Acc:RF00001] | | | | |  |  |  |  |  |
| #INFO: | QUERY: | ENSOARG00000022212 | N/A | N/A |  |  |  |  |  |  |  |  |  |
| #INFO: | QUERY: | ENSOARG00000022220 | N/A | N/A |  |  |  |  |  |  |  |  |  |
| #INFO: | QUERY: | ENSOARG00000022236 | N/A | N/A |  |  |  |  |  |  |  |  |  |
| #INFO: | QUERY: | ENSOARG00000022321 | CPEB3_RIBOZYME | Mammalian CPEB3 ribozyme [Source:RFAM;Acc:RF00622] | | | | | |  |  |  |  |
| #INFO: | QUERY: | ENSOARG00000022437 | N/A | N/A |  |  |  |  |  |  |  |  |  |
| #INFO: | QUERY: | ENSOARG00000022467 | SNORA76 | Small nucleolar RNA SNORA76 [Source:RFAM;Acc:RF00598] | | | | | |  |  |  |  |
| #INFO: | QUERY: | ENSOARG00000022481 | N/A | N/A |  |  |  |  |  |  |  |  |  |
| #INFO: | QUERY: | ENSOARG00000022504 | 5S_RRNA | 5S ribosomal RNA [Source:RFAM;Acc:RF00001] | | | | |  |  |  |  |  |
| #INFO: | QUERY: | ENSOARG00000022512 | N/A | N/A |  |  |  |  |  |  |  |  |  |
| #INFO: | QUERY: | ENSOARG00000022627 | 7SK | 7SK RNA [Source:RFAM;Acc:RF00100] | | | |  |  |  |  |  |  |
| #INFO: | QUERY: | ENSOARG00000022706 | SNORA19 | Small nucleolar RNA SNORA19 [Source:RFAM;Acc:RF00413] | | | | | |  |  |  |  |
| #INFO: | QUERY: | ENSOARG00000022914 | SNORA77 | Small nucleolar RNA SNORA77 [Source:RFAM;Acc:RF00599] | | | | | |  |  |  |  |
| #INFO: | QUERY: | ENSOARG00000022967 | U6 | U6 spliceosomal RNA [Source:RFAM;Acc:RF00026] | | | | |  |  |  |  |  |
| #INFO: | QUERY: | ENSOARG00000022987 | SNORD72 | Small nucleolar RNA SNORD72 [Source:RFAM;Acc:RF00577] | | | | | |  |  |  |  |
| #INFO: | QUERY: | ENSOARG00000023147 | N/A | N/A |  |  |  |  |  |  |  |  |  |
| #INFO: | QUERY: | ENSOARG00000023188 | N/A | N/A |  |  |  |  |  |  |  |  |  |
| #INFO: | QUERY: | ENSOARG00000023242 | N/A | N/A |  |  |  |  |  |  |  |  |  |
| #INFO: | QUERY: | ENSOARG00000023421 | N/A | N/A |  |  |  |  |  |  |  |  |  |
| #INFO: | QUERY: | ENSOARG00000023444 | U6 | U6 spliceosomal RNA [Source:RFAM;Acc:RF00026] | | | | |  |  |  |  |  |
| #INFO: | QUERY: | ENSOARG00000023530 | N/A | N/A |  |  |  |  |  |  |  |  |  |
| #INFO: | QUERY: | ENSOARG00000023668 | U6 | U6 spliceosomal RNA [Source:RFAM;Acc:RF00026] | | | | |  |  |  |  |  |
| #INFO: | QUERY: | ENSOARG00000023681 | SNORA70 | Small nucleolar RNA SNORA70 [Source:RFAM;Acc:RF00156] | | | | | |  |  |  |  |
| #INFO: | QUERY: | ENSOARG00000023735 | OAR-MIR-191 | oar-mir-191 [Source:miRBase;Acc:MI0025260] | | | | |  |  |  |  |  |
| #INFO: | QUERY: | ENSOARG00000023752 | SNORD22 | Small nucleolar RNA SNORD22 [Source:RFAM;Acc:RF00099] | | | | | |  |  |  |  |
| #INFO: | QUERY: | ENSOARG00000023853 | N/A | N/A |  |  |  |  |  |  |  |  |  |
| #INFO: | QUERY: | ENSOARG00000023859 | U6 | U6 spliceosomal RNA [Source:RFAM;Acc:RF00026] | | | | |  |  |  |  |  |
| #INFO: | QUERY: | ENSOARG00000023860 | 5S_RRNA | 5S ribosomal RNA [Source:RFAM;Acc:RF00001] | | | | |  |  |  |  |  |
| #INFO: | QUERY: | ENSOARG00000023881 | SNOU13 | Small nucleolar RNA U13 [Source:RFAM;Acc:RF01210] | | | | | |  |  |  |  |
| #INFO: | QUERY: | ENSOARG00000024054 | N/A | N/A |  |  |  |  |  |  |  |  |  |
| #INFO: | QUERY: | ENSOARG00000024091 | PVT1_4 | Pvt1 oncogene conserved region 4 [Source:RFAM;Acc:RF02167] | | | | | | |  |  |  |
| #INFO: | QUERY: | ENSOARG00000024205 | U5 | U5 spliceosomal RNA [Source:RFAM;Acc:RF00020] | | | | |  |  |  |  |  |
| #INFO: | QUERY: | ENSOARG00000024249 | U6 | U6 spliceosomal RNA [Source:RFAM;Acc:RF00026] | | | | |  |  |  |  |  |
| #INFO: | QUERY: | ENSOARG00000024250 | U6 | U6 spliceosomal RNA [Source:RFAM;Acc:RF00026] | | | | |  |  |  |  |  |
| #INFO: | QUERY: | ENSOARG00000024274 | N/A | N/A |  |  |  |  |  |  |  |  |  |
| #INFO: | QUERY: | ENSOARG00000024382 | N/A | N/A |  |  |  |  |  |  |  |  |  |
| #INFO: | QUERY: | ENSOARG00000024390 | PVT1_5 | Pvt1 oncogene conserved region 5 [Source:RFAM;Acc:RF02168] | | | | | | |  |  |  |
| #INFO: | QUERY: | ENSOARG00000024611 | SNORA70 | Small nucleolar RNA SNORA70 [Source:RFAM;Acc:RF00156] | | | | | |  |  |  |  |
| #INFO: | QUERY: | ENSOARG00000024618 | PCGEM1 | Prostate-specific transcript 1 [Source:RFAM;Acc:RF01981] | | | | | |  |  |  |  |
| #INFO: | QUERY: | ENSOARG00000024648 | U6 | U6 spliceosomal RNA [Source:RFAM;Acc:RF00026] | | | | |  |  |  |  |  |
| #INFO: | QUERY: | ENSOARG00000024730 | U1 | U1 spliceosomal RNA [Source:RFAM;Acc:RF00003] | | | | |  |  |  |  |  |
| #INFO: | QUERY: | ENSOARG00000024741 | U6 | U6 spliceosomal RNA [Source:RFAM;Acc:RF00026] | | | | |  |  |  |  |  |
| #INFO: | QUERY: | ENSOARG00000024743 | U2 | U2 spliceosomal RNA [Source:RFAM;Acc:RF00004] | | | | |  |  |  |  |  |
| #INFO: | QUERY: | ENSOARG00000024772 | UC_338 | TUC338 [Source:RFAM;Acc:RF02271] | | | |  |  |  |  |  |  |
| #INFO: | QUERY: | ENSOARG00000024799 | N/A | N/A |  |  |  |  |  |  |  |  |  |
| #INFO: | QUERY: | ENSOARG00000024834 | SNORA70 | Small nucleolar RNA SNORA70 [Source:RFAM;Acc:RF00156] | | | | | |  |  |  |  |
| #INFO: | QUERY: | ENSOARG00000024840 | U6 | U6 spliceosomal RNA [Source:RFAM;Acc:RF00026] | | | | |  |  |  |  |  |
| #INFO: | QUERY: | ENSOARG00000025021 | N/A | N/A |  |  |  |  |  |  |  |  |  |
| #INFO: | QUERY: | ENSOARG00000025048 | N/A | N/A |  |  |  |  |  |  |  |  |  |
| #INFO: | QUERY: | ENSOARG00000025126 | N/A | N/A |  |  |  |  |  |  |  |  |  |
| #INFO: | QUERY: | ENSOARG00000025230 | N/A | N/A |  |  |  |  |  |  |  |  |  |
| #INFO: | QUERY: | ENSOARG00000025258 | N/A | N/A |  |  |  |  |  |  |  |  |  |
| #INFO: | QUERY: | ENSOARG00000025290 | N/A | N/A |  |  |  |  |  |  |  |  |  |
| #INFO: | QUERY: | ENSOARG00000025293 | N/A | N/A |  |  |  |  |  |  |  |  |  |
| #INFO: | QUERY: | ENSOARG00000025309 | N/A | N/A |  |  |  |  |  |  |  |  |  |
| #INFO: | QUERY: | ENSOARG00000025447 | N/A | N/A |  |  |  |  |  |  |  |  |  |
| #INFO: | QUERY: | ENSOARG00000025448 | N/A | N/A |  |  |  |  |  |  |  |  |  |
| #INFO: | QUERY: | ENSOARG00000025465 | N/A | N/A |  |  |  |  |  |  |  |  |  |
| #INFO: | QUERY: | ENSOARG00000025676 | N/A | N/A |  |  |  |  |  |  |  |  |  |
| #INFO: | QUERY: | ENSOARG00000025695 | N/A | N/A |  |  |  |  |  |  |  |  |  |
| #INFO: | QUERY: | ENSOARG00000025724 | N/A | N/A |  |  |  |  |  |  |  |  |  |
| #INFO: | QUERY: | ENSOARG00000025725 | N/A | N/A |  |  |  |  |  |  |  |  |  |
| #INFO: | QUERY: | ENSOARG00000025754 | N/A | N/A |  |  |  |  |  |  |  |  |  |
| #INFO: | QUERY: | ENSOARG00000025774 | N/A | N/A |  |  |  |  |  |  |  |  |  |
| #INFO: | QUERY: | ENSOARG00000025798 | N/A | N/A |  |  |  |  |  |  |  |  |  |
| #INFO: | QUERY: | ENSOARG00000025821 | N/A | N/A |  |  |  |  |  |  |  |  |  |
| #INFO: | QUERY: | ENSOARG00000025830 | N/A | N/A |  |  |  |  |  |  |  |  |  |
| #INFO: | QUERY: | ENSOARG00000025937 | N/A | N/A |  |  |  |  |  |  |  |  |  |
| #INFO: | QUERY: | ENSOARG00000025938 | N/A | N/A |  |  |  |  |  |  |  |  |  |
| #INFO: | QUERY: | ENSOARG00000025947 | N/A | N/A |  |  |  |  |  |  |  |  |  |
| #INFO: | QUERY: | ENSOARG00000025977 | N/A | N/A |  |  |  |  |  |  |  |  |  |
| #INFO: | QUERY: | ENSOARG00000026004 | N/A | N/A |  |  |  |  |  |  |  |  |  |
| #INFO: | QUERY: | ENSOARG00000026014 | N/A | N/A |  |  |  |  |  |  |  |  |  |
| #INFO: | QUERY: | ENSOARG00000026026 | N/A | N/A |  |  |  |  |  |  |  |  |  |
| #INFO: | QUERY: | ENSOARG00000026030 | N/A | N/A |  |  |  |  |  |  |  |  |  |
| #INFO: | QUERY: | ENSOARG00000026065 | N/A | N/A |  |  |  |  |  |  |  |  |  |
| #INFO: | QUERY: | ENSOARG00000026222 | N/A | N/A |  |  |  |  |  |  |  |  |  |
| #INFO: | QUERY: | ENSOARG00000026240 | N/A | N/A |  |  |  |  |  |  |  |  |  |
| #INFO: | QUERY: | ENSOARG00000026271 | N/A | N/A |  |  |  |  |  |  |  |  |  |
| #INFO: | QUERY: | ENSOARG00000026272 | N/A | N/A |  |  |  |  |  |  |  |  |  |
| #INFO: | QUERY: | ENSOARG00000026305 | N/A | N/A |  |  |  |  |  |  |  |  |  |
| #INFO: | QUERY: | ENSOARG00000026306 | N/A | N/A |  |  |  |  |  |  |  |  |  |
| #INFO: | QUERY: | ENSOARG00000026377 | N/A | N/A |  |  |  |  |  |  |  |  |  |
| #INFO: | QUERY: | ENSOARG00000026392 | N/A | N/A |  |  |  |  |  |  |  |  |  |
| #INFO: | QUERY: | ENSOARG00000026406 | N/A | N/A |  |  |  |  |  |  |  |  |  |
| #INFO: | QUERY: | ENSOARG00000026407 | N/A | N/A |  |  |  |  |  |  |  |  |  |
| #INFO: | QUERY: | ENSOARG00000026415 | N/A | N/A |  |  |  |  |  |  |  |  |  |
| #INFO: | QUERY: | ENSOARG00000026456 | N/A | N/A |  |  |  |  |  |  |  |  |  |
| #INFO: | QUERY: | ENSOARG00000026640 | N/A | N/A |  |  |  |  |  |  |  |  |  |
| #INFO: | QUERY: | ENSOARG00000026661 | N/A | N/A |  |  |  |  |  |  |  |  |  |
| #INFO: | QUERY: | ENSOARG00000026721 | N/A | N/A |  |  |  |  |  |  |  |  |  |
| #INFO: | QUERY: | ENSOARG00000026829 | N/A | N/A |  |  |  |  |  |  |  |  |  |
| #INFO: | QUERY: | ENSOARG00000027023 | N/A | N/A |  |  |  |  |  |  |  |  |  |
| #INFO: | QUERY: | ENSOARG00000027027 | N/A | N/A |  |  |  |  |  |  |  |  |  |
| #INFO: | QUERY: | ENSOARG00000027055 | N/A | N/A |  |  |  |  |  |  |  |  |  |
| #INFO: | QUERY: | ENSOARG00000027056 | N/A | N/A |  |  |  |  |  |  |  |  |  |
| #INFO: | QUERY: | ENSOARG00000027057 | N/A | N/A |  |  |  |  |  |  |  |  |  |
