# Supplementary material for: Detection of candidate genes affecting milk production traits in sheep using whole‐genome sequencing analysis
Source: Vet Med Sci. 2022 Jan 11;8(3):1197–204. doi: 10.1002/vms3.731 (PMC9122411; doi:10.1002/vms3.731)
Supplement: Supplementary file 3 — TABLE S2 Positively selected genes extracted with nucleotide diversity (Pi) statistical method and output of g: profiler related to them [file VMS3-8-1197-s005.docx]

**Table S2.** Positively selected genes extracted with nucleotide diversity (Pi) statistical method and output of g: profiler related to them

| # | signf | | p-value | | T | | Q | | Q&T | | Q&T/Q | | Q&T/T | | term ID | | t type | | t group | | t name | | t depth | | Q&T list |
| --- | --- | --- | --- | --- | --- | --- | --- | --- | --- | --- | --- | --- | --- | --- | --- | --- | --- | --- | --- | --- | --- | --- | --- | --- | --- |
| # |  | |  | |  | |  | |  | |  | |  | |  | |  | |  | |  | |  | |  |
| 1 | ! | | 0.0415 | | 70 | | 328 | | 7 | | 0.021 | | 0.1 | | GO:0071774 | | BP | | 14 | | response to fibroblast growth factor | | 1 | | ENSOARG00000003329,ENSOARG00000007954,ENSOARG00000010131,ENSOARG00000010198,ENSOARG00000014240,ENSOARG00000017222,ENSOARG00000018809 |
| 1 | ! | | 0.0415 | | 70 | | 328 | | 7 | | 0.021 | | 0.1 | | GO:0044344 | | BP | | 14 | | cellular response to fibroblast growth factor stimulus | | 1 | | ENSOARG00000003329,ENSOARG00000007954,ENSOARG00000010131,ENSOARG00000010198,ENSOARG00000014240,ENSOARG00000017222,ENSOARG00000018809 |
| 1 | ! | | 0.0442 | | 2 | | 328 | | 2 | | 0.006 | | 1 | | GO:0032025 | | BP | | 5 | | response to cobalt ion | | 1 | | ENSOARG00000010271,ENSOARG00000015367 |
| 1 | ! | | 0.0442 | | 2 | | 328 | | 2 | | 0.006 | | 1 | | GO:0071279 | | BP | | 5 | | cellular response to cobalt ion | | 1 | | ENSOARG00000010271,ENSOARG00000015367 |
| 1 | ! | | 0.0442 | | 2 | | 328 | | 2 | | 0.006 | | 1 | | GO:0033563 | | BP | | 11 | | dorsal/ventral axon guidance | | 1 | | ENSOARG00000004732,ENSOARG00000008959 |
| 1 | ! | | 0.023 | | 43 | | 328 | | 6 | | 0.018 | | 0.14 | | GO:0034637 | | BP | | 13 | | cellular carbohydrate biosynthetic process | | 1 | | ENSOARG00000003933,ENSOARG00000004634,ENSOARG00000009854,ENSOARG00000013995,ENSOARG00000014188,ENSOARG00000017762 |
| 1 | ! | | 0.0147 | | 39 | | 328 | | 6 | | 0.018 | | 0.154 | | GO:0000271 | | BP | | 13 | | polysaccharide biosynthetic process | | 1 | | ENSOARG00000003933,ENSOARG00000004634,ENSOARG00000009854,ENSOARG00000013995,ENSOARG00000014188,ENSOARG00000017762 |
| 1 | ! | | 0.0102 | | 35 | | 328 | | 6 | | 0.018 | | 0.171 | | GO:0033692 | | BP | | 13 | | cellular polysaccharide biosynthetic process | | 2 | | ENSOARG00000003933,ENSOARG00000004634,ENSOARG00000009854,ENSOARG00000013995,ENSOARG00000014188,ENSOARG00000017762 |
| 1 | ! | | 0.026 | | 1196 | | 328 | | 40 | | 0.122 | | 0.033 | | GO:0007186 | | BP | | 3 | | G-protein coupled receptor signaling pathway | | 1 | | ENSOARG00000000204,ENSOARG00000000238,ENSOARG00000000325,ENSOARG00000001126,ENSOARG00000001342,ENSOARG00000003137,ENSOARG00000003825,ENSOARG00000004069,ENSOARG00000004080,ENSOARG00000004093,ENSOARG00000004105,ENSOARG00000004748,ENSOARG00000006182,ENSOARG00000006204,ENSOARG00000006696,ENSOARG00000007147,ENSOARG00000007336,ENSOARG00000007355,ENSOARG00000007372,ENSOARG00000007386,ENSOARG00000007531,ENSOARG00000007569,ENSOARG00000007583,ENSOARG00000008979,ENSOARG00000011621,ENSOARG00000013529,ENSOARG00000013620,ENSOARG00000013925,ENSOARG00000014001,ENSOARG00000015761,ENSOARG00000016502,ENSOARG00000017215,ENSOARG00000017228,ENSOARG00000017361,ENSOARG00000017887,ENSOARG00000018287,ENSOARG00000018363,ENSOARG00000018380,ENSOARG00000019176,ENSOARG00000019747 |
| 1 | ! | | 0.0102 | | 1524 | | 328 | | 50 | | 0.152 | | 0.033 | | GO:0003008 | | BP | | 10 | | system process | | 1 | | ENSOARG00000000204,ENSOARG00000000238,ENSOARG00000000325,ENSOARG00000000603,ENSOARG00000000681,ENSOARG00000001126,ENSOARG00000001342,ENSOARG00000002168,ENSOARG00000003137,ENSOARG00000003329,ENSOARG00000004069,ENSOARG00000004080,ENSOARG00000004093,ENSOARG00000004105,ENSOARG00000004748,ENSOARG00000005168,ENSOARG00000006182,ENSOARG00000006204,ENSOARG00000006696,ENSOARG00000007336,ENSOARG00000007355,ENSOARG00000007372,ENSOARG00000007386,ENSOARG00000007531,ENSOARG00000007560,ENSOARG00000007569,ENSOARG00000007583,ENSOARG00000007585,ENSOARG00000008979,ENSOARG00000009678,ENSOARG00000010714,ENSOARG00000011621,ENSOARG00000011860,ENSOARG00000012452,ENSOARG00000013009,ENSOARG00000015124,ENSOARG00000015761,ENSOARG00000016010,ENSOARG00000016502,ENSOARG00000016960,ENSOARG00000017012,ENSOARG00000017215,ENSOARG00000017228,ENSOARG00000018241,ENSOARG00000018287,ENSOARG00000018363,ENSOARG00000018380,ENSOARG00000018985,ENSOARG00000019007,ENSOARG00000019176 |
| 1 | ! | | 0.00261 | | 1135 | | 328 | | 43 | | 0.131 | | 0.038 | | GO:0050877 | | BP | | 10 | | nervous system process | | 2 | | ENSOARG00000000204,ENSOARG00000000238,ENSOARG00000000325,ENSOARG00000001126,ENSOARG00000001342,ENSOARG00000002168,ENSOARG00000003137,ENSOARG00000004069,ENSOARG00000004080,ENSOARG00000004093,ENSOARG00000004105,ENSOARG00000004748,ENSOARG00000005168,ENSOARG00000006182,ENSOARG00000006204,ENSOARG00000006696,ENSOARG00000007336,ENSOARG00000007355,ENSOARG00000007372,ENSOARG00000007386,ENSOARG00000007531,ENSOARG00000007569,ENSOARG00000007583,ENSOARG00000007585,ENSOARG00000008979,ENSOARG00000009678,ENSOARG00000010714,ENSOARG00000011621,ENSOARG00000011860,ENSOARG00000012452,ENSOARG00000013009,ENSOARG00000015124,ENSOARG00000016010,ENSOARG00000016502,ENSOARG00000017215,ENSOARG00000017228,ENSOARG00000018241,ENSOARG00000018287,ENSOARG00000018363,ENSOARG00000018380,ENSOARG00000018985,ENSOARG00000019007,ENSOARG00000019176 |
| 1 | ! | | 0.0017 | | 907 | | 328 | | 38 | | 0.116 | | 0.042 | | GO:0007600 | | BP | | 10 | | sensory perception | | 3 | | ENSOARG00000000204,ENSOARG00000000238,ENSOARG00000000325,ENSOARG00000001126,ENSOARG00000001342,ENSOARG00000002168,ENSOARG00000003137,ENSOARG00000004069,ENSOARG00000004080,ENSOARG00000004093,ENSOARG00000004105,ENSOARG00000004748,ENSOARG00000006182,ENSOARG00000006204,ENSOARG00000006696,ENSOARG00000007336,ENSOARG00000007355,ENSOARG00000007372,ENSOARG00000007386,ENSOARG00000007531,ENSOARG00000007569,ENSOARG00000007583,ENSOARG00000008979,ENSOARG00000010714,ENSOARG00000011621,ENSOARG00000011860,ENSOARG00000013009,ENSOARG00000015124,ENSOARG00000016010,ENSOARG00000016502,ENSOARG00000017215,ENSOARG00000017228,ENSOARG00000018287,ENSOARG00000018363,ENSOARG00000018380,ENSOARG00000018985,ENSOARG00000019007,ENSOARG00000019176 |
| 1 | ! | | 0.00474 | | 660 | | 328 | | 29 | | 0.088 | | 0.044 | | GO:0007606 | | BP | | 10 | | sensory perception of chemical stimulus | | 4 | | ENSOARG00000000204,ENSOARG00000000238,ENSOARG00000000325,ENSOARG00000001126,ENSOARG00000001342,ENSOARG00000003137,ENSOARG00000004069,ENSOARG00000004080,ENSOARG00000004093,ENSOARG00000004105,ENSOARG00000004748,ENSOARG00000006182,ENSOARG00000006204,ENSOARG00000006696,ENSOARG00000007336,ENSOARG00000007355,ENSOARG00000007372,ENSOARG00000007386,ENSOARG00000007531,ENSOARG00000007569,ENSOARG00000007583,ENSOARG00000008979,ENSOARG00000011621,ENSOARG00000017215,ENSOARG00000017228,ENSOARG00000018287,ENSOARG00000018363,ENSOARG00000018380,ENSOARG00000019176 |
| 1 | ! | | 0.00232 | | 612 | | 328 | | 29 | | 0.088 | | 0.047 | | GO:0007608 | | BP | | 10 | | sensory perception of smell | | 5 | | ENSOARG00000000204,ENSOARG00000000238,ENSOARG00000000325,ENSOARG00000001126,ENSOARG00000001342,ENSOARG00000003137,ENSOARG00000004069,ENSOARG00000004080,ENSOARG00000004093,ENSOARG00000004105,ENSOARG00000004748,ENSOARG00000006182,ENSOARG00000006204,ENSOARG00000006696,ENSOARG00000007336,ENSOARG00000007355,ENSOARG00000007372,ENSOARG00000007386,ENSOARG00000007531,ENSOARG00000007569,ENSOARG00000007583,ENSOARG00000008979,ENSOARG00000011621,ENSOARG00000017215,ENSOARG00000017228,ENSOARG00000018287,ENSOARG00000018363,ENSOARG00000018380,ENSOARG00000019176 |
| 1 | ! | | 0.00319 | | 713 | | 328 | | 31 | | 0.095 | | 0.043 | | GO:0051606 | | BP | | 10 | | detection of stimulus | | 1 | | ENSOARG00000000204,ENSOARG00000000238,ENSOARG00000000325,ENSOARG00000001126,ENSOARG00000001342,ENSOARG00000003137,ENSOARG00000004069,ENSOARG00000004080,ENSOARG00000004093,ENSOARG00000004105,ENSOARG00000004748,ENSOARG00000006182,ENSOARG00000006204,ENSOARG00000006696,ENSOARG00000007336,ENSOARG00000007355,ENSOARG00000007372,ENSOARG00000007386,ENSOARG00000007531,ENSOARG00000007569,ENSOARG00000007583,ENSOARG00000008979,ENSOARG00000011621,ENSOARG00000013009,ENSOARG00000015124,ENSOARG00000017215,ENSOARG00000017228,ENSOARG00000018287,ENSOARG00000018363,ENSOARG00000018380,ENSOARG00000019176 |
| 1 | ! | | 0.00304 | | 634 | | 328 | | 29 | | 0.088 | | 0.046 | | GO:0009593 | | BP | | 10 | | detection of chemical stimulus | | 2 | | ENSOARG00000000204,ENSOARG00000000238,ENSOARG00000000325,ENSOARG00000001126,ENSOARG00000001342,ENSOARG00000003137,ENSOARG00000004069,ENSOARG00000004080,ENSOARG00000004093,ENSOARG00000004105,ENSOARG00000004748,ENSOARG00000006182,ENSOARG00000006204,ENSOARG00000006696,ENSOARG00000007336,ENSOARG00000007355,ENSOARG00000007372,ENSOARG00000007386,ENSOARG00000007531,ENSOARG00000007569,ENSOARG00000007583,ENSOARG00000008979,ENSOARG00000011621,ENSOARG00000017215,ENSOARG00000017228,ENSOARG00000018287,ENSOARG00000018363,ENSOARG00000018380,ENSOARG00000019176 |
| 1 | ! | | 0.0017 | | 657 | | 328 | | 31 | | 0.095 | | 0.047 | | GO:0050906 | | BP | | 10 | | detection of stimulus involved in sensory perception | | 2 | | ENSOARG00000000204,ENSOARG00000000238,ENSOARG00000000325,ENSOARG00000001126,ENSOARG00000001342,ENSOARG00000003137,ENSOARG00000004069,ENSOARG00000004080,ENSOARG00000004093,ENSOARG00000004105,ENSOARG00000004748,ENSOARG00000006182,ENSOARG00000006204,ENSOARG00000006696,ENSOARG00000007336,ENSOARG00000007355,ENSOARG00000007372,ENSOARG00000007386,ENSOARG00000007531,ENSOARG00000007569,ENSOARG00000007583,ENSOARG00000008979,ENSOARG00000011621,ENSOARG00000013009,ENSOARG00000015124,ENSOARG00000017215,ENSOARG00000017228,ENSOARG00000018287,ENSOARG00000018363,ENSOARG00000018380,ENSOARG00000019176 |
| 1 | ! | | 0.00232 | | 616 | | 328 | | 29 | | 0.088 | | 0.047 | | GO:0050907 | | BP | | 10 | | detection of chemical stimulus involved in sensory perception | | 3 | | ENSOARG00000000204,ENSOARG00000000238,ENSOARG00000000325,ENSOARG00000001126,ENSOARG00000001342,ENSOARG00000003137,ENSOARG00000004069,ENSOARG00000004080,ENSOARG00000004093,ENSOARG00000004105,ENSOARG00000004748,ENSOARG00000006182,ENSOARG00000006204,ENSOARG00000006696,ENSOARG00000007336,ENSOARG00000007355,ENSOARG00000007372,ENSOARG00000007386,ENSOARG00000007531,ENSOARG00000007569,ENSOARG00000007583,ENSOARG00000008979,ENSOARG00000011621,ENSOARG00000017215,ENSOARG00000017228,ENSOARG00000018287,ENSOARG00000018363,ENSOARG00000018380,ENSOARG00000019176 |
| 1 | ! | | 0.0017 | | 592 | | 328 | | 29 | | 0.088 | | 0.049 | | GO:0050911 | | BP | | 10 | | detection of chemical stimulus involved in sensory perception of smell | | 4 | | ENSOARG00000000204,ENSOARG00000000238,ENSOARG00000000325,ENSOARG00000001126,ENSOARG00000001342,ENSOARG00000003137,ENSOARG00000004069,ENSOARG00000004080,ENSOARG00000004093,ENSOARG00000004105,ENSOARG00000004748,ENSOARG00000006182,ENSOARG00000006204,ENSOARG00000006696,ENSOARG00000007336,ENSOARG00000007355,ENSOARG00000007372,ENSOARG00000007386,ENSOARG00000007531,ENSOARG00000007569,ENSOARG00000007583,ENSOARG00000008979,ENSOARG00000011621,ENSOARG00000017215,ENSOARG00000017228,ENSOARG00000018287,ENSOARG00000018363,ENSOARG00000018380,ENSOARG00000019176 |
| 1 | ! | | 0.0191 | | 14 | | 328 | | 4 | | 0.012 | | 0.286 | | GO:0015669 | | BP | | 2 | | gas transport | | 1 | | ENSOARG00000019130,ENSOARG00000019144,ENSOARG00000019163,ENSOARG00000019174 |
| 1 | ! | | 0.0102 | | 11 | | 328 | | 4 | | 0.012 | | 0.364 | | GO:0015671 | | BP | | 2 | | oxygen transport | | 2 | | ENSOARG00000019130,ENSOARG00000019144,ENSOARG00000019163,ENSOARG00000019174 |
| 1 | ! | | 0.00319 | | 8 | | 328 | | 4 | | 0.012 | | 0.5 | | GO:0005833 | | CC | | 17 | | hemoglobin complex | | 1 | | ENSOARG00000019130,ENSOARG00000019144,ENSOARG00000019163,ENSOARG00000019174 |
| 1 | ! | | 0.0114 | | 12 | | 328 | | 4 | | 0.012 | | 0.333 | | GO:0019825 | | MF | | 9 | | oxygen binding | | 1 | | ENSOARG00000019130,ENSOARG00000019144,ENSOARG00000019163,ENSOARG00000019174 |
| 1 | ! | | 0.023 | | 15 | | 328 | | 4 | | 0.012 | | 0.267 | | GO:0140104 | | MF | | 8 | | molecular carrier activity | | 1 | | ENSOARG00000019130,ENSOARG00000019144,ENSOARG00000019163,ENSOARG00000019174 |
| 1 | ! | | 0.0102 | | 11 | | 328 | | 4 | | 0.012 | | 0.364 | | GO:0005344 | | MF | | 8 | | oxygen carrier activity | | 1 | | ENSOARG00000019130,ENSOARG00000019144,ENSOARG00000019163,ENSOARG00000019174 |
| 1 | ! | | 0.0102 | | 1530 | | 328 | | 50 | | 0.152 | | 0.033 | | GO:0060089 | | MF | | 6 | | molecular transducer activity | | 1 | | ENSOARG00000000204,ENSOARG00000000238,ENSOARG00000000325,ENSOARG00000000603,ENSOARG00000001126,ENSOARG00000001342,ENSOARG00000001408,ENSOARG00000001517,ENSOARG00000003137,ENSOARG00000003825,ENSOARG00000004069,ENSOARG00000004080,ENSOARG00000004093,ENSOARG00000004105,ENSOARG00000004732,ENSOARG00000004748,ENSOARG00000006182,ENSOARG00000006204,ENSOARG00000006696,ENSOARG00000007140,ENSOARG00000007147,ENSOARG00000007336,ENSOARG00000007355,ENSOARG00000007372,ENSOARG00000007386,ENSOARG00000007531,ENSOARG00000007569,ENSOARG00000007583,ENSOARG00000008979,ENSOARG00000011621,ENSOARG00000012328,ENSOARG00000013529,ENSOARG00000013620,ENSOARG00000014001,ENSOARG00000014188,ENSOARG00000014972,ENSOARG00000015457,ENSOARG00000015761,ENSOARG00000016384,ENSOARG00000016502,ENSOARG00000017215,ENSOARG00000017228,ENSOARG00000018241,ENSOARG00000018287,ENSOARG00000018363,ENSOARG00000018380,ENSOARG00000018809,ENSOARG00000019176,ENSOARG00000019747,ENSOARG00000020442 |
| 1 | ! | | 0.0128 | | 1512 | | 328 | | 49 | | 0.149 | | 0.032 | | GO:0004872 | | MF | | 6 | | receptor activity | | 2 | | ENSOARG00000000204,ENSOARG00000000238,ENSOARG00000000325,ENSOARG00000000603,ENSOARG00000001126,ENSOARG00000001342,ENSOARG00000001408,ENSOARG00000001517,ENSOARG00000003137,ENSOARG00000003825,ENSOARG00000004069,ENSOARG00000004080,ENSOARG00000004093,ENSOARG00000004105,ENSOARG00000004732,ENSOARG00000004748,ENSOARG00000006182,ENSOARG00000006204,ENSOARG00000006696,ENSOARG00000007140,ENSOARG00000007147,ENSOARG00000007336,ENSOARG00000007355,ENSOARG00000007372,ENSOARG00000007386,ENSOARG00000007531,ENSOARG00000007569,ENSOARG00000007583,ENSOARG00000008979,ENSOARG00000011621,ENSOARG00000012328,ENSOARG00000013529,ENSOARG00000013620,ENSOARG00000014001,ENSOARG00000014188,ENSOARG00000014972,ENSOARG00000015457,ENSOARG00000015761,ENSOARG00000016502,ENSOARG00000017215,ENSOARG00000017228,ENSOARG00000018241,ENSOARG00000018287,ENSOARG00000018363,ENSOARG00000018380,ENSOARG00000018809,ENSOARG00000019176,ENSOARG00000019747,ENSOARG00000020442 |
| 1 | ! | | 0.0362 | | 1301 | | 328 | | 42 | | 0.128 | | 0.032 | | GO:0099600 | | MF | | 6 | | transmembrane receptor activity | | 3 | | ENSOARG00000000204,ENSOARG00000000238,ENSOARG00000000325,ENSOARG00000000603,ENSOARG00000001126,ENSOARG00000001342,ENSOARG00000001408,ENSOARG00000003137,ENSOARG00000003825,ENSOARG00000004069,ENSOARG00000004080,ENSOARG00000004093,ENSOARG00000004105,ENSOARG00000004732,ENSOARG00000004748,ENSOARG00000006182,ENSOARG00000006204,ENSOARG00000006696,ENSOARG00000007147,ENSOARG00000007336,ENSOARG00000007355,ENSOARG00000007372,ENSOARG00000007386,ENSOARG00000007531,ENSOARG00000007569,ENSOARG00000007583,ENSOARG00000008979,ENSOARG00000011621,ENSOARG00000013529,ENSOARG00000013620,ENSOARG00000014001,ENSOARG00000015761,ENSOARG00000016502,ENSOARG00000017215,ENSOARG00000017228,ENSOARG00000018241,ENSOARG00000018287,ENSOARG00000018363,ENSOARG00000018380,ENSOARG00000018809,ENSOARG00000019176,ENSOARG00000019747 |
| 1 | ! | | 0.0372 | | 1347 | | 328 | | 43 | | 0.131 | | 0.032 | | GO:0038023 | | MF | | 6 | | signaling receptor activity | | 3 | | ENSOARG00000000204,ENSOARG00000000238,ENSOARG00000000325,ENSOARG00000000603,ENSOARG00000001126,ENSOARG00000001342,ENSOARG00000001408,ENSOARG00000003137,ENSOARG00000003825,ENSOARG00000004069,ENSOARG00000004080,ENSOARG00000004093,ENSOARG00000004105,ENSOARG00000004732,ENSOARG00000004748,ENSOARG00000006182,ENSOARG00000006204,ENSOARG00000006696,ENSOARG00000007140,ENSOARG00000007147,ENSOARG00000007336,ENSOARG00000007355,ENSOARG00000007372,ENSOARG00000007386,ENSOARG00000007531,ENSOARG00000007569,ENSOARG00000007583,ENSOARG00000008979,ENSOARG00000011621,ENSOARG00000013529,ENSOARG00000013620,ENSOARG00000014001,ENSOARG00000015761,ENSOARG00000016502,ENSOARG00000017215,ENSOARG00000017228,ENSOARG00000018241,ENSOARG00000018287,ENSOARG00000018363,ENSOARG00000018380,ENSOARG00000018809,ENSOARG00000019176,ENSOARG00000019747 |
| 1 | ! | | 0.023 | | 1269 | | 328 | | 42 | | 0.128 | | 0.033 | | GO:0004888 | | MF | | 6 | | transmembrane signaling receptor activity | | 4 | | ENSOARG00000000204,ENSOARG00000000238,ENSOARG00000000325,ENSOARG00000000603,ENSOARG00000001126,ENSOARG00000001342,ENSOARG00000001408,ENSOARG00000003137,ENSOARG00000003825,ENSOARG00000004069,ENSOARG00000004080,ENSOARG00000004093,ENSOARG00000004105,ENSOARG00000004732,ENSOARG00000004748,ENSOARG00000006182,ENSOARG00000006204,ENSOARG00000006696,ENSOARG00000007147,ENSOARG00000007336,ENSOARG00000007355,ENSOARG00000007372,ENSOARG00000007386,ENSOARG00000007531,ENSOARG00000007569,ENSOARG00000007583,ENSOARG00000008979,ENSOARG00000011621,ENSOARG00000013529,ENSOARG00000013620,ENSOARG00000014001,ENSOARG00000015761,ENSOARG00000016502,ENSOARG00000017215,ENSOARG00000017228,ENSOARG00000018241,ENSOARG00000018287,ENSOARG00000018363,ENSOARG00000018380,ENSOARG00000018809,ENSOARG00000019176,ENSOARG00000019747 |
| 1 | ! | | 0.0017 | | 592 | | 328 | | 29 | | 0.088 | | 0.049 | | GO:0004984 | | MF | | 6 | | olfactory receptor activity | | 5 | | ENSOARG00000000204,ENSOARG00000000238,ENSOARG00000000325,ENSOARG00000001126,ENSOARG00000001342,ENSOARG00000003137,ENSOARG00000004069,ENSOARG00000004080,ENSOARG00000004093,ENSOARG00000004105,ENSOARG00000004748,ENSOARG00000006182,ENSOARG00000006204,ENSOARG00000006696,ENSOARG00000007336,ENSOARG00000007355,ENSOARG00000007372,ENSOARG00000007386,ENSOARG00000007531,ENSOARG00000007569,ENSOARG00000007583,ENSOARG00000008979,ENSOARG00000011621,ENSOARG00000017215,ENSOARG00000017228,ENSOARG00000018287,ENSOARG00000018363,ENSOARG00000018380,ENSOARG00000019176 |
| 1 | ! | | 0.0104 | | 969 | | 328 | | 36 | | 0.11 | | 0.037 | | GO:0004930 | | MF | | 6 | | G-protein coupled receptor activity | | 5 | | ENSOARG00000000204,ENSOARG00000000238,ENSOARG00000000325,ENSOARG00000001126,ENSOARG00000001342,ENSOARG00000003137,ENSOARG00000003825,ENSOARG00000004069,ENSOARG00000004080,ENSOARG00000004093,ENSOARG00000004105,ENSOARG00000004748,ENSOARG00000006182,ENSOARG00000006204,ENSOARG00000006696,ENSOARG00000007147,ENSOARG00000007336,ENSOARG00000007355,ENSOARG00000007372,ENSOARG00000007386,ENSOARG00000007531,ENSOARG00000007569,ENSOARG00000007583,ENSOARG00000008979,ENSOARG00000011621,ENSOARG00000013529,ENSOARG00000013620,ENSOARG00000015761,ENSOARG00000016502,ENSOARG00000017215,ENSOARG00000017228,ENSOARG00000018287,ENSOARG00000018363,ENSOARG00000018380,ENSOARG00000019176,ENSOARG00000019747 |
| 1 | ! | | 0.00497 | | 3 | | 85 | | 3 | | 0.035 | | 1 | | HP:0200023 | | hp | | 16 | | Priapism | | 1 | | ENSOARG00000019144,ENSOARG00000019163,ENSOARG00000019174 |
| 1 | ! | | 0.00497 | | 3 | | 85 | | 3 | | 0.035 | | 1 | | HP:0008346 | | hp | | 18 | | Increased red cell sickling tendency | | 1 | | ENSOARG00000019144,ENSOARG00000019163,ENSOARG00000019174 |
| 1 | ! | | 0.0219 | | 5 | | 85 | | 3 | | 0.035 | | 0.6 | | HP:0012415 | | hp | | 7 | | Abnormal blood gas level | | 1 | | ENSOARG00000019144,ENSOARG00000019163,ENSOARG00000019174 |
| 1 | ! | | 0.0219 | | 5 | | 85 | | 3 | | 0.035 | | 0.6 | | HP:0012418 | | hp | | 7 | | Hypoxemia | | 2 | | ENSOARG00000019144,ENSOARG00000019163,ENSOARG00000019174 |
| 1 | ! | | 0.014 | | 4 | | 85 | | 3 | | 0.035 | | 0.75 | | HP:0005511 | | hp | | 1 | | Heinz body anemia | | 1 | | ENSOARG00000019144,ENSOARG00000019163,ENSOARG00000019174 |
| 1 | ! | | 0.014 | | 4 | | 85 | | 3 | | 0.035 | | 0.75 | | HP:0011981 | | hp | | 15 | | Pigment gallstones | | 1 | | ENSOARG00000019144,ENSOARG00000019163,ENSOARG00000019174 |
| 1 | ! | | 0.00497 | | 7 | | 85 | | 4 | | 0.047 | | 0.571 | | HP:0025409 | | hp | | 19 | | Abnormal spleen physiology | | 1 | | ENSOARG00000001517,ENSOARG00000019144,ENSOARG00000019163,ENSOARG00000019174 |
| 1 | ! | | 0.00497 | | 7 | | 85 | | 4 | | 0.047 | | 0.571 | | HP:0001971 | | hp | | 19 | | Hypersplenism | | 2 | | ENSOARG00000001517,ENSOARG00000019144,ENSOARG00000019163,ENSOARG00000019174 |
| 1 | ! | | 0.0219 | | 5 | | 85 | | 3 | | 0.035 | | 0.6 | | HP:0005560 | | hp | | 12 | | Imbalanced hemoglobin synthesis | | 1 | | ENSOARG00000019144,ENSOARG00000019163,ENSOARG00000019174 |
| 1 | ! | | 0.00497 | | 3 | | 85 | | 3 | | 0.035 | | 1 | | HP:0011906 | | hp | | 12 | | Reduced beta/alpha synthesis ratio | | 2 | | ENSOARG00000019144,ENSOARG00000019163,ENSOARG00000019174 |
| 1 | ! | | 0.0219 | | 5 | | 85 | | 3 | | 0.035 | | 0.6 | | HP:0011907 | | hp | | 12 | | Reduced alpha/beta synthesis ratio | | 2 | | ENSOARG00000019144,ENSOARG00000019163,ENSOARG00000019174 |
| 1 | ! | | 0.0357 | | 14 | | 85 | | 4 | | 0.047 | | 0.286 | | HP:0012100 | | hp | | 4 | | Abnormal circulating creatinine level | | 1 | | ENSOARG00000010887,ENSOARG00000019144,ENSOARG00000019163,ENSOARG00000019174 |
| 1 | ! | | 0.0281 | | 13 | | 85 | | 4 | | 0.047 | | 0.308 | | HP:0003259 | | hp | | 4 | | Elevated serum creatinine | | 1 | | ENSOARG00000010887,ENSOARG00000019144,ENSOARG00000019163,ENSOARG00000019174 |
| #INFO: | PARAMETERS: fdr = 1 | | | |  | |  | |  | |  | |  | |  | |  | |  | |  | |  | |  |
| #INFO: | PARAMETERS: sort_by_structure = 1 | | | |  | |  | |  | |  | |  | |  | |  | |  | |  | |  | |  |
| #INFO: | PARAMETERS: significant = 1 | | | |  | |  | |  | |  | |  | |  | |  | |  | |  | |  | |  |
| #INFO: | PARAMETERS: organism = oaries | | | |  | |  | |  | |  | |  | |  | |  | |  | |  | |  | |  |
| #INFO: | PARAMETERS: user_thr = 1.00 | | | |  | |  | |  | |  | |  | |  | |  | |  | |  | |  | |  |
| #INFO: | User: http | | | |  | |  | |  | |  | |  | |  | |  | |  | |  | |  | |  |
| #INFO: | Host: arak-prod | | | |  | |  | |  | |  | |  | |  | |  | |  | |  | |  | |  |
| #INFO: | Time: 2017-12-12 12:57:0 | | | |  | |  | |  | |  | |  | |  | |  | |  | |  | |  | |  |
| #INFO: | Version: r1741_e90_eg37 | | | |  | |  | |  | |  | |  | |  | |  | |  | |  | |  | |  |
| #INFO: | Effective domain size for GO: 17974, threshold 0.05 | | | |  | |  | |  | |  | |  | |  | |  | |  | |  | |  | |  |
| #INFO: | Effective domain size for HP: 3692, threshold 0.05 | | | |  | |  | |  | |  | |  | |  | |  | |  | |  | |  | |  |
| #INFO: |  | |  | |  | |  | |  | |  | |  | |  | |  | |  | |  | |  | |  |
| #INFO: | --- Gene names and descriptions | | | |  | |  | |  | |  | |  | |  | |  | |  | |  | |  | |  |
| #INFO: |  | |  | |  | |  | |  | |  | |  | |  | |  | |  | |  | |  | |  |
| #INFO: | QUERY: | | ENSOARG00000000084 | | BSX | | brain specific homeobox [Source:HGNC Symbol;Acc:HGNC:20450] | | | | | | | | | | | | | |  | |  | |  |
| #INFO: | QUERY: | | ENSOARG00000000106 | | VRK1 | | vaccinia related kinase 1 [Source:HGNC Symbol;Acc:HGNC:12718] | | | | | | | | | | | | | |  | |  | |  |
| #INFO: | QUERY: | | ENSOARG00000000193 | | CADPS2 | | calcium dependent secretion activator 2 [Source:HGNC Symbol;Acc:HGNC:16018] | | | | | | | | | | | | | | | |  | |  |
| #INFO: | QUERY: | | ENSOARG00000000204 | | N/A | | N/A | |  | |  | |  | |  | |  | |  | |  | |  | |  |
| #INFO: | QUERY: | ENSOARG00000000238 | | N/A | | N/A | |  | |  | |  | |  | |  | |  | |  | |  | |  | |
| #INFO: | QUERY: | ENSOARG00000000254 | | N/A | | N/A | |  | |  | |  | |  | |  | |  | |  | |  | |  | |
| #INFO: | QUERY: | ENSOARG00000000290 | | N/A | | N/A | |  | |  | |  | |  | |  | |  | |  | |  | |  | |
| #INFO: | QUERY: | | ENSOARG00000000325 | | N/A | | N/A | |  | |  | |  | |  | |  | |  | |  | |  | |  |
| #INFO: | QUERY: | | ENSOARG00000000387 | | RNF148 | | ring finger protein 148 [Source:HGNC Symbol;Acc:HGNC:22411] | | | | | | | | | | | | | |  | |  | |  |
| #INFO: | QUERY: | | ENSOARG00000000407 | | N/A | | N/A | |  | |  | |  | |  | |  | |  | |  | |  | |  |
| #INFO: | QUERY: | | ENSOARG00000000443 | | N/A | | N/A | |  | |  | |  | |  | |  | |  | |  | |  | |  |
| #INFO: | QUERY: | | ENSOARG00000000492 | | NLRP12 | | NLR family pyrin domain containing 12 [Source:HGNC Symbol;Acc:HGNC:22938] | | | | | | | | | | | | | | | |  | |  |
| #INFO: | QUERY: | | ENSOARG00000000603 | | FLT4 | | fms related tyrosine kinase 4 [Source:HGNC Symbol;Acc:HGNC:3767] | | | | | | | | | | | | | |  | |  | |  |
| #INFO: | QUERY: | | ENSOARG00000000681 | | SCN3B | | sodium voltage-gated channel beta subunit 3 [Source:HGNC Symbol;Acc:HGNC:20665] | | | | | | | | | | | | | | | |  | |  |
| #INFO: | QUERY: | | ENSOARG00000000770 | | ZNF202 | | zinc finger protein 202 [Source:HGNC Symbol;Acc:HGNC:12994] | | | | | | | | | | | | | |  | |  | |  |
| #INFO: | QUERY: | | ENSOARG00000000773 | | ASB15 | | ankyrin repeat and SOCS box containing 15 [Source:HGNC Symbol;Acc:HGNC:19767] | | | | | | | | | | | | | | | |  | |  |
| #INFO: | QUERY: | | ENSOARG00000001024 | | N/A | | N/A | |  | |  | |  | |  | |  | |  | |  | |  | |  |
| #INFO: | QUERY: | | ENSOARG00000001052 | | N/A | | N/A | |  | |  | |  | |  | |  | |  | |  | |  | |  |
| #INFO: | QUERY: | | ENSOARG00000001118 | | RNF133 | | ring finger protein 133 [Source:HGNC Symbol;Acc:HGNC:21154] | | | | | | | | | | | | | |  | |  | |  |
| #INFO: | QUERY: | | ENSOARG00000001122 | | HYAL4 | | hyaluronoglucosaminidase 4 [Source:HGNC Symbol;Acc:HGNC:5323] | | | | | | | | | | | | | |  | |  | |  |
| #INFO: | QUERY: | | ENSOARG00000001126 | | N/A | | N/A | |  | |  | |  | |  | |  | |  | |  | |  | |  |
| #INFO: | QUERY: | | ENSOARG00000001129 | | N/A | | N/A | |  | |  | |  | |  | |  | |  | |  | |  | |  |
| #INFO: | QUERY: | | ENSOARG00000001193 | | N/A | | N/A | |  | |  | |  | |  | |  | |  | |  | |  | |  |
| #INFO: | QUERY: | | ENSOARG00000001300 | | XYLB | | xylulokinase [Source:HGNC Symbol;Acc:HGNC:12839] | | | | | | | | | | | |  | |  | |  | |  |
| #INFO: | QUERY: | | ENSOARG00000001301 | | RRM1 | | ribonucleotide reductase catalytic subunit M1 [Source:HGNC Symbol;Acc:HGNC:10451] | | | | | | | | | | | | | | | |  | |  |
| #INFO: | QUERY: | | ENSOARG00000001342 | | OR2A5 | | olfactory receptor family 2 subfamily A member 5 [Source:HGNC Symbol;Acc:HGNC:8232] | | | | | | | | | | | | | | | |  | |  |
| #INFO: | QUERY: | | ENSOARG00000001408 | | ACVR2B | | activin A receptor type 2B [Source:HGNC Symbol;Acc:HGNC:174] | | | | | | | | | | | | | |  | |  | |  |
| #INFO: | QUERY: | | ENSOARG00000001443 | | FSTL5 | | follistatin like 5 [Source:HGNC Symbol;Acc:HGNC:21386] | | | | | | | | | | | |  | |  | |  | |  |
| #INFO: | QUERY: | | ENSOARG00000001451 | | N/A | | N/A | |  | |  | |  | |  | |  | |  | |  | |  | |  |
| #INFO: | QUERY: | | ENSOARG00000001455 | | EXOG | | exo/endonuclease G [Source:HGNC Symbol;Acc:HGNC:3347] | | | | | | | | | | | |  | |  | |  | |  |
| #INFO: | QUERY: | | ENSOARG00000001517 | | N/A | | N/A | |  | |  | |  | |  | |  | |  | |  | |  | |  |
| #INFO: | QUERY: | | ENSOARG00000001527 | | POT1 | | protection of telomeres 1 [Source:HGNC Symbol;Acc:HGNC:17284] | | | | | | | | | | | | | |  | |  | |  |
| #INFO: | QUERY: | | ENSOARG00000001653 | | MAPK9 | | mitogen-activated protein kinase 9 [Source:HGNC Symbol;Acc:HGNC:6886] | | | | | | | | | | | | | | | |  | |  |
| #INFO: | QUERY: | | ENSOARG00000001754 | | MDFIC | | MyoD family inhibitor domain containing [Source:HGNC Symbol;Acc:HGNC:28870] | | | | | | | | | | | | | | | |  | |  |
| #INFO: | QUERY: | | ENSOARG00000001843 | | GAP43 | | growth associated protein 43 [Source:HGNC Symbol;Acc:HGNC:4140] | | | | | | | | | | | | | |  | |  | |  |
| #INFO: | QUERY: | | ENSOARG00000001856 | | N/A | | N/A | |  | |  | |  | |  | |  | |  | |  | |  | |  |
| #INFO: | QUERY: | | ENSOARG00000001935 | | HEPHL1 | | hephaestin like 1 [Source:HGNC Symbol;Acc:HGNC:30477] | | | | | | | | | | | |  | |  | |  | |  |
| #INFO: | QUERY: | | ENSOARG00000001974 | | RNF130 | | ring finger protein 130 [Source:HGNC Symbol;Acc:HGNC:18280] | | | | | | | | | | | | | |  | |  | |  |
| #INFO: | QUERY: | | ENSOARG00000002115 | | MRE11 | | MRE11 homolog, double strand break repair nuclease [Source:HGNC Symbol;Acc:HGNC:7230] | | | | | | | | | | | | | | | |  | |  |
| #INFO: | QUERY: | | ENSOARG00000002168 | | OTOG | | otogelin [Source:HGNC Symbol;Acc:HGNC:8516] | | | | | | | | | |  | |  | |  | |  | |  |
| #INFO: | QUERY: | | ENSOARG00000002329 | | GAD1 | | glutamate decarboxylase 1 [Source:HGNC Symbol;Acc:HGNC:4092] | | | | | | | | | | | | | |  | |  | |  |
| #INFO: | QUERY: | | ENSOARG00000002452 | | SYNRG | | synergin gamma [Source:HGNC Symbol;Acc:HGNC:557] | | | | | | | | | | | |  | |  | |  | |  |
| #INFO: | QUERY: | | ENSOARG00000002530 | | ANKRD49 | | ankyrin repeat domain 49 [Source:HGNC Symbol;Acc:HGNC:25970] | | | | | | | | | | | | | |  | |  | |  |
| #INFO: | QUERY: | | ENSOARG00000002602 | | AASDHPPT | | aminoadipate-semialdehyde dehydrogenase-phosphopantetheinyl transferase [Source:HGNC Symbol;Acc:HGNC:14235] | | | | | | | | | | | | | | | | | | |
| #INFO: | QUERY: | | ENSOARG00000002656 | | N/A | | N/A | |  | |  | |  | |  | |  | |  | |  | |  | |  |
| #INFO: | QUERY: | | ENSOARG00000002702 | | N/A | | N/A | |  | |  | |  | |  | |  | |  | |  | |  | |  |
| #INFO: | QUERY: | | ENSOARG00000002739 | | N/A | | N/A | |  | |  | |  | |  | |  | |  | |  | |  | |  |
| #INFO: | QUERY: | | ENSOARG00000002984 | | CFAP52 | | cilia and flagella associated protein 52 [Source:HGNC Symbol;Acc:HGNC:16053] | | | | | | | | | | | | | | | |  | |  |
| #INFO: | QUERY: | | ENSOARG00000002991 | | CXCL12 | | C-X-C motif chemokine ligand 12 [Source:HGNC Symbol;Acc:HGNC:10672] | | | | | | | | | | | | | | | |  | |  |
| #INFO: | QUERY: | | ENSOARG00000002998 | | DDX52 | | DExD-box helicase 52 [Source:HGNC Symbol;Acc:HGNC:20038] | | | | | | | | | | | |  | |  | |  | |  |
| #INFO: | QUERY: | | ENSOARG00000003031 | | N/A | | N/A | |  | |  | |  | |  | |  | |  | |  | |  | |  |
| #INFO: | QUERY: | | ENSOARG00000003068 | | CASP4 | | caspase 4 [Source:HGNC Symbol;Acc:HGNC:1505] | | | | | | | | | |  | |  | |  | |  | |  |
| #INFO: | QUERY: | | ENSOARG00000003137 | | OR13C3 | | olfactory receptor family 13 subfamily C member 3 [Source:HGNC Symbol;Acc:HGNC:14704] | | | | | | | | | | | | | | | |  | |  |
| #INFO: | QUERY: | | ENSOARG00000003140 | | USP43 | | ubiquitin specific peptidase 43 [Source:HGNC Symbol;Acc:HGNC:20072] | | | | | | | | | | | | | |  | |  | |  |
| #INFO: | QUERY: | | ENSOARG00000003250 | | SCGN | | secretagogin, EF-hand calcium binding protein [Source:HGNC Symbol;Acc:HGNC:16941] | | | | | | | | | | | | | | | |  | |  |
| #INFO: | QUERY: | | ENSOARG00000003281 | | N/A | | N/A | |  | |  | |  | |  | |  | |  | |  | |  | |  |
| #INFO: | QUERY: | | ENSOARG00000003293 | | PDGFD | | platelet derived growth factor D [Source:HGNC Symbol;Acc:HGNC:30620] | | | | | | | | | | | | | | | |  | |  |
| #INFO: | QUERY: | | ENSOARG00000003329 | | SULF1 | | sulfatase 1 [Source:HGNC Symbol;Acc:HGNC:20391] | | | | | | | | | |  | |  | |  | |  | |  |
| #INFO: | QUERY: | | ENSOARG00000003431 | | N/A | | N/A | |  | |  | |  | |  | |  | |  | |  | |  | |  |
| #INFO: | QUERY: | | ENSOARG00000003461 | | N/A | | N/A | |  | |  | |  | |  | |  | |  | |  | |  | |  |
| #INFO: | QUERY: | | ENSOARG00000003497 | | N/A | | N/A | |  | |  | |  | |  | |  | |  | |  | |  | |  |
| #INFO: | QUERY: | | ENSOARG00000003572 | | RPS6KA5 | | ribosomal protein S6 kinase A5 [Source:HGNC Symbol;Acc:HGNC:10434] | | | | | | | | | | | | | |  | |  | |  |
| #INFO: | QUERY: | | ENSOARG00000003590 | | C2ORF73 | | chromosome 2 open reading frame 73 [Source:HGNC Symbol;Acc:HGNC:26861] | | | | | | | | | | | | | | | |  | |  |
| #INFO: | QUERY: | | ENSOARG00000003600 | | N/A | | N/A | |  | |  | |  | |  | |  | |  | |  | |  | |  |
| #INFO: | QUERY: | | ENSOARG00000003805 | | N/A | | N/A | |  | |  | |  | |  | |  | |  | |  | |  | |  |
| #INFO: | QUERY: | | ENSOARG00000003825 | | GPR158 | | G protein-coupled receptor 158 [Source:HGNC Symbol;Acc:HGNC:23689] | | | | | | | | | | | | | |  | |  | |  |
| #INFO: | QUERY: | | ENSOARG00000003926 | | CARMIL1 | | capping protein regulator and myosin 1 linker 1 [Source:HGNC Symbol;Acc:HGNC:21581] | | | | | | | | | | | | | | | |  | |  |
| #INFO: | QUERY: | | ENSOARG00000003933 | | CSGALNACT1 | | chondroitin sulfate N-acetylgalactosaminyltransferase 1 [Source:HGNC Symbol;Acc:HGNC:24290] | | | | | | | | | | | | | | | |  | |  |
| #INFO: | QUERY: | | ENSOARG00000003976 | | SH2D4A | | SH2 domain containing 4A [Source:HGNC Symbol;Acc:HGNC:26102] | | | | | | | | | | | | | |  | |  | |  |
| #INFO: | QUERY: | | ENSOARG00000004018 | | SPINK5 | | serine peptidase inhibitor, Kazal type 5 [Source:HGNC Symbol;Acc:HGNC:15464] | | | | | | | | | | | | | | | |  | |  |
| #INFO: | QUERY: | | ENSOARG00000004069 | | OR1G1 | | olfactory receptor family 1 subfamily G member 1 [Source:HGNC Symbol;Acc:HGNC:8204] | | | | | | | | | | | | | | | |  | |  |
| #INFO: | QUERY: | | ENSOARG00000004080 | | N/A | | N/A | |  | |  | |  | |  | |  | |  | |  | |  | |  |
| #INFO: | QUERY: | | ENSOARG00000004093 | | N/A | | N/A | |  | |  | |  | |  | |  | |  | |  | |  | |  |
| #INFO: | QUERY: | | ENSOARG00000004105 | | N/A | | N/A | |  | |  | |  | |  | |  | |  | |  | |  | |  |
| #INFO: | QUERY: | | ENSOARG00000004107 | | N/A | | N/A | |  | |  | |  | |  | |  | |  | |  | |  | |  |
| #INFO: | QUERY: | | ENSOARG00000004355 | | GATB | | glutamyl-tRNA amidotransferase subunit B [Source:HGNC Symbol;Acc:HGNC:8849] | | | | | | | | | | | | | | | |  | |  |
| #INFO: | QUERY: | | ENSOARG00000004630 | | SEC14L5 | | SEC14 like lipid binding 5 [Source:HGNC Symbol;Acc:HGNC:29032] | | | | | | | | | | | | | |  | |  | |  |
| #INFO: | QUERY: | | ENSOARG00000004634 | | B3GNT4 | | UDP-GlcNAc:betaGal beta-1,3-N-acetylglucosaminyltransferase 4 [Source:HGNC Symbol;Acc:HGNC:15683] | | | | | | | | | | | | | | | | | |  |
| #INFO: | QUERY: | | ENSOARG00000004647 | | ZNF385D | | zinc finger protein 385D [Source:HGNC Symbol;Acc:HGNC:26191] | | | | | | | | | | | | | |  | |  | |  |
| #INFO: | QUERY: | | ENSOARG00000004731 | | N/A | | N/A | |  | |  | |  | |  | |  | |  | |  | |  | |  |
| #INFO: | QUERY: | | ENSOARG00000004732 | | DCC | | DCC netrin 1 receptor [Source:HGNC Symbol;Acc:HGNC:2701] | | | | | | | | | | | |  | |  | |  | |  |
| #INFO: | QUERY: | | ENSOARG00000004748 | | N/A | | N/A | |  | |  | |  | |  | |  | |  | |  | |  | |  |
| #INFO: | QUERY: | | ENSOARG00000004788 | | N/A | | N/A | |  | |  | |  | |  | |  | |  | |  | |  | |  |
| #INFO: | QUERY: | | ENSOARG00000004839 | | NAGPA | | N-acetylglucosamine-1-phosphodiester alpha-N-acetylglucosaminidase [Source:HGNC Symbol;Acc:HGNC:17378] | | | | | | | | | | | | | | | | | | |
| #INFO: | QUERY: | | ENSOARG00000004922 | | C16ORF89 | | chromosome 16 open reading frame 89 [Source:HGNC Symbol;Acc:HGNC:28687] | | | | | | | | | | | | | | | |  | |  |
| #INFO: | QUERY: | | ENSOARG00000005002 | | N/A | | N/A | |  | |  | |  | |  | |  | |  | |  | |  | |  |
| #INFO: | QUERY: | | ENSOARG00000005004 | | FCHO2 | | FCH domain only 2 [Source:HGNC Symbol;Acc:HGNC:25180] | | | | | | | | | | | |  | |  | |  | |  |
| #INFO: | QUERY: | | ENSOARG00000005045 | | N/A | | N/A | |  | |  | |  | |  | |  | |  | |  | |  | |  |
| #INFO: | QUERY: | | ENSOARG00000005096 | | N/A | | N/A | |  | |  | |  | |  | |  | |  | |  | |  | |  |
| #INFO: | QUERY: | | ENSOARG00000005115 | | METTL18 | | methyltransferase like 18 [Source:HGNC Symbol;Acc:HGNC:28793] | | | | | | | | | | | | | |  | |  | |  |
| #INFO: | QUERY: | | ENSOARG00000005149 | | KCNIP4 | | potassium voltage-gated channel interacting protein 4 [Source:HGNC Symbol;Acc:HGNC:30083] | | | | | | | | | | | | | | | |  | |  |
| #INFO: | QUERY: | | ENSOARG00000005168 | | SYT4 | | synaptotagmin 4 [Source:HGNC Symbol;Acc:HGNC:11512] | | | | | | | | | | | |  | |  | |  | |  |
| #INFO: | QUERY: | | ENSOARG00000005235 | | GYG1 | | glycogenin 1 [Source:HGNC Symbol;Acc:HGNC:4699] | | | | | | | | | | | |  | |  | |  | |  |
| #INFO: | QUERY: | | ENSOARG00000005368 | | SLC10A2 | | solute carrier family 10 member 2 [Source:HGNC Symbol;Acc:HGNC:10906] | | | | | | | | | | | | | | | |  | |  |
| #INFO: | QUERY: | | ENSOARG00000005371 | | ST3GAL1 | | ST3 beta-galactoside alpha-2,3-sialyltransferase 1 [Source:HGNC Symbol;Acc:HGNC:10862] | | | | | | | | | | | | | | | |  | |  |
| #INFO: | QUERY: | | ENSOARG00000005430 | | N/A | | N/A | |  | |  | |  | |  | |  | |  | |  | |  | |  |
| #INFO: | QUERY: | | ENSOARG00000005437 | | N/A | | N/A | |  | |  | |  | |  | |  | |  | |  | |  | |  |
| #INFO: | QUERY: | | ENSOARG00000005461 | | KBTBD12 | | kelch repeat and BTB domain containing 12 [Source:HGNC Symbol;Acc:HGNC:25731] | | | | | | | | | | | | | | | |  | |  |
| #INFO: | QUERY: | | ENSOARG00000005603 | | KHDRBS2 | | KH RNA binding domain containing, signal transduction associated 2 [Source:HGNC Symbol;Acc:HGNC:18114] | | | | | | | | | | | | | | | | | |  |
| #INFO: | QUERY: | | ENSOARG00000005617 | | TNFSF13B | | TNF superfamily member 13b [Source:HGNC Symbol;Acc:HGNC:11929] | | | | | | | | | | | | | |  | |  | |  |
| #INFO: | QUERY: | | ENSOARG00000005785 | | MYO16 | | myosin XVI [Source:HGNC Symbol;Acc:HGNC:29822] | | | | | | | | | | | |  | |  | |  | |  |
| #INFO: | QUERY: | | ENSOARG00000006182 | | N/A | | N/A | |  | |  | |  | |  | |  | |  | |  | |  | |  |
| #INFO: | QUERY: | | ENSOARG00000006204 | | OR52E2 | | olfactory receptor family 52 subfamily E member 2 [Source:HGNC Symbol;Acc:HGNC:14769] | | | | | | | | | | | | | | | |  | |  |
| #INFO: | QUERY: | | ENSOARG00000006266 | | TMEM123 | | transmembrane protein 123 [Source:HGNC Symbol;Acc:HGNC:30138] | | | | | | | | | | | | | |  | |  | |  |
| #INFO: | QUERY: | | ENSOARG00000006356 | | BIRC3 | | baculoviral IAP repeat containing 3 [Source:HGNC Symbol;Acc:HGNC:591] | | | | | | | | | | | | | | | |  | |  |
| #INFO: | QUERY: | | ENSOARG00000006515 | | COL4A2 | | collagen type IV alpha 2 chain [Source:HGNC Symbol;Acc:HGNC:2203] | | | | | | | | | | | | | |  | |  | |  |
| #INFO: | QUERY: | | ENSOARG00000006696 | | N/A | | N/A | |  | |  | |  | |  | |  | |  | |  | |  | |  |
| #INFO: | QUERY: | | ENSOARG00000006741 | | INTS4 | | integrator complex subunit 4 [Source:HGNC Symbol;Acc:HGNC:25048] | | | | | | | | | | | | | |  | |  | |  |
| #INFO: | QUERY: | | ENSOARG00000006767 | | N/A | | N/A | |  | |  | |  | |  | |  | |  | |  | |  | |  |
| #INFO: | QUERY: | | ENSOARG00000006806 | | SERPINB11 | | serpin family B member 11 (gene/pseudogene) [Source:HGNC Symbol;Acc:HGNC:14221] | | | | | | | | | | | | | | | |  | |  |
| #INFO: | QUERY: | | ENSOARG00000006824 | | AAMDC | | adipogenesis associated Mth938 domain containing [Source:HGNC Symbol;Acc:HGNC:30205] | | | | | | | | | | | | | | | |  | |  |
| #INFO: | QUERY: | | ENSOARG00000006880 | | SERPINB7 | | serpin family B member 7 [Source:HGNC Symbol;Acc:HGNC:13902] | | | | | | | | | | | | | |  | |  | |  |
| #INFO: | QUERY: | | ENSOARG00000007073 | | ERCC8 | | ERCC excision repair 8, CSA ubiquitin ligase complex subunit [Source:HGNC Symbol;Acc:HGNC:3439] | | | | | | | | | | | | | | | |  | |  |
| #INFO: | QUERY: | | ENSOARG00000007100 | | TGM3 | | transglutaminase 3 [Source:HGNC Symbol;Acc:HGNC:11779] | | | | | | | | | | | |  | |  | |  | |  |
| #INFO: | QUERY: | | ENSOARG00000007140 | | HNF4G | | hepatocyte nuclear factor 4 gamma [Source:HGNC Symbol;Acc:HGNC:5026] | | | | | | | | | | | | | | | |  | |  |
| #INFO: | QUERY: | | ENSOARG00000007147 | | N/A | | N/A | |  | |  | |  | |  | |  | |  | |  | |  | |  |
| #INFO: | QUERY: | | ENSOARG00000007198 | | N/A | | N/A | |  | |  | |  | |  | |  | |  | |  | |  | |  |
| #INFO: | QUERY: | | ENSOARG00000007223 | | TRIM7 | | tripartite motif containing 7 [Source:HGNC Symbol;Acc:HGNC:16278] | | | | | | | | | | | | | |  | |  | |  |
| #INFO: | QUERY: | | ENSOARG00000007336 | | N/A | | N/A | |  | |  | |  | |  | |  | |  | |  | |  | |  |
| #INFO: | QUERY: | | ENSOARG00000007355 | | N/A | | N/A | |  | |  | |  | |  | |  | |  | |  | |  | |  |
| #INFO: | QUERY: | | ENSOARG00000007372 | | OR51V1 | | olfactory receptor family 51 subfamily V member 1 [Source:HGNC Symbol;Acc:HGNC:19597] | | | | | | | | | | | | | | | |  | |  |
| #INFO: | QUERY: | | ENSOARG00000007386 | | OR52Z1 | | olfactory receptor family 52 subfamily Z member 1 (gene/pseudogene) [Source:HGNC Symbol;Acc:HGNC:19596] | | | | | | | | | | | | | | | | | | |
| #INFO: | QUERY: | | ENSOARG00000007484 | | WDSUB1 | | WD repeat, sterile alpha motif and U-box domain containing 1 [Source:HGNC Symbol;Acc:HGNC:26697] | | | | | | | | | | | | | | | | | |  |
| #INFO: | QUERY: | | ENSOARG00000007488 | | SPTA1 | | spectrin alpha, erythrocytic 1 [Source:HGNC Symbol;Acc:HGNC:11272] | | | | | | | | | | | | | |  | |  | |  |
| #INFO: | QUERY: | | ENSOARG00000007531 | | OR6K2 | | olfactory receptor family 6 subfamily K member 2 [Source:HGNC Symbol;Acc:HGNC:15029] | | | | | | | | | | | | | | | |  | |  |
| #INFO: | QUERY: | | ENSOARG00000007560 | | ARHGAP42 | | Rho GTPase activating protein 42 [Source:HGNC Symbol;Acc:HGNC:26545] | | | | | | | | | | | | | | | |  | |  |
| #INFO: | QUERY: | | ENSOARG00000007569 | | N/A | | N/A | |  | |  | |  | |  | |  | |  | |  | |  | |  |
| #INFO: | QUERY: | | ENSOARG00000007583 | | N/A | | N/A | |  | |  | |  | |  | |  | |  | |  | |  | |  |
| #INFO: | QUERY: | | ENSOARG00000007585 | | TANC1 | | tetratricopeptide repeat, ankyrin repeat and coiled-coil containing 1 [Source:HGNC Symbol;Acc:HGNC:29364] | | | | | | | | | | | | | | | | | |  |
| #INFO: | QUERY: | | ENSOARG00000007645 | | N/A | | N/A | |  | |  | |  | |  | |  | |  | |  | |  | |  |
| #INFO: | QUERY: | | ENSOARG00000007652 | | N/A | | N/A | |  | |  | |  | |  | |  | |  | |  | |  | |  |
| #INFO: | QUERY: | | ENSOARG00000007657 | | CDK20 | | cyclin dependent kinase 20 [Source:HGNC Symbol;Acc:HGNC:21420] | | | | | | | | | | | | | |  | |  | |  |
| #INFO: | QUERY: | | ENSOARG00000007693 | | NUDT7 | | nudix hydrolase 7 [Source:HGNC Symbol;Acc:HGNC:8054] | | | | | | | | | | | |  | |  | |  | |  |
| #INFO: | QUERY: | | ENSOARG00000007705 | | ERICH6B | | glutamate rich 6B [Source:HGNC Symbol;Acc:HGNC:26523] | | | | | | | | | | | |  | |  | |  | |  |
| #INFO: | QUERY: | | ENSOARG00000007736 | | SPERT | | spermatid associated [Source:HGNC Symbol;Acc:HGNC:30720] | | | | | | | | | | | |  | |  | |  | |  |
| #INFO: | QUERY: | | ENSOARG00000007770 | | TMPRSS11D | | transmembrane protease, serine 11D [Source:HGNC Symbol;Acc:HGNC:24059] | | | | | | | | | | | | | | | |  | |  |
| #INFO: | QUERY: | | ENSOARG00000007772 | | N/A | | N/A | |  | |  | |  | |  | |  | |  | |  | |  | |  |
| #INFO: | QUERY: | | ENSOARG00000007791 | | VAT1L | | vesicle amine transport 1 like [Source:HGNC Symbol;Acc:HGNC:29315] | | | | | | | | | | | | | |  | |  | |  |
| #INFO: | QUERY: | | ENSOARG00000007866 | | PSMB9 | | proteasome subunit beta 9 [Source:HGNC Symbol;Acc:HGNC:9546] | | | | | | | | | | | | | |  | |  | |  |
| #INFO: | QUERY: | | ENSOARG00000007892 | | N/A | | N/A | |  | |  | |  | |  | |  | |  | |  | |  | |  |
| #INFO: | QUERY: | | ENSOARG00000007906 | | N/A | | N/A | |  | |  | |  | |  | |  | |  | |  | |  | |  |
| #INFO: | QUERY: | | ENSOARG00000007954 | | FGFBP1 | | fibroblast growth factor binding protein 1 [Source:HGNC Symbol;Acc:HGNC:19695] | | | | | | | | | | | | | | | |  | |  |
| #INFO: | QUERY: | | ENSOARG00000008039 | | N/A | | N/A | |  | |  | |  | |  | |  | |  | |  | |  | |  |
| #INFO: | QUERY: | | ENSOARG00000008224 | | DAB1 | | DAB1, reelin adaptor protein [Source:HGNC Symbol;Acc:HGNC:2661] | | | | | | | | | | | | | |  | |  | |  |
| #INFO: | QUERY: | | ENSOARG00000008380 | | RAB6B | | RAB6B, member RAS oncogene family [Source:HGNC Symbol;Acc:HGNC:14902] | | | | | | | | | | | | | | | |  | |  |
| #INFO: | QUERY: | | ENSOARG00000008408 | | MAN1A1 | | mannosidase alpha class 1A member 1 [Source:HGNC Symbol;Acc:HGNC:6821] | | | | | | | | | | | | | | | |  | |  |
| #INFO: | QUERY: | | ENSOARG00000008412 | | SRPRB | | SRP receptor beta subunit [Source:HGNC Symbol;Acc:HGNC:24085] | | | | | | | | | | | | | |  | |  | |  |
| #INFO: | QUERY: | | ENSOARG00000008514 | | HSD17B3 | | hydroxysteroid 17-beta dehydrogenase 3 [Source:HGNC Symbol;Acc:HGNC:5212] | | | | | | | | | | | | | | | |  | |  |
| #INFO: | QUERY: | | ENSOARG00000008602 | | CRBN | | cereblon [Source:HGNC Symbol;Acc:HGNC:30185] | | | | | | | | | |  | |  | |  | |  | |  |
| #INFO: | QUERY: | | ENSOARG00000008640 | | DHX57 | | DExH-box helicase 57 [Source:HGNC Symbol;Acc:HGNC:20086] | | | | | | | | | | | |  | |  | |  | |  |
| #INFO: | QUERY: | | ENSOARG00000008932 | | ANO2 | | anoctamin 2 [Source:HGNC Symbol;Acc:HGNC:1183] | | | | | | | | | | | |  | |  | |  | |  |
| #INFO: | QUERY: | | ENSOARG00000008959 | | SLIT1 | | slit guidance ligand 1 [Source:HGNC Symbol;Acc:HGNC:11085] | | | | | | | | | | | |  | |  | |  | |  |
| #INFO: | QUERY: | | ENSOARG00000008975 | | MYH11 | | myosin heavy chain 11 [Source:HGNC Symbol;Acc:HGNC:7569] | | | | | | | | | | | |  | |  | |  | |  |
| #INFO: | QUERY: | | ENSOARG00000008979 | | N/A | | N/A | |  | |  | |  | |  | |  | |  | |  | |  | |  |
| #INFO: | QUERY: | | ENSOARG00000009086 | | N/A | | N/A | |  | |  | |  | |  | |  | |  | |  | |  | |  |
| #INFO: | QUERY: | | ENSOARG00000009276 | | EYA2 | | EYA transcriptional coactivator and phosphatase 2 [Source:HGNC Symbol;Acc:HGNC:3520] | | | | | | | | | | | | | | | |  | |  |
| #INFO: | QUERY: | | ENSOARG00000009490 | | N/A | | N/A | |  | |  | |  | |  | |  | |  | |  | |  | |  |
| #INFO: | QUERY: | | ENSOARG00000009536 | | PLCB4 | | phospholipase C beta 4 [Source:HGNC Symbol;Acc:HGNC:9059] | | | | | | | | | | | | | |  | |  | |  |
| #INFO: | QUERY: | | ENSOARG00000009547 | | SHQ1 | | SHQ1, H/ACA ribonucleoprotein assembly factor [Source:HGNC Symbol;Acc:HGNC:25543] | | | | | | | | | | | | | | | |  | |  |
| #INFO: | QUERY: | | ENSOARG00000009656 | | POLR3A | | RNA polymerase III subunit A [Source:HGNC Symbol;Acc:HGNC:30074] | | | | | | | | | | | | | |  | |  | |  |
| #INFO: | QUERY: | | ENSOARG00000009678 | | TUSC3 | | tumor suppressor candidate 3 [Source:HGNC Symbol;Acc:HGNC:30242] | | | | | | | | | | | | | |  | |  | |  |
| #INFO: | QUERY: | | ENSOARG00000009780 | | N/A | | N/A | |  | |  | |  | |  | |  | |  | |  | |  | |  |
| #INFO: | QUERY: | | ENSOARG00000009829 | | FUT4 | | fucosyltransferase 4 [Source:HGNC Symbol;Acc:HGNC:4015] | | | | | | | | | | | |  | |  | |  | |  |
| #INFO: | QUERY: | | ENSOARG00000009854 | | EXT1 | | exostosin glycosyltransferase 1 [Source:HGNC Symbol;Acc:HGNC:3512] | | | | | | | | | | | | | |  | |  | |  |
| #INFO: | QUERY: | | ENSOARG00000009938 | | KYNU | | kynureninase [Source:HGNC Symbol;Acc:HGNC:6469] | | | | | | | | | | | |  | |  | |  | |  |
| #INFO: | QUERY: | | ENSOARG00000009979 | | DSCAM | | DS cell adhesion molecule [Source:HGNC Symbol;Acc:HGNC:3039] | | | | | | | | | | | | | |  | |  | |  |
| #INFO: | QUERY: | | ENSOARG00000010131 | | FGF6 | | fibroblast growth factor 6 [Source:HGNC Symbol;Acc:HGNC:3684] | | | | | | | | | | | | | |  | |  | |  |
| #INFO: | QUERY: | | ENSOARG00000010176 | | MAT1A | | methionine adenosyltransferase 1A [Source:HGNC Symbol;Acc:HGNC:6903] | | | | | | | | | | | | | | | |  | |  |
| #INFO: | QUERY: | | ENSOARG00000010181 | | KCTD3 | | potassium channel tetramerization domain containing 3 [Source:HGNC Symbol;Acc:HGNC:21305] | | | | | | | | | | | | | | | |  | |  |
| #INFO: | QUERY: | | ENSOARG00000010198 | | FGF23 | | fibroblast growth factor 23 [Source:HGNC Symbol;Acc:HGNC:3680] | | | | | | | | | | | | | |  | |  | |  |
| #INFO: | QUERY: | | ENSOARG00000010256 | | DYDC1 | | DPY30 domain containing 1 [Source:HGNC Symbol;Acc:HGNC:23460] | | | | | | | | | | | | | |  | |  | |  |
| #INFO: | QUERY: | | ENSOARG00000010271 | | TIGAR | | TP53 induced glycolysis regulatory phosphatase [Source:HGNC Symbol;Acc:HGNC:1185] | | | | | | | | | | | | | | | |  | |  |
| #INFO: | QUERY: | | ENSOARG00000010276 | | CSN1S1 | | alpha-S1-casein precursor [Source:RefSeq peptide;Acc:NP_001009795] | | | | | | | | | | | | | |  | |  | |  |
| #INFO: | QUERY: | | ENSOARG00000010375 | | GUCY1A2 | | guanylate cyclase 1 soluble subunit alpha 2 [Source:HGNC Symbol;Acc:HGNC:4684] | | | | | | | | | | | | | | | |  | |  |
| #INFO: | QUERY: | | ENSOARG00000010477 | | CSN2 | | beta-casein precursor [Source:RefSeq peptide;Acc:NP_001009373] | | | | | | | | | | | | | |  | |  | |  |
| #INFO: | QUERY: | | ENSOARG00000010567 | | N/A | | N/A | |  | |  | |  | |  | |  | |  | |  | |  | |  |
| #INFO: | QUERY: | | ENSOARG00000010714 | | USH2A | | usherin [Source:HGNC Symbol;Acc:HGNC:12601] | | | | | | | | | |  | |  | |  | |  | |  |
| #INFO: | QUERY: | | ENSOARG00000010794 | | N/A | | N/A | |  | |  | |  | |  | |  | |  | |  | |  | |  |
| #INFO: | QUERY: | | ENSOARG00000010804 | | N/A | | N/A | |  | |  | |  | |  | |  | |  | |  | |  | |  |
| #INFO: | QUERY: | | ENSOARG00000010876 | | MRPL46 | | mitochondrial ribosomal protein L46 [Source:HGNC Symbol;Acc:HGNC:1192] | | | | | | | | | | | | | | | |  | |  |
| #INFO: | QUERY: | | ENSOARG00000010887 | | INVS | | inversin [Source:HGNC Symbol;Acc:HGNC:17870] | | | | | | | | | |  | |  | |  | |  | |  |
| #INFO: | QUERY: | | ENSOARG00000010902 | | RCC2 | | regulator of chromosome condensation 2 [Source:HGNC Symbol;Acc:HGNC:30297] | | | | | | | | | | | | | | | |  | |  |
| #INFO: | QUERY: | | ENSOARG00000011161 | | N/A | | N/A | |  | |  | |  | |  | |  | |  | |  | |  | |  |
| #INFO: | QUERY: | | ENSOARG00000011167 | | CMTR2 | | cap methyltransferase 2 [Source:HGNC Symbol;Acc:HGNC:25635] | | | | | | | | | | | | | |  | |  | |  |
| #INFO: | QUERY: | | ENSOARG00000011174 | | C1ORF112 | | chromosome 1 open reading frame 112 [Source:HGNC Symbol;Acc:HGNC:25565] | | | | | | | | | | | | | | | |  | |  |
| #INFO: | QUERY: | | ENSOARG00000011308 | | ZNF345 | | zinc finger protein 345 [Source:HGNC Symbol;Acc:HGNC:16367] | | | | | | | | | | | | | |  | |  | |  |
| #INFO: | QUERY: | | ENSOARG00000011434 | | RRP1 | | ribosomal RNA processing 1 [Source:HGNC Symbol;Acc:HGNC:18785] | | | | | | | | | | | | | |  | |  | |  |
| #INFO: | QUERY: | | ENSOARG00000011451 | | SCYL3 | | SCY1 like pseudokinase 3 [Source:HGNC Symbol;Acc:HGNC:19285] | | | | | | | | | | | | | |  | |  | |  |
| #INFO: | QUERY: | | ENSOARG00000011538 | | N/A | | N/A | |  | |  | |  | |  | |  | |  | |  | |  | |  |
| #INFO: | QUERY: | | ENSOARG00000011596 | | NUDT15 | | nudix hydrolase 15 [Source:HGNC Symbol;Acc:HGNC:23063] | | | | | | | | | | | |  | |  | |  | |  |
| #INFO: | QUERY: | | ENSOARG00000011607 | | EXOC6B | | exocyst complex component 6B [Source:HGNC Symbol;Acc:HGNC:17085] | | | | | | | | | | | | | |  | |  | |  |
| #INFO: | QUERY: | | ENSOARG00000011621 | | N/A | | N/A | |  | |  | |  | |  | |  | |  | |  | |  | |  |
| #INFO: | QUERY: | | ENSOARG00000011624 | | FAM78B | | family with sequence similarity 78 member B [Source:HGNC Symbol;Acc:HGNC:13495] | | | | | | | | | | | | | | | |  | |  |
| #INFO: | QUERY: | | ENSOARG00000011690 | | DIABLO | | diablo IAP-binding mitochondrial protein [Source:HGNC Symbol;Acc:HGNC:21528] | | | | | | | | | | | | | | | |  | |  |
| #INFO: | QUERY: | | ENSOARG00000011742 | | HIPK2 | | homeodomain interacting protein kinase 2 [Source:HGNC Symbol;Acc:HGNC:14402] | | | | | | | | | | | | | | | |  | |  |
| #INFO: | QUERY: | | ENSOARG00000011752 | | LRRC43 | | leucine rich repeat containing 43 [Source:HGNC Symbol;Acc:HGNC:28562] | | | | | | | | | | | | | | | |  | |  |
| #INFO: | QUERY: | | ENSOARG00000011860 | | POMK | | protein-O-mannose kinase [Source:HGNC Symbol;Acc:HGNC:26267] | | | | | | | | | | | | | |  | |  | |  |
| #INFO: | QUERY: | | ENSOARG00000011873 | | N/A | | N/A | |  | |  | |  | |  | |  | |  | |  | |  | |  |
| #INFO: | QUERY: | | ENSOARG00000011884 | | PRKDC | | protein kinase, DNA-activated, catalytic polypeptide [Source:HGNC Symbol;Acc:HGNC:9413] | | | | | | | | | | | | | | | |  | |  |
| #INFO: | QUERY: | | ENSOARG00000011885 | | DPP10 | | dipeptidyl peptidase like 10 [Source:HGNC Symbol;Acc:HGNC:20823] | | | | | | | | | | | | | |  | |  | |  |
| #INFO: | QUERY: | | ENSOARG00000011886 | | ZNF804B | | zinc finger protein 804B [Source:HGNC Symbol;Acc:HGNC:21958] | | | | | | | | | | | | | |  | |  | |  |
| #INFO: | QUERY: | | ENSOARG00000012328 | | MARCO | | macrophage receptor with collagenous structure [Source:HGNC Symbol;Acc:HGNC:6895] | | | | | | | | | | | | | | | |  | |  |
| #INFO: | QUERY: | | ENSOARG00000012354 | | PSAT1 | | phosphoserine aminotransferase 1 [Source:HGNC Symbol;Acc:HGNC:19129] | | | | | | | | | | | | | | | |  | |  |
| #INFO: | QUERY: | | ENSOARG00000012449 | | CDK17 | | cyclin dependent kinase 17 [Source:HGNC Symbol;Acc:HGNC:8750] | | | | | | | | | | | | | |  | |  | |  |
| #INFO: | QUERY: | | ENSOARG00000012452 | | ADARB1 | | adenosine deaminase, RNA specific B1 [Source:HGNC Symbol;Acc:HGNC:226] | | | | | | | | | | | | | | | |  | |  |
| #INFO: | QUERY: | | ENSOARG00000012484 | | PRUNE2 | | prune homolog 2 [Source:HGNC Symbol;Acc:HGNC:25209] | | | | | | | | | | | |  | |  | |  | |  |
| #INFO: | QUERY: | | ENSOARG00000012546 | | CRISP1 | | cysteine rich secretory protein 1 [Source:HGNC Symbol;Acc:HGNC:304] | | | | | | | | | | | | | |  | |  | |  |
| #INFO: | QUERY: | | ENSOARG00000012626 | | TRIP11 | | thyroid hormone receptor interactor 11 [Source:HGNC Symbol;Acc:HGNC:12305] | | | | | | | | | | | | | | | |  | |  |
| #INFO: | QUERY: | | ENSOARG00000012754 | | ATXN3 | | ataxin 3 [Source:HGNC Symbol;Acc:HGNC:7106] | | | | | | | | | |  | |  | |  | |  | |  |
| #INFO: | QUERY: | | ENSOARG00000012797 | | MCM4 | | minichromosome maintenance complex component 4 [Source:HGNC Symbol;Acc:HGNC:6947] | | | | | | | | | | | | | | | |  | |  |
| #INFO: | QUERY: | | ENSOARG00000012915 | | CDH18 | | cadherin 18 [Source:HGNC Symbol;Acc:HGNC:1757] | | | | | | | | | |  | |  | |  | |  | |  |
| #INFO: | QUERY: | | ENSOARG00000013009 | | ATP8A2 | | ATPase phospholipid transporting 8A2 [Source:HGNC Symbol;Acc:HGNC:13533] | | | | | | | | | | | | | | | |  | |  |
| #INFO: | QUERY: | | ENSOARG00000013110 | | PKHD1L1 | | PKHD1 like 1 [Source:HGNC Symbol;Acc:HGNC:20313] | | | | | | | | | | | |  | |  | |  | |  |
| #INFO: | QUERY: | | ENSOARG00000013117 | | N/A | | N/A | |  | |  | |  | |  | |  | |  | |  | |  | |  |
| #INFO: | QUERY: | | ENSOARG00000013129 | | N/A | | N/A | |  | |  | |  | |  | |  | |  | |  | |  | |  |
| #INFO: | QUERY: | | ENSOARG00000013248 | | TMEM189-UBE2V1 | | TMEM189-UBE2V1 readthrough [Source:HGNC Symbol;Acc:HGNC:33521] | | | | | | | | | | | | | |  | |  | |  |
| #INFO: | QUERY: | | ENSOARG00000013337 | | PUM3 | | pumilio RNA binding family member 3 [Source:HGNC Symbol;Acc:HGNC:29676] | | | | | | | | | | | | | | | |  | |  |
| #INFO: | QUERY: | | ENSOARG00000013395 | | N/A | | N/A | |  | |  | |  | |  | |  | |  | |  | |  | |  |
| #INFO: | QUERY: | | ENSOARG00000013447 | | SYCE1 | | synaptonemal complex central element protein 1 [Source:HGNC Symbol;Acc:HGNC:28852] | | | | | | | | | | | | | | | |  | |  |
| #INFO: | QUERY: | | ENSOARG00000013498 | | DNAH5 | | dynein axonemal heavy chain 5 [Source:HGNC Symbol;Acc:HGNC:2950] | | | | | | | | | | | | | |  | |  | |  |
| #INFO: | QUERY: | | ENSOARG00000013508 | | N/A | | N/A | |  | |  | |  | |  | |  | |  | |  | |  | |  |
| #INFO: | QUERY: | | ENSOARG00000013529 | | ADGRL4 | | adhesion G protein-coupled receptor L4 [Source:HGNC Symbol;Acc:HGNC:20822] | | | | | | | | | | | | | | | |  | |  |
| #INFO: | QUERY: | | ENSOARG00000013577 | | CA13 | | carbonic anhydrase 13 [Source:HGNC Symbol;Acc:HGNC:14914] | | | | | | | | | | | | | |  | |  | |  |
| #INFO: | QUERY: | | ENSOARG00000013596 | | COBL | | cordon-bleu WH2 repeat protein [Source:HGNC Symbol;Acc:HGNC:22199] | | | | | | | | | | | | | | | |  | |  |
| #INFO: | QUERY: | | ENSOARG00000013620 | | ADGRL2 | | adhesion G protein-coupled receptor L2 [Source:HGNC Symbol;Acc:HGNC:18582] | | | | | | | | | | | | | | | |  | |  |
| #INFO: | QUERY: | | ENSOARG00000013675 | | N/A | | N/A | |  | |  | |  | |  | |  | |  | |  | |  | |  |
| #INFO: | QUERY: | | ENSOARG00000013756 | | ARHGAP12 | | Rho GTPase activating protein 12 [Source:HGNC Symbol;Acc:HGNC:16348] | | | | | | | | | | | | | | | |  | |  |
| #INFO: | QUERY: | | ENSOARG00000013806 | | N/A | | N/A | |  | |  | |  | |  | |  | |  | |  | |  | |  |
| #INFO: | QUERY: | | ENSOARG00000013841 | | N/A | | N/A | |  | |  | |  | |  | |  | |  | |  | |  | |  |
| #INFO: | QUERY: | | ENSOARG00000013848 | | C14ORF132 | | chromosome 14 open reading frame 132 [Source:HGNC Symbol;Acc:HGNC:20346] | | | | | | | | | | | | | | | |  | |  |
| #INFO: | QUERY: | | ENSOARG00000013854 | | DNASE2B | | deoxyribonuclease 2 beta [Source:HGNC Symbol;Acc:HGNC:28875] | | | | | | | | | | | | | |  | |  | |  |
| #INFO: | QUERY: | | ENSOARG00000013867 | | RPF1 | | ribosome production factor 1 homolog [Source:HGNC Symbol;Acc:HGNC:30350] | | | | | | | | | | | | | | | |  | |  |
| #INFO: | QUERY: | | ENSOARG00000013925 | | GNG5 | | G protein subunit gamma 5 [Source:HGNC Symbol;Acc:HGNC:4408] | | | | | | | | | | | | | |  | |  | |  |
| #INFO: | QUERY: | | ENSOARG00000013942 | | SPATA1 | | spermatogenesis associated 1 [Source:HGNC Symbol;Acc:HGNC:14682] | | | | | | | | | | | | | |  | |  | |  |
| #INFO: | QUERY: | | ENSOARG00000013959 | | CTBS | | chitobiase [Source:HGNC Symbol;Acc:HGNC:2496] | | | | | | | | | |  | |  | |  | |  | |  |
| #INFO: | QUERY: | | ENSOARG00000013977 | | SSX2IP | | SSX family member 2 interacting protein [Source:HGNC Symbol;Acc:HGNC:16509] | | | | | | | | | | | | | | | |  | |  |
| #INFO: | QUERY: | | ENSOARG00000013984 | | SNRPD3 | | small nuclear ribonucleoprotein D3 polypeptide [Source:HGNC Symbol;Acc:HGNC:11160] | | | | | | | | | | | | | | | |  | |  |
| #INFO: | QUERY: | | ENSOARG00000013992 | | CHAF1B | | chromatin assembly factor 1 subunit B [Source:HGNC Symbol;Acc:HGNC:1911] | | | | | | | | | | | | | | | |  | |  |
| #INFO: | QUERY: | | ENSOARG00000013995 | | EPM2AIP1 | | EPM2A interacting protein 1 [Source:HGNC Symbol;Acc:HGNC:19735] | | | | | | | | | | | | | |  | |  | |  |
| #INFO: | QUERY: | | ENSOARG00000014001 | | GABRG2 | | gamma-aminobutyric acid type A receptor gamma2 subunit [Source:HGNC Symbol;Acc:HGNC:4087] | | | | | | | | | | | | | | | |  | |  |
| #INFO: | QUERY: | | ENSOARG00000014032 | | CCDC171 | | coiled-coil domain containing 171 [Source:HGNC Symbol;Acc:HGNC:29828] | | | | | | | | | | | | | | | |  | |  |
| #INFO: | QUERY: | | ENSOARG00000014036 | | GUCD1 | | guanylyl cyclase domain containing 1 [Source:HGNC Symbol;Acc:HGNC:14237] | | | | | | | | | | | | | | | |  | |  |
| #INFO: | QUERY: | | ENSOARG00000014074 | | UPB1 | | beta-ureidopropionase 1 [Source:HGNC Symbol;Acc:HGNC:16297] | | | | | | | | | | | | | |  | |  | |  |
| #INFO: | QUERY: | | ENSOARG00000014084 | | CNTLN | | centlein [Source:HGNC Symbol;Acc:HGNC:23432] | | | | | | | | | |  | |  | |  | |  | |  |
| #INFO: | QUERY: | | ENSOARG00000014188 | | ENPP1 | | ectonucleotide pyrophosphatase/phosphodiesterase 1 [Source:HGNC Symbol;Acc:HGNC:3356] | | | | | | | | | | | | | | | |  | |  |
| #INFO: | QUERY: | | ENSOARG00000014224 | | SLC4A7 | | solute carrier family 4 member 7 [Source:HGNC Symbol;Acc:HGNC:11033] | | | | | | | | | | | | | | | |  | |  |
| #INFO: | QUERY: | | ENSOARG00000014240 | | CTGF | | connective tissue growth factor [Source:HGNC Symbol;Acc:HGNC:2500] | | | | | | | | | | | | | |  | |  | |  |
| #INFO: | QUERY: | | ENSOARG00000014283 | | N/A | | N/A | |  | |  | |  | |  | |  | |  | |  | |  | |  |
| #INFO: | QUERY: | | ENSOARG00000014303 | | STX7 | | syntaxin 7 [Source:HGNC Symbol;Acc:HGNC:11442] | | | | | | | | | |  | |  | |  | |  | |  |
| #INFO: | QUERY: | | ENSOARG00000014305 | | BUB1 | | BUB1 mitotic checkpoint serine/threonine kinase [Source:HGNC Symbol;Acc:HGNC:1148] | | | | | | | | | | | | | | | |  | |  |
| #INFO: | QUERY: | | ENSOARG00000014323 | | ECHDC3 | | enoyl-CoA hydratase domain containing 3 [Source:HGNC Symbol;Acc:HGNC:23489] | | | | | | | | | | | | | | | |  | |  |
| #INFO: | QUERY: | | ENSOARG00000014361 | | SEMA5A | | semaphorin 5A [Source:HGNC Symbol;Acc:HGNC:10736] | | | | | | | | | | | |  | |  | |  | |  |
| #INFO: | QUERY: | | ENSOARG00000014403 | | N/A | | N/A | |  | |  | |  | |  | |  | |  | |  | |  | |  |
| #INFO: | QUERY: | | ENSOARG00000014502 | | N/A | | N/A | |  | |  | |  | |  | |  | |  | |  | |  | |  |
| #INFO: | QUERY: | | ENSOARG00000014527 | | N/A | | N/A | |  | |  | |  | |  | |  | |  | |  | |  | |  |
| #INFO: | QUERY: | | ENSOARG00000014596 | | TMEM74 | | transmembrane protein 74 [Source:HGNC Symbol;Acc:HGNC:26409] | | | | | | | | | | | | | |  | |  | |  |
| #INFO: | QUERY: | | ENSOARG00000014628 | | N/A | | N/A | |  | |  | |  | |  | |  | |  | |  | |  | |  |
| #INFO: | QUERY: | | ENSOARG00000014642 | | N/A | | N/A | |  | |  | |  | |  | |  | |  | |  | |  | |  |
| #INFO: | QUERY: | | ENSOARG00000014661 | | RNF144A | | ring finger protein 144A [Source:HGNC Symbol;Acc:HGNC:20457] | | | | | | | | | | | | | |  | |  | |  |
| #INFO: | QUERY: | | ENSOARG00000014684 | | RBMS3 | | RNA binding motif single stranded interacting protein 3 [Source:HGNC Symbol;Acc:HGNC:13427] | | | | | | | | | | | | | | | |  | |  |
| #INFO: | QUERY: | | ENSOARG00000014703 | | MYL10 | | myosin light chain 10 [Source:HGNC Symbol;Acc:HGNC:29825] | | | | | | | | | | | |  | |  | |  | |  |
| #INFO: | QUERY: | | ENSOARG00000014711 | | COL26A1 | | collagen type XXVI alpha 1 chain [Source:HGNC Symbol;Acc:HGNC:18038] | | | | | | | | | | | | | | | |  | |  |
| #INFO: | QUERY: | | ENSOARG00000014718 | | COL6A6 | | collagen type VI alpha 6 chain [Source:HGNC Symbol;Acc:HGNC:27023] | | | | | | | | | | | | | |  | |  | |  |
| #INFO: | QUERY: | | ENSOARG00000014720 | | N/A | | N/A | |  | |  | |  | |  | |  | |  | |  | |  | |  |
| #INFO: | QUERY: | | ENSOARG00000014737 | | N/A | | N/A | |  | |  | |  | |  | |  | |  | |  | |  | |  |
| #INFO: | QUERY: | | ENSOARG00000014852 | | BBS10 | | Bardet-Biedl syndrome 10 [Source:HGNC Symbol;Acc:HGNC:26291] | | | | | | | | | | | | | |  | |  | |  |
| #INFO: | QUERY: | | ENSOARG00000014876 | | OSBPL8 | | oxysterol binding protein like 8 [Source:HGNC Symbol;Acc:HGNC:16396] | | | | | | | | | | | | | |  | |  | |  |
| #INFO: | QUERY: | | ENSOARG00000014972 | | MED13 | | mediator complex subunit 13 [Source:HGNC Symbol;Acc:HGNC:22474] | | | | | | | | | | | | | |  | |  | |  |
| #INFO: | QUERY: | | ENSOARG00000014987 | | N/A | | N/A | |  | |  | |  | |  | |  | |  | |  | |  | |  |
| #INFO: | QUERY: | | ENSOARG00000015055 | | KIF13B | | kinesin family member 13B [Source:HGNC Symbol;Acc:HGNC:14405] | | | | | | | | | | | | | |  | |  | |  |
| #INFO: | QUERY: | | ENSOARG00000015068 | | SLC10A5 | | solute carrier family 10 member 5 [Source:HGNC Symbol;Acc:HGNC:22981] | | | | | | | | | | | | | | | |  | |  |
| #INFO: | QUERY: | | ENSOARG00000015098 | | N/A | | N/A | |  | |  | |  | |  | |  | |  | |  | |  | |  |
| #INFO: | QUERY: | | ENSOARG00000015124 | | PTPRQ | | protein tyrosine phosphatase, receptor type Q [Source:HGNC Symbol;Acc:HGNC:9679] | | | | | | | | | | | | | | | |  | |  |
| #INFO: | QUERY: | | ENSOARG00000015154 | | ZPBP | | zona pellucida binding protein [Source:HGNC Symbol;Acc:HGNC:15662] | | | | | | | | | | | | | |  | |  | |  |
| #INFO: | QUERY: | | ENSOARG00000015220 | | PPFIA2 | | PTPRF interacting protein alpha 2 [Source:HGNC Symbol;Acc:HGNC:9246] | | | | | | | | | | | | | | | |  | |  |
| #INFO: | QUERY: | | ENSOARG00000015226 | | N/A | | N/A | |  | |  | |  | |  | |  | |  | |  | |  | |  |
| #INFO: | QUERY: | | ENSOARG00000015257 | | CRB1 | | crumbs 1, cell polarity complex component [Source:HGNC Symbol;Acc:HGNC:2343] | | | | | | | | | | | | | | | |  | |  |
| #INFO: | QUERY: | | ENSOARG00000015297 | | GBP5 | | guanylate binding protein 5 [Source:HGNC Symbol;Acc:HGNC:19895] | | | | | | | | | | | | | |  | |  | |  |
| #INFO: | QUERY: | | ENSOARG00000015367 | | BNIP3 | | BCL2 interacting protein 3 [Source:HGNC Symbol;Acc:HGNC:1084] | | | | | | | | | | | | | |  | |  | |  |
| #INFO: | QUERY: | | ENSOARG00000015414 | | N/A | | N/A | |  | |  | |  | |  | |  | |  | |  | |  | |  |
| #INFO: | QUERY: | | ENSOARG00000015421 | | MGAT4C | | MGAT4 family member C [Source:HGNC Symbol;Acc:HGNC:30871] | | | | | | | | | | | | | |  | |  | |  |
| #INFO: | QUERY: | | ENSOARG00000015432 | | N/A | | N/A | |  | |  | |  | |  | |  | |  | |  | |  | |  |
| #INFO: | QUERY: | | ENSOARG00000015436 | | N/A | | N/A | |  | |  | |  | |  | |  | |  | |  | |  | |  |
| #INFO: | QUERY: | | ENSOARG00000015449 | | N/A | | N/A | |  | |  | |  | |  | |  | |  | |  | |  | |  |
| #INFO: | QUERY: | | ENSOARG00000015457 | | ENPP2 | | ectonucleotide pyrophosphatase/phosphodiesterase 2 [Source:HGNC Symbol;Acc:HGNC:3357] | | | | | | | | | | | | | | | |  | |  |
| #INFO: | QUERY: | | ENSOARG00000015488 | | N/A | | N/A | |  | |  | |  | |  | |  | |  | |  | |  | |  |
| #INFO: | QUERY: | | ENSOARG00000015495 | | ZFPM2 | | zinc finger protein, FOG family member 2 [Source:HGNC Symbol;Acc:HGNC:16700] | | | | | | | | | | | | | | | |  | |  |
| #INFO: | QUERY: | | ENSOARG00000015593 | | CEP112 | | centrosomal protein 112 [Source:HGNC Symbol;Acc:HGNC:28514] | | | | | | | | | | | | | |  | |  | |  |
| #INFO: | QUERY: | | ENSOARG00000015661 | | APOH | | apolipoprotein H [Source:HGNC Symbol;Acc:HGNC:616] | | | | | | | | | | | |  | |  | |  | |  |
| #INFO: | QUERY: | | ENSOARG00000015751 | | C2ORF50 | | chromosome 2 open reading frame 50 [Source:HGNC Symbol;Acc:HGNC:26324] | | | | | | | | | | | | | | | |  | |  |
| #INFO: | QUERY: | | ENSOARG00000015761 | | HTR7 | | 5-hydroxytryptamine receptor 7 [Source:HGNC Symbol;Acc:HGNC:5302] | | | | | | | | | | | | | |  | |  | |  |
| #INFO: | QUERY: | | ENSOARG00000015789 | | RPP30 | | ribonuclease P/MRP subunit p30 [Source:HGNC Symbol;Acc:HGNC:17688] | | | | | | | | | | | | | | | |  | |  |
| #INFO: | QUERY: | | ENSOARG00000015808 | | PQLC3 | | PQ loop repeat containing 3 [Source:HGNC Symbol;Acc:HGNC:28503] | | | | | | | | | | | | | |  | |  | |  |
| #INFO: | QUERY: | | ENSOARG00000015894 | | N/A | | N/A | |  | |  | |  | |  | |  | |  | |  | |  | |  |
| #INFO: | QUERY: | | ENSOARG00000015908 | | ANKRD1 | | ankyrin repeat domain 1 [Source:HGNC Symbol;Acc:HGNC:15819] | | | | | | | | | | | | | |  | |  | |  |
| #INFO: | QUERY: | | ENSOARG00000015947 | | GLRX5 | | glutaredoxin 5 [Source:HGNC Symbol;Acc:HGNC:20134] | | | | | | | | | | | |  | |  | |  | |  |
| #INFO: | QUERY: | | ENSOARG00000015953 | | TCL1B | | T-cell leukemia/lymphoma 1B [Source:HGNC Symbol;Acc:HGNC:11649] | | | | | | | | | | | | | |  | |  | |  |
| #INFO: | QUERY: | | ENSOARG00000015981 | | TCL1A | | T-cell leukemia/lymphoma 1A [Source:HGNC Symbol;Acc:HGNC:11648] | | | | | | | | | | | | | |  | |  | |  |
| #INFO: | QUERY: | | ENSOARG00000016010 | | N/A | | N/A | |  | |  | |  | |  | |  | |  | |  | |  | |  |
| #INFO: | QUERY: | | ENSOARG00000016040 | | HECTD2 | | HECT domain E3 ubiquitin protein ligase 2 [Source:HGNC Symbol;Acc:HGNC:26736] | | | | | | | | | | | | | | | |  | |  |
| #INFO: | QUERY: | | ENSOARG00000016056 | | FBXL3 | | F-box/LRR-repeat protein 3 [Source:RefSeq peptide;Acc:NP_001123211] | | | | | | | | | | | | | |  | |  | |  |
| #INFO: | QUERY: | | ENSOARG00000016172 | | N/A | | N/A | |  | |  | |  | |  | |  | |  | |  | |  | |  |
| #INFO: | QUERY: | | ENSOARG00000016318 | | N/A | | N/A | |  | |  | |  | |  | |  | |  | |  | |  | |  |
| #INFO: | QUERY: | | ENSOARG00000016340 | | NBAS | | neuroblastoma amplified sequence [Source:HGNC Symbol;Acc:HGNC:15625] | | | | | | | | | | | | | | | |  | |  |
| #INFO: | QUERY: | | ENSOARG00000016384 | | CDK14 | | cyclin dependent kinase 14 [Source:HGNC Symbol;Acc:HGNC:8883] | | | | | | | | | | | | | |  | |  | |  |
| #INFO: | QUERY: | | ENSOARG00000016410 | | WDR75 | | WD repeat domain 75 [Source:HGNC Symbol;Acc:HGNC:25725] | | | | | | | | | | | | | |  | |  | |  |
| #INFO: | QUERY: | | ENSOARG00000016502 | | ADGRV1 | | adhesion G protein-coupled receptor V1 [Source:HGNC Symbol;Acc:HGNC:17416] | | | | | | | | | | | | | | | |  | |  |
| #INFO: | QUERY: | | ENSOARG00000016515 | | CNIH3 | | cornichon family AMPA receptor auxiliary protein 3 [Source:HGNC Symbol;Acc:HGNC:26802] | | | | | | | | | | | | | | | |  | |  |
| #INFO: | QUERY: | | ENSOARG00000016532 | | N/A | | N/A | |  | |  | |  | |  | |  | |  | |  | |  | |  |
| #INFO: | QUERY: | | ENSOARG00000016550 | | MLH1 | | mutL homolog 1 [Source:HGNC Symbol;Acc:HGNC:7127] | | | | | | | | | | | |  | |  | |  | |  |
| #INFO: | QUERY: | | ENSOARG00000016604 | | GLT1D1 | | glycosyltransferase 1 domain containing 1 [Source:HGNC Symbol;Acc:HGNC:26483] | | | | | | | | | | | | | | | |  | |  |
| #INFO: | QUERY: | | ENSOARG00000016758 | | N/A | | N/A | |  | |  | |  | |  | |  | |  | |  | |  | |  |
| #INFO: | QUERY: | | ENSOARG00000016960 | | CRHBP | | corticotropin releasing hormone binding protein [Source:HGNC Symbol;Acc:HGNC:2356] | | | | | | | | | | | | | | | |  | |  |
| #INFO: | QUERY: | | ENSOARG00000016999 | | DNTTIP2 | | deoxynucleotidyltransferase terminal interacting protein 2 [Source:HGNC Symbol;Acc:HGNC:24013] | | | | | | | | | | | | | | | |  | |  |
| #INFO: | QUERY: | | ENSOARG00000017012 | | GCLM | | glutamate-cysteine ligase modifier subunit [Source:HGNC Symbol;Acc:HGNC:4312] | | | | | | | | | | | | | | | |  | |  |
| #INFO: | QUERY: | | ENSOARG00000017066 | | N/A | | N/A | |  | |  | |  | |  | |  | |  | |  | |  | |  |
| #INFO: | QUERY: | | ENSOARG00000017145 | | N/A | | N/A | |  | |  | |  | |  | |  | |  | |  | |  | |  |
| #INFO: | QUERY: | | ENSOARG00000017215 | | N/A | | N/A | |  | |  | |  | |  | |  | |  | |  | |  | |  |
| #INFO: | QUERY: | | ENSOARG00000017222 | | SPATA5 | | spermatogenesis associated 5 [Source:HGNC Symbol;Acc:HGNC:18119] | | | | | | | | | | | | | |  | |  | |  |
| #INFO: | QUERY: | | ENSOARG00000017228 | | N/A | | N/A | |  | |  | |  | |  | |  | |  | |  | |  | |  |
| #INFO: | QUERY: | | ENSOARG00000017361 | | ARHGEF12 | | Rho guanine nucleotide exchange factor 12 [Source:HGNC Symbol;Acc:HGNC:14193] | | | | | | | | | | | | | | | |  | |  |
| #INFO: | QUERY: | | ENSOARG00000017524 | | N/A | | N/A | |  | |  | |  | |  | |  | |  | |  | |  | |  |
| #INFO: | QUERY: | | ENSOARG00000017578 | | N/A | | N/A | |  | |  | |  | |  | |  | |  | |  | |  | |  |
| #INFO: | QUERY: | | ENSOARG00000017683 | | PALMD | | palmdelphin [Source:HGNC Symbol;Acc:HGNC:15846] | | | | | | | | | | | |  | |  | |  | |  |
| #INFO: | QUERY: | | ENSOARG00000017700 | | FRRS1 | | ferric chelate reductase 1 [Source:HGNC Symbol;Acc:HGNC:27622] | | | | | | | | | | | | | |  | |  | |  |
| #INFO: | QUERY: | | ENSOARG00000017760 | | CCDC192 | | coiled-coil domain containing 192 [Source:HGNC Symbol;Acc:HGNC:49566] | | | | | | | | | | | | | | | |  | |  |
| #INFO: | QUERY: | | ENSOARG00000017762 | | AGL | | amylo-alpha-1, 6-glucosidase, 4-alpha-glucanotransferase [Source:HGNC Symbol;Acc:HGNC:321] | | | | | | | | | | | | | | | |  | |  |
| #INFO: | QUERY: | | ENSOARG00000017783 | | UBAP1L | | ubiquitin associated protein 1 like [Source:HGNC Symbol;Acc:HGNC:40028] | | | | | | | | | | | | | | | |  | |  |
| #INFO: | QUERY: | | ENSOARG00000017838 | | AMPH | | amphiphysin [Source:HGNC Symbol;Acc:HGNC:471] | | | | | | | | | |  | |  | |  | |  | |  |
| #INFO: | QUERY: | | ENSOARG00000017849 | | SLC35A3 | | solute carrier family 35 member A3 [Source:HGNC Symbol;Acc:HGNC:11023] | |  | |  | |  | |  | |  | |  | |  | |  | |  |
| #INFO: | QUERY: | | ENSOARG00000017887 | | RPGRIP1L | | RPGRIP1 like [Source:HGNC Symbol;Acc:HGNC:29168] | | | | | | | | | | | |  | |  | |  | |  |
| #INFO: | QUERY: | | ENSOARG00000017944 | | N/A | | N/A | |  | |  | |  | |  | |  | |  | |  | |  | |  |
| #INFO: | QUERY: | | ENSOARG00000018069 | | NDST4 | | N-deacetylase and N-sulfotransferase 4 [Source:HGNC Symbol;Acc:HGNC:20779] | | | | | | | | | | | | | | | |  | |  |
| #INFO: | QUERY: | | ENSOARG00000018151 | | DIS3L | | DIS3 like exosome 3'-5' exoribonuclease [Source:HGNC Symbol;Acc:HGNC:28698] | | | | | | | | | | | | | | | |  | |  |
| #INFO: | QUERY: | | ENSOARG00000018241 | | GRID2 | | glutamate ionotropic receptor delta type subunit 2 [Source:HGNC Symbol;Acc:HGNC:4576] | | | | | | | | | | | | | | | |  | |  |
| #INFO: | QUERY: | | ENSOARG00000018287 | | N/A | | N/A | |  | |  | |  | |  | |  | |  | |  | |  | |  |
| #INFO: | QUERY: | | ENSOARG00000018342 | | CCSER1 | | coiled-coil serine rich protein 1 [Source:HGNC Symbol;Acc:HGNC:29349] | | | | | | | | | | | | | |  | |  | |  |
| #INFO: | QUERY: | | ENSOARG00000018363 | | N/A | | N/A | |  | |  | |  | |  | |  | |  | |  | |  | |  |
| #INFO: | QUERY: | | ENSOARG00000018380 | | N/A | | N/A | |  | |  | |  | |  | |  | |  | |  | |  | |  |
| #INFO: | QUERY: | | ENSOARG00000018455 | | OLFM3 | | olfactomedin 3 [Source:HGNC Symbol;Acc:HGNC:17990] | | | | | | | | | | | |  | |  | |  | |  |
| #INFO: | QUERY: | | ENSOARG00000018504 | | TPK1 | | thiamin pyrophosphokinase 1 [Source:HGNC Symbol;Acc:HGNC:17358] | | | | | | | | | | | | | |  | |  | |  |
| #INFO: | QUERY: | | ENSOARG00000018561 | | N/A | | N/A | |  | |  | |  | |  | |  | |  | |  | |  | |  |
| #INFO: | QUERY: | | ENSOARG00000018604 | | GLCE | | glucuronic acid epimerase [Source:HGNC Symbol;Acc:HGNC:17855] | | | | | | | | | | | | | |  | |  | |  |
| #INFO: | QUERY: | | ENSOARG00000018675 | | NDUFA10 | | NADH:ubiquinone oxidoreductase subunit A10 [Source:HGNC Symbol;Acc:HGNC:7684] | | | | | | | | | | | | | | | |  | |  |
| #INFO: | QUERY: | | ENSOARG00000018779 | | TRAF3IP1 | | TRAF3 interacting protein 1 [Source:HGNC Symbol;Acc:HGNC:17861] | | | | | | | | | | | | | |  | |  | |  |
| #INFO: | QUERY: | | ENSOARG00000018809 | | CD44 | | CD44 molecule (Indian blood group) [Source:HGNC Symbol;Acc:HGNC:1681] | | | | | | | | | | | | | | | |  | |  |
| #INFO: | QUERY: | | ENSOARG00000018985 | | CRYBB3 | | crystallin beta B3 [Source:HGNC Symbol;Acc:HGNC:2400] | | | | | | | | | | | |  | |  | |  | |  |
| #INFO: | QUERY: | | ENSOARG00000019007 | | CRYBB2 | | crystallin beta B2 [Source:HGNC Symbol;Acc:HGNC:2398] | | | | | | | | | | | |  | |  | |  | |  |
| #INFO: | QUERY: | | ENSOARG00000019033 | | N/A | | N/A | |  | |  | |  | |  | |  | |  | |  | |  | |  |
| #INFO: | QUERY: | | ENSOARG00000019099 | | N/A | | N/A | |  | |  | |  | |  | |  | |  | |  | |  | |  |
| #INFO: | QUERY: | | ENSOARG00000019119 | | IQCA1 | | IQ motif containing with AAA domain 1 [Source:HGNC Symbol;Acc:HGNC:26195] | | | | | | | | | | | | | | | |  | |  |
| #INFO: | QUERY: | | ENSOARG00000019130 | | N/A | | N/A | |  | |  | |  | |  | |  | |  | |  | |  | |  |
| #INFO: | QUERY: | | ENSOARG00000019144 | | N/A | | N/A | |  | |  | |  | |  | |  | |  | |  | |  | |  |
| #INFO: | QUERY: | | ENSOARG00000019163 | | N/A | | N/A | |  | |  | |  | |  | |  | |  | |  | |  | |  |
| #INFO: | QUERY: | | ENSOARG00000019174 | | N/A | | N/A | |  | |  | |  | |  | |  | |  | |  | |  | |  |
| #INFO: | QUERY: | | ENSOARG00000019176 | | N/A | | N/A | |  | |  | |  | |  | |  | |  | |  | |  | |  |
| #INFO: | QUERY: | | ENSOARG00000019388 | | CSMD2 | | CUB and Sushi multiple domains 2 [Source:HGNC Symbol;Acc:HGNC:19290] | | | | | | | | | | | | | | | |  | |  |
| #INFO: | QUERY: | | ENSOARG00000019554 | | ARID2 | | AT-rich interaction domain 2 [Source:HGNC Symbol;Acc:HGNC:18037] | | | | | | | | | | | | | |  | |  | |  |
| #INFO: | QUERY: | | ENSOARG00000019588 | | OVCH1 | | ovochymase 1 [Source:HGNC Symbol;Acc:HGNC:23080] | | | | | | | | | | | |  | |  | |  | |  |
| #INFO: | QUERY: | | ENSOARG00000019623 | | ERGIC2 | | ERGIC and golgi 2 [Source:HGNC Symbol;Acc:HGNC:30208] | | | | | | | | | | | |  | |  | |  | |  |
| #INFO: | QUERY: | | ENSOARG00000019747 | | GRIK3 | | glutamate ionotropic receptor kainate type subunit 3 [Source:HGNC Symbol;Acc:HGNC:4581] | | | | | | | | | | | | | | | |  | |  |
| #INFO: | QUERY: | | ENSOARG00000020089 | | N/A | | N/A | |  | |  | |  | |  | |  | |  | |  | |  | |  |
| #INFO: | QUERY: | | ENSOARG00000020091 | | HIST1H2BA | | histone cluster 1 H2B family member a [Source:HGNC Symbol;Acc:HGNC:18730] | | | | | | | | | | | | | | | |  | |  |
| #INFO: | QUERY: | | ENSOARG00000020138 | | N/A | | N/A | |  | |  | |  | |  | |  | |  | |  | |  | |  |
| #INFO: | QUERY: | | ENSOARG00000020167 | | DIRC2 | | disrupted in renal carcinoma 2 [Source:HGNC Symbol;Acc:HGNC:16628] | | | | | | | | | | | | | |  | |  | |  |
| #INFO: | QUERY: | | ENSOARG00000020441 | | MB21D2 | | Mab-21 domain containing 2 [Source:HGNC Symbol;Acc:HGNC:30438] | | | | | | | | | | | | | |  | |  | |  |
| #INFO: | QUERY: | | ENSOARG00000020442 | | NOTCH2 | | notch 2 [Source:HGNC Symbol;Acc:HGNC:7882] | | | | | | | | | |  | |  | |  | |  | |  |
| #INFO: | QUERY: | | ENSOARG00000020469 | | CLDN16 | | claudin 16 [Source:HGNC Symbol;Acc:HGNC:2037] | | | | | | | | | |  | |  | |  | |  | |  |
| #INFO: | QUERY: | | ENSOARG00000020593 | | PID1 | | phosphotyrosine interaction domain containing 1 [Source:HGNC Symbol;Acc:HGNC:26084] | | | | | | | | | | | | | | | |  | |  |
| #INFO: | QUERY: | | ENSOARG00000020740 | | DIS3L2 | | DIS3 like 3'-5' exoribonuclease 2 [Source:HGNC Symbol;Acc:HGNC:28648] | | | | | | | | | | | | | | | |  | |  |
| #INFO: | QUERY: | | ENSOARG00000020974 | | N/A | | N/A | |  | |  | |  | |  | |  | |  | |  | |  | |  |
| #INFO: | QUERY: | | ENSOARG00000020976 | | GLDN | | gliomedin [Source:HGNC Symbol;Acc:HGNC:29514] | | | | | | | | | |  | |  | |  | |  | |  |
| #INFO: | QUERY: | | ENSOARG00000021090 | | N/A | | N/A | |  | |  | |  | |  | |  | |  | |  | |  | |  |
| #INFO: | QUERY: | | ENSOARG00000021199 | | SIPA1L1 | | signal induced proliferation associated 1 like 1 [Source:HGNC Symbol;Acc:HGNC:20284] | | | | | | | | | | | | | | | |  | |  |
| #INFO: | QUERY: | | ENSOARG00000021212 | | SNORD113 | | Small nucleolar RNA SNORD113/SNORD114 family [Source:RFAM;Acc:RF00181] | | | | | | | | | | | | | | | |  | |  |
| #INFO: | QUERY: | | ENSOARG00000021221 | | SNORD113 | | Small nucleolar RNA SNORD113/SNORD114 family [Source:RFAM;Acc:RF00181] | | | | | | | | | | | | | | | |  | |  |
| #INFO: | QUERY: | | ENSOARG00000021264 | | N/A | | N/A | |  | |  | |  | |  | |  | |  | |  | |  | |  |
| #INFO: | QUERY: | | ENSOARG00000021304 | | 5S_RRNA | | 5S ribosomal RNA [Source:RFAM;Acc:RF00001] | | | | | | | | | |  | |  | |  | |  | |  |
| #INFO: | QUERY: | | ENSOARG00000021329 | | U6 | | U6 spliceosomal RNA [Source:RFAM;Acc:RF00026] | |  | |  | |  | |  | |  | |  | |  | |  | |  |
| #INFO: | QUERY: | | ENSOARG00000021350 | | U6 | | U6 spliceosomal RNA [Source:RFAM;Acc:RF00026] | | | | | | | | | |  | |  | |  | |  | |  |
| #INFO: | QUERY: | | ENSOARG00000021369 | | N/A | | N/A | |  | |  | |  | |  | |  | |  | |  | |  | |  |
| #INFO: | QUERY: | | ENSOARG00000021408 | | OAR-MIR-1197 | | oar-mir-1197 [Source:miRBase;Acc:MI0016922] | | | | | | | | | |  | |  | |  | |  | |  |
| #INFO: | QUERY: | | ENSOARG00000021410 | | OAR-MIR-411B | | oar-mir-411b [Source:miRBase;Acc:MI0016921] | | | | | | | | | |  | |  | |  | |  | |  |
| #INFO: | QUERY: | | ENSOARG00000021417 | | OAR-MIR-1193 | | oar-mir-1193 [Source:miRBase;Acc:MI0016928] | | | | | | | | | |  | |  | |  | |  | |  |
| #INFO: | QUERY: | | ENSOARG00000021439 | | U6 | | U6 spliceosomal RNA [Source:RFAM;Acc:RF00026] | | | | | | | | | |  | |  | |  | |  | |  |
| #INFO: | QUERY: | | ENSOARG00000021503 | | U6 | | U6 spliceosomal RNA [Source:RFAM;Acc:RF00026] | | | | | | | | | |  | |  | |  | |  | |  |
| #INFO: | QUERY: | | ENSOARG00000021557 | | SNORD113 | | Small nucleolar RNA SNORD113/SNORD114 family [Source:RFAM;Acc:RF00181] | | | | | | | | | | | | | | | |  | |  |
| #INFO: | QUERY: | | ENSOARG00000021558 | | N/A | | N/A | |  | |  | |  | |  | |  | |  | |  | |  | |  |
| #INFO: | QUERY: | | ENSOARG00000021651 | | N/A | | N/A | |  | |  | |  | |  | |  | |  | |  | |  | |  |
| #INFO: | QUERY: | | ENSOARG00000021660 | | U6 | | U6 spliceosomal RNA [Source:RFAM;Acc:RF00026] | | | | | | | | | |  | |  | |  | |  | |  |
| #INFO: | QUERY: | | ENSOARG00000021819 | | SNORD113 | | Small nucleolar RNA SNORD113/SNORD114 family [Source:RFAM;Acc:RF00181] | | | | | | | | | | | | | | | |  | |  |
| #INFO: | QUERY: | | ENSOARG00000021829 | | N/A | | N/A | |  | |  | |  | |  | |  | |  | |  | |  | |  |
| #INFO: | QUERY: | | ENSOARG00000021941 | | SNORD113 | | Small nucleolar RNA SNORD113/SNORD114 family [Source:RFAM;Acc:RF00181] | | | | | | | | | | | | | | | |  | |  |
| #INFO: | QUERY: | | ENSOARG00000021975 | | N/A | | N/A | |  | |  | |  | |  | |  | |  | |  | |  | |  |
| #INFO: | QUERY: | | ENSOARG00000021980 | | SNORD113 | | Small nucleolar RNA SNORD113/SNORD114 family [Source:RFAM;Acc:RF00181] | | | | | | | | | | | | | | | |  | |  |
| #INFO: | QUERY: | | ENSOARG00000021988 | | SNORD113 | | Small nucleolar RNA SNORD113/SNORD114 family [Source:RFAM;Acc:RF00181] | | | | | | | | | | | | | | | |  | |  |
| #INFO: | QUERY: | | ENSOARG00000022074 | | U6 | | U6 spliceosomal RNA [Source:RFAM;Acc:RF00026] | | | | | | | | | |  | |  | |  | |  | |  |
| #INFO: | QUERY: | | ENSOARG00000022080 | | 5S_RRNA | | 5S ribosomal RNA [Source:RFAM;Acc:RF00001] | | | | | | | | | |  | |  | |  | |  | |  |
| #INFO: | QUERY: | | ENSOARG00000022087 | | SNORD123 | | Small nucleolar RNA SNORD123 [Source:RFAM;Acc:RF01156] | | | | | | | | | | | |  | |  | |  | |  |
| #INFO: | QUERY: | | ENSOARG00000022155 | | OAR-MIR-379 | | oar-mir-379 [Source:miRBase;Acc:MI0016917] | | | | | | | | | |  | |  | |  | |  | |  |
| #INFO: | QUERY: | | ENSOARG00000022247 | | OAR-MIR-329B | | oar-mir-329b [Source:miRBase;Acc:MI0016925] | | | | | | | | | |  | |  | |  | |  | |  |
| #INFO: | QUERY: | | ENSOARG00000022309 | | SNORD113 | | Small nucleolar RNA SNORD113/SNORD114 family [Source:RFAM;Acc:RF00181] | | | | | | | | | | | | | | | |  | |  |
| #INFO: | QUERY: | | ENSOARG00000022316 | | SNORD113 | | Small nucleolar RNA SNORD113/SNORD114 family [Source:RFAM;Acc:RF00181] | | | | | | | | | | | | | | | |  | |  |
| #INFO: | QUERY: | | ENSOARG00000022341 | | SNORD113 | | Small nucleolar RNA SNORD113/SNORD114 family [Source:RFAM;Acc:RF00181] | | | | | | | | | | | | | | | |  | |  |
| #INFO: | QUERY: | | ENSOARG00000022345 | | SNORA62 | | Small nucleolar RNA SNORA62/SNORA6 family [Source:RFAM;Acc:RF00091] | | | | | | | | | | | | | | | |  | |  |
| #INFO: | QUERY: | | ENSOARG00000022355 | | U6 | | U6 spliceosomal RNA [Source:RFAM;Acc:RF00026] | | | | | | | | | |  | |  | |  | |  | |  |
| #INFO: | QUERY: | | ENSOARG00000022429 | | U6 | | U6 spliceosomal RNA [Source:RFAM;Acc:RF00026] | | | | | | | | | |  | |  | |  | |  | |  |
| #INFO: | QUERY: | | ENSOARG00000022460 | | 5S_RRNA | | 5S ribosomal RNA [Source:RFAM;Acc:RF00001] | | | | | | | | | |  | |  | |  | |  | |  |
| #INFO: | QUERY: | | ENSOARG00000022489 | | OAR-MIR-323A | | oar-mir-323a [Source:miRBase;Acc:MI0016923] | | | | | | | | | |  | |  | |  | |  | |  |
| #INFO: | QUERY: | | ENSOARG00000022506 | | OAR-MIR-329A | | oar-mir-329a [Source:miRBase;Acc:MI0016926] | | | | | | | | | |  | |  | |  | |  | |  |
| #INFO: | QUERY: | | ENSOARG00000022534 | | SNORD113 | | Small nucleolar RNA SNORD113/SNORD114 family [Source:RFAM;Acc:RF00181] | | | | | | | | | | | | | | | |  | |  |
| #INFO: | QUERY: | | ENSOARG00000022543 | | U6 | | U6 spliceosomal RNA [Source:RFAM;Acc:RF00026] | | | | | | | | | |  | |  | |  | |  | |  |
| #INFO: | QUERY: | | ENSOARG00000022572 | | SNORD113 | | Small nucleolar RNA SNORD113/SNORD114 family [Source:RFAM;Acc:RF00181] | | | | | | | | | | | | | | | |  | |  |
| #INFO: | QUERY: | | ENSOARG00000022614 | | OAR-MIR-380 | | oar-mir-380 [Source:miRBase;Acc:MI0016920] | | | | | | | | | |  | |  | |  | |  | |  |
| #INFO: | QUERY: | | ENSOARG00000022659 | | SNORD113 | | Small nucleolar RNA SNORD113/SNORD114 family [Source:RFAM;Acc:RF00181] | | | | | | | | | | | | | | | |  | |  |
| #INFO: | QUERY: | | ENSOARG00000022660 | | N/A | | N/A | |  | |  | |  | |  | |  | |  | |  | |  | |  |
| #INFO: | QUERY: | | ENSOARG00000022727 | | N/A | | N/A | |  | |  | |  | |  | |  | |  | |  | |  | |  |
| #INFO: | QUERY: | | ENSOARG00000022802 | | U6 | | U6 spliceosomal RNA [Source:RFAM;Acc:RF00026] | | | | | | | | | |  | |  | |  | |  | |  |
| #INFO: | QUERY: | | ENSOARG00000022955 | | 7SK | | 7SK RNA [Source:RFAM;Acc:RF00100] | | | | | | | |  | |  | |  | |  | |  | |  |
| #INFO: | QUERY: | | ENSOARG00000023114 | | U6 | | U6 spliceosomal RNA [Source:RFAM;Acc:RF00026] | | | | | | | | | |  | |  | |  | |  | |  |
| #INFO: | QUERY: | | ENSOARG00000023129 | | U6 | | U6 spliceosomal RNA [Source:RFAM;Acc:RF00026] | | | | | | | | | |  | |  | |  | |  | |  |
| #INFO: | QUERY: | | ENSOARG00000023140 | | SNORD113 | | Small nucleolar RNA SNORD113/SNORD114 family [Source:RFAM;Acc:RF00181] | | | | | | | | | | | | | | | |  | |  |
| #INFO: | QUERY: | | ENSOARG00000023165 | | U6 | | U6 spliceosomal RNA [Source:RFAM;Acc:RF00026] | | | | | | | | | |  | |  | |  | |  | |  |
| #INFO: | QUERY: | | ENSOARG00000023438 | | TCL6_3 | | T-cell leukemia/lymphoma 6 conserved region 3 [Source:RFAM;Acc:RF02193] | | | | | | | | | | | | | | | |  | |  |
| #INFO: | QUERY: | | ENSOARG00000023442 | | U6 | | U6 spliceosomal RNA [Source:RFAM;Acc:RF00026] | | | | | | | | | |  | |  | |  | |  | |  |
| #INFO: | QUERY: | | ENSOARG00000023550 | | SNORD113 | | Small nucleolar RNA SNORD113/SNORD114 family [Source:RFAM;Acc:RF00181] | | | | | | | | | | | | | | | |  | |  |
| #INFO: | QUERY: | | ENSOARG00000023551 | | SNORD113 | | Small nucleolar RNA SNORD113/SNORD114 family [Source:RFAM;Acc:RF00181] | | | | | | | | | | | | | | | |  | |  |
| #INFO: | QUERY: | | ENSOARG00000023560 | | U1 | | U1 spliceosomal RNA [Source:RFAM;Acc:RF00003] | | | | | | | | | |  | |  | |  | |  | |  |
| #INFO: | QUERY: | | ENSOARG00000023575 | |  | | Small nucleolar RNA SNORD113/SNORD114 family [Source:RFAM;Acc:RF00181] | | | | | | | | | | | | | | | |  | |  |
| #INFO: | QUERY: | | ENSOARG00000023577 | | TCL6_2 | | T-cell leukemia/lymphoma 6 conserved region 2 [Source:RFAM;Acc:RF02192] | | | | | | | | | | | | | | | |  | |  |
| #INFO: | QUERY: | | ENSOARG00000023623 | | SNORD113 | | Small nucleolar RNA SNORD113/SNORD114 family [Source:RFAM;Acc:RF00181] | | | | | | | | | | | | | | | |  | |  |
| #INFO: | QUERY: | | ENSOARG00000023678 | | OAR-MIR-411A | | oar-mir-411a [Source:miRBase;Acc:MI0016918] | | | | | | | | | |  | |  | |  | |  | |  |
| #INFO: | QUERY: | | ENSOARG00000023742 | | SNORA79 | | Small nucleolar RNA SNORA79 [Source:RFAM;Acc:RF00600] | | | | | | | | | | | |  | |  | |  | |  |
| #INFO: | QUERY: | | ENSOARG00000023802 | | N/A | | N/A | |  | |  | |  | |  | |  | |  | |  | |  | |  |
| #INFO: | QUERY: | | ENSOARG00000023836 | | 5S_RRNA | | 5S ribosomal RNA [Source:RFAM;Acc:RF00001] | | | | | | | | | |  | |  | |  | |  | |  |
| #INFO: | QUERY: | | ENSOARG00000023869 | | SNORD113 | | Small nucleolar RNA SNORD113/SNORD114 family [Source:RFAM;Acc:RF00181] | | | | | | | | | | | | | | | |  | |  |
| #INFO: | QUERY: | | ENSOARG00000023967 | | SNORA70 | | Small nucleolar RNA SNORA70 [Source:RFAM;Acc:RF00156] | | | | | | | | | | | |  | |  | |  | |  |
| #INFO: | QUERY: | | ENSOARG00000023992 | | U6 | | U6 spliceosomal RNA [Source:RFAM;Acc:RF00026] | | | | | | | | | |  | |  | |  | |  | |  |
| #INFO: | QUERY: | | ENSOARG00000024000 | | OAR-MIR-758 | | oar-mir-758 [Source:miRBase;Acc:MI0016924] | | | | | | | | | |  | |  | |  | |  | |  |
| #INFO: | QUERY: | | ENSOARG00000024019 | | 5S_RRNA | | 5S ribosomal RNA [Source:RFAM;Acc:RF00001] | | | | | | | | | |  | |  | |  | |  | |  |
| #INFO: | QUERY: | | ENSOARG00000024058 | | SNORD113 | | Small nucleolar RNA SNORD113/SNORD114 family [Source:RFAM;Acc:RF00181] | | | | | | | | | | | | | | | |  | |  |
| #INFO: | QUERY: | | ENSOARG00000024070 | | U6 | | U6 spliceosomal RNA [Source:RFAM;Acc:RF00026] | | | | | | | | | |  | |  | |  | |  | |  |
| #INFO: | QUERY: | | ENSOARG00000024081 | | N/A | | N/A | |  | |  | |  | |  | |  | |  | |  | |  | |  |
| #INFO: | QUERY: | | ENSOARG00000024121 | | SNORD113 | | Small nucleolar RNA SNORD113/SNORD114 family [Source:RFAM;Acc:RF00181] | | | | | | | | | | | | | | | |  | |  |
| #INFO: | QUERY: | | ENSOARG00000024140 | | SNORD113 | | Small nucleolar RNA SNORD113/SNORD114 family [Source:RFAM;Acc:RF00181] | | | | | | | | | | | | | | | |  | |  |
| #INFO: | QUERY: | | ENSOARG00000024222 | | U1 | | U1 spliceosomal RNA [Source:RFAM;Acc:RF00003] | | | | | | | | | |  | |  | |  | |  | |  |
| #INFO: | QUERY: | | ENSOARG00000024236 | | U1 | | U1 spliceosomal RNA [Source:RFAM;Acc:RF00003] | | | | | | | | | |  | |  | |  | |  | |  |
| #INFO: | QUERY: | | ENSOARG00000024267 | | SNORD113 | | Small nucleolar RNA SNORD113/SNORD114 family [Source:RFAM;Acc:RF00181] | | | | | | | | | | | | | | | |  | |  |
| #INFO: | QUERY: | | ENSOARG00000024285 | | SNORD113 | | Small nucleolar RNA SNORD113/SNORD114 family [Source:RFAM;Acc:RF00181] | | | | | | | | | | | | | | | |  | |  |
| #INFO: | QUERY: | | ENSOARG00000024297 | | OAR-MIR-495 | | oar-mir-495 [Source:miRBase;Acc:MI0016930] | | | | | | | | | |  | |  | |  | |  | |  |
| #INFO: | QUERY: | | ENSOARG00000024298 | | SNORD113 | | Small nucleolar RNA SNORD113/SNORD114 family [Source:RFAM;Acc:RF00181] | | | | | | | | | | | | | | | |  | |  |
| #INFO: | QUERY: | | ENSOARG00000024345 | | SNORA72 | | Small nucleolar RNA SNORA72 [Source:RFAM;Acc:RF00139] | | | | | | | | | | | |  | |  | |  | |  |
| #INFO: | QUERY: | | ENSOARG00000024385 | | SNORD96 | | Small nucleolar RNA SNORD96 family [Source:RFAM;Acc:RF00055] | | | | | | | | | | | | | |  | |  | |  |
| #INFO: | QUERY: | | ENSOARG00000024394 | | SNORD113 | | Small nucleolar RNA SNORD113/SNORD114 family [Source:RFAM;Acc:RF00181] | | | | | | | | | | | | | | | |  | |  |
| #INFO: | QUERY: | | ENSOARG00000024405 | | SNORD113 | | Small nucleolar RNA SNORD113/SNORD114 family [Source:RFAM;Acc:RF00181] | | | | | | | | | | | | | | | |  | |  |
| #INFO: | QUERY: | | ENSOARG00000024450 | | U6 | | U6 spliceosomal RNA [Source:RFAM;Acc:RF00026] | | | | | | | | | |  | |  | |  | |  | |  |
| #INFO: | QUERY: | | ENSOARG00000024477 | | N/A | | N/A | |  | |  | |  | |  | |  | |  | |  | |  | |  |
| #INFO: | QUERY: | | ENSOARG00000024500 | | 5S_RRNA | | 5S ribosomal RNA [Source:RFAM;Acc:RF00001] | | | | | | | | | |  | |  | |  | |  | |  |
| #INFO: | QUERY: | | ENSOARG00000024508 | | SNORD113 | | Small nucleolar RNA SNORD113/SNORD114 family [Source:RFAM;Acc:RF00181] | | | | | | | | | | | | | | | |  | |  |
| #INFO: | QUERY: | | ENSOARG00000024597 | | SNORD113 | | Small nucleolar RNA SNORD113/SNORD114 family [Source:RFAM;Acc:RF00181] | | | | | | | | | | | | | | | |  | |  |
| #INFO: | QUERY: | | ENSOARG00000024733 | | U6 | | U6 spliceosomal RNA [Source:RFAM;Acc:RF00026] | | | | | | | | | |  | |  | |  | |  | |  |
| #INFO: | QUERY: | | ENSOARG00000024761 | | SNORD113 | | Small nucleolar RNA SNORD113/SNORD114 family [Source:RFAM;Acc:RF00181] | | | | | | | | | | | | | | | |  | |  |
| #INFO: | QUERY: | | ENSOARG00000024781 | | U6 | | U6 spliceosomal RNA [Source:RFAM;Acc:RF00026] | | | | | | | | | |  | |  | |  | |  | |  |
| #INFO: | QUERY: | | ENSOARG00000024853 | | U3 | | Small nucleolar RNA U3 [Source:RFAM;Acc:RF00012] | | | | | | | | | | | |  | |  | |  | |  |
| #INFO: | QUERY: | | ENSOARG00000024862 | | U6 | | U6 spliceosomal RNA [Source:RFAM;Acc:RF00026] | | | | | | | | | |  | |  | |  | |  | |  |
| #INFO: | QUERY: | | ENSOARG00000024875 | | SNORD113 | | Small nucleolar RNA SNORD113/SNORD114 family [Source:RFAM;Acc:RF00181] | | | | | | | | | | | | | | | |  | |  |
| #INFO: | QUERY: | | ENSOARG00000024934 | | SNORD113 | | Small nucleolar RNA SNORD113/SNORD114 family [Source:RFAM;Acc:RF00181] | | | | | | | | | | | | | | | |  | |  |
| #INFO: | QUERY: | | ENSOARG00000024985 | | U6 | | U6 spliceosomal RNA [Source:RFAM;Acc:RF00026] | | | | | | | | | |  | |  | |  | |  | |  |
| #INFO: | QUERY: | | ENSOARG00000025043 | | TCL6_1 | | T-cell leukemia/lymphoma 6 conserved region 1 [Source:RFAM;Acc:RF02191] | | | | | | | | | | | | | | | |  | |  |
| #INFO: | QUERY: | | ENSOARG00000025088 | | U6 | | U6 spliceosomal RNA [Source:RFAM;Acc:RF00026] | | | | | | | | | |  | |  | |  | |  | |  |
| #INFO: | QUERY: | | ENSOARG00000025122 | | OAR-MIR-494 | | oar-mir-494 [Source:miRBase;Acc:MI0016927] | | | | | | | | | |  | |  | |  | |  | |  |
| #INFO: | QUERY: | | ENSOARG00000025128 | | 5S_RRNA | | 5S ribosomal RNA [Source:RFAM;Acc:RF00001] | | | | | | | | | |  | |  | |  | |  | |  |
| #INFO: | QUERY: | | ENSOARG00000025158 | | SNORD113 | | Small nucleolar RNA SNORD113/SNORD114 family [Source:RFAM;Acc:RF00181] | | | | | | | | | | | | | | | |  | |  |
| #INFO: | QUERY: | | ENSOARG00000025159 | | OAR-MIR-543 | | oar-mir-543 [Source:miRBase;Acc:MI0016929] | | | | | | | | | |  | |  | |  | |  | |  |
| #INFO: | QUERY: | | ENSOARG00000025206 | | N/A | | N/A | |  | |  | |  | |  | |  | |  | |  | |  | |  |
| #INFO: | QUERY: | | ENSOARG00000025207 | | N/A | | N/A | |  | |  | |  | |  | |  | |  | |  | |  | |  |
| #INFO: | QUERY: | | ENSOARG00000025215 | | N/A | | N/A | |  | |  | |  | |  | |  | |  | |  | |  | |  |
| #INFO: | QUERY: | | ENSOARG00000025260 | | N/A | | N/A | |  | |  | |  | |  | |  | |  | |  | |  | |  |
| #INFO: | QUERY: | | ENSOARG00000025437 | | N/A | | N/A | |  | |  | |  | |  | |  | |  | |  | |  | |  |
| #INFO: | QUERY: | | ENSOARG00000025533 | | N/A | | N/A | |  | |  | |  | |  | |  | |  | |  | |  | |  |
| #INFO: | QUERY: | | ENSOARG00000025558 | | N/A | | N/A | |  | |  | |  | |  | |  | |  | |  | |  | |  |
| #INFO: | QUERY: | | ENSOARG00000025591 | | N/A | | N/A | |  | |  | |  | |  | |  | |  | |  | |  | |  |
| #INFO: | QUERY: | | ENSOARG00000025613 | | N/A | | N/A | |  | |  | |  | |  | |  | |  | |  | |  | |  |
| #INFO: | QUERY: | | ENSOARG00000025634 | | N/A | | N/A | |  | |  | |  | |  | |  | |  | |  | |  | |  |
| #INFO: | QUERY: | | ENSOARG00000025651 | | N/A | | N/A | |  | |  | |  | |  | |  | |  | |  | |  | |  |
| #INFO: | QUERY: | | ENSOARG00000025704 | | N/A | | N/A | |  | |  | |  | |  | |  | |  | |  | |  | |  |
| #INFO: | QUERY: | | ENSOARG00000025705 | | N/A | | N/A | |  | |  | |  | |  | |  | |  | |  | |  | |  |
| #INFO: | QUERY: | | ENSOARG00000025744 | | N/A | | N/A | |  | |  | |  | |  | |  | |  | |  | |  | |  |
| #INFO: | QUERY: | | ENSOARG00000025835 | | N/A | | N/A | |  | |  | |  | |  | |  | |  | |  | |  | |  |
| #INFO: | QUERY: | | ENSOARG00000025893 | | N/A | | N/A | |  | |  | |  | |  | |  | |  | |  | |  | |  |
| #INFO: | QUERY: | | ENSOARG00000026011 | | N/A | | N/A | |  | |  | |  | |  | |  | |  | |  | |  | |  |
| #INFO: | QUERY: | | ENSOARG00000026022 | | N/A | | N/A | |  | |  | |  | |  | |  | |  | |  | |  | |  |
| #INFO: | QUERY: | | ENSOARG00000026213 | | N/A | | N/A | |  | |  | |  | |  | |  | |  | |  | |  | |  |
| #INFO: | QUERY: | | ENSOARG00000026270 | | N/A | | N/A | |  | |  | |  | |  | |  | |  | |  | |  | |  |
| #INFO: | QUERY: | | ENSOARG00000026338 | | N/A | | N/A | |  | |  | |  | |  | |  | |  | |  | |  | |  |
| #INFO: | QUERY: | | ENSOARG00000026429 | | N/A | | N/A | |  | |  | |  | |  | |  | |  | |  | |  | |  |
| #INFO: | QUERY: | | ENSOARG00000026460 | | N/A | | N/A | |  | |  | |  | |  | |  | |  | |  | |  | |  |
| #INFO: | QUERY: | | ENSOARG00000026461 | | N/A | | N/A | |  | |  | |  | |  | |  | |  | |  | |  | |  |
| #INFO: | QUERY: | | ENSOARG00000026463 | | N/A | | N/A | |  | |  | |  | |  | |  | |  | |  | |  | |  |
| #INFO: | QUERY: | | ENSOARG00000026464 | | N/A | | N/A | |  | |  | |  | |  | |  | |  | |  | |  | |  |
| #INFO: | QUERY: | | ENSOARG00000026508 | | N/A | | N/A | |  | |  | |  | |  | |  | |  | |  | |  | |  |
| #INFO: | QUERY: | | ENSOARG00000026522 | | N/A | | N/A | |  | |  | |  | |  | |  | |  | |  | |  | |  |
| #INFO: | QUERY: | | ENSOARG00000026554 | | N/A | | N/A | |  | |  | |  | |  | |  | |  | |  | |  | |  |
| #INFO: | QUERY: | | ENSOARG00000026555 | | N/A | | N/A | |  | |  | |  | |  | |  | |  | |  | |  | |  |
| #INFO: | QUERY: | | ENSOARG00000026559 | | N/A | | N/A | |  | |  | |  | |  | |  | |  | |  | |  | |  |
| #INFO: | QUERY: | | ENSOARG00000026605 | | N/A | | N/A | |  | |  | |  | |  | |  | |  | |  | |  | |  |
| #INFO: | QUERY: | | ENSOARG00000026724 | | N/A | | N/A | |  | |  | |  | |  | |  | |  | |  | |  | |  |
| #INFO: | QUERY: | | ENSOARG00000026737 | | N/A | | N/A | |  | |  | |  | |  | |  | |  | |  | |  | |  |
| #INFO: | QUERY: | | ENSOARG00000026740 | | N/A | | N/A | |  | |  | |  | |  | |  | |  | |  | |  | |  |
| #INFO: | QUERY: | | ENSOARG00000026812 | | N/A | | N/A | |  | |  | |  | |  | |  | |  | |  | |  | |  |
| #INFO: | QUERY: | | ENSOARG00000026828 | | N/A | | N/A | |  | |  | |  | |  | |  | |  | |  | |  | |  |
| #INFO: | QUERY: | | ENSOARG00000026829 | | N/A | | N/A | |  | |  | |  | |  | |  | |  | |  | |  | |  |
| #INFO: | QUERY: | | ENSOARG00000026854 | | N/A | | N/A | |  | |  | |  | |  | |  | |  | |  | |  | |  |
| #INFO: | QUERY: | | ENSOARG00000026866 | | N/A | | N/A | |  | |  | |  | |  | |  | |  | |  | |  | |  |
| #INFO: | QUERY: | | ENSOARG00000026960 | | N/A | | N/A | |  | |  | |  | |  | |  | |  | |  | |  | |  |
