# Supplementary material for: Detection of candidate genes affecting milk production traits in sheep using whole‐genome sequencing analysis
Source: Vet Med Sci. 2022 Jan 11;8(3):1197–204. doi: 10.1002/vms3.731 (PMC9122411; doi:10.1002/vms3.731)
Supplement: Supplementary file 4 — TABLE S3 Gene functional enrichment categories found in positively selected genes detected by the methods Pi and FST methods [file VMS3-8-1197-s004.docx]

**Table S3.** Gene functional enrichment categories found in positively selected genes detected by the methods Pi and F_ST_ methods.

| Methods | P-value | Term | Type | Description |
| --- | --- | --- | --- | --- |
| Pi  (top 1%) | 0.0415 | GO:0071774 | ^a^BP | response to fibroblast growth factor |
|  | 0.0415 | GO:0044344 | BP | cellular response to fibroblast growth factor stimulus |
|  | 0.0442 | GO:0032025 | BP | response to cobalt ion |
|  | 0.0442 | GO:0071279 | BP | cellular response to cobalt ion |
|  | 0.0442 | GO:0033563 | BP | dorsal/ventral axon guidance |
|  | 0.023 | GO:0034637 | BP | cellular carbohydrate biosynthetic process |
|  | 0.0147 | GO:0000271 | BP | polysaccharide biosynthetic process |
|  | 0.0102 | GO:0033692 | BP | cellular polysaccharide biosynthetic process |
|  | 0.026 | GO:0007186 | BP | G-protein coupled receptor signaling pathway |
|  | 0.0102 | GO:0003008 | BP | system process |
|  | 0.00261 | GO:0050877 | BP | nervous system process |
|  | 0.0017 | GO:0007600 | BP | sensory perception |
|  | 0.00474 | GO:0007606 | BP | sensory perception of chemical stimulus |
|  | 0.00232 | GO:0007608 | BP | sensory perception of smell |
|  | 0.00319 | GO:0051606 | BP | detection of stimulus |
|  | 0.00304 | GO:0009593 | BP | detection of chemical stimulus |
|  | 0.0017 | GO:0050906 | BP | detection of stimulus involved in sensory perception |
|  | 0.00232 | GO:0050907 | BP | detection of chemical stimulus involved in sensory perception |
|  | 0.0017 | GO:0050911 | BP | detection of chemical stimulus involved in sensory perception of smell |
|  | 0.0191 | GO:0015669 | BP | gas transport |
|  | 0.0102 | GO:0015671 | BP | oxygen transport |
|  | 0.00319 | GO:0005833 | CC | hemoglobin complex |
|  | 0.0114 | GO:0019825 | MF | oxygen binding |
|  | 0.023 | GO:0140104 | MF | molecular carrier activity |
|  | 0.0102 | GO:0005344 | MF | oxygen carrier activity |
|  | 0.0102 | GO:0060089 | MF | molecular transducer activity |
|  | 0.0128 | GO:0004872 | MF | receptor activity |
|  | 0.0362 | GO:0099600 | MF | transmembrane receptor activity |
|  | 0.0372 | GO:0038023 | MF | signaling receptor activity |
|  | 0.023 | GO:0004888 | MF | transmembrane signaling receptor activity |
|  | 0.0017 | GO:0004984 | MF | olfactory receptor activity |
|  | 0.0104 | GO:0004930 | MF | G-protein coupled receptor activity |
|  | 0.00497 | HP:0200023 | Hp | Priapism |
|  | 0.00497 | HP:0008346 | Hp | Increased red cell sickling tendency |
|  | 0.0219 | HP:0012415 | Hp | Abnormal blood gas level |
|  | 0.0219 | HP:0012418 | Hp | Hypoxemia |
|  | 0.014 | HP:0005511 | Hp | Heinz body anemia |
|  | 0.014 | HP:0011981 | Hp | Pigment gallstones |
|  | 0.00497 | HP:0025409 | Hp | Abnormal spleen physiology |
|  | 0.00497 | HP:0001971 | Hp | Hypersplenism |
|  | 0.0219 | HP:0005560 | Hp | Imbalanced hemoglobin synthesis |
|  | 0.00497 | HP:0011906 | Hp | Reduced beta/alpha synthesis ratio |
|  | 0.0219 | HP:0011907 | Hp | Reduced alpha/beta synthesis ratio |
|  | 0.0357 | HP:0012100 | Hp | Abnormal circulating creatinine level |
|  | 0.0281 | HP:0003259 | Hp | Elevated serum creatinine |
| FST  (top 1%) | 0.00947 | GO:0032844 | BP | regulation of homeostatic process |

BP: Biological Process, CC: Cellular Components, MF: Molecular Functions, HP: Human Phenotype ontology.
