# Supplementary material for: Detection of candidate genes affecting milk production traits in sheep using whole‐genome sequencing analysis
Source: Vet Med Sci. 2022 Jan 11;8(3):1197–204. doi: 10.1002/vms3.731 (PMC9122411; doi:10.1002/vms3.731)
Supplement: Supplementary file 5 — TABLE S4 Candidate gene putatively selected by two statistical methods affecting milk production [file VMS3-8-1197-s002.docx]

**Table S4.** Candidate gene putatively selected by two statistical methods affecting milk production

| Methods | Genes | Chr. | Trait and references |
| --- | --- | --- | --- |
| Pi  (top 1%) | SLC35A3 | 1 | milk fat and protein percentage (Liu et al 2018) |
|  | CLDN16 | 1 | Milk yield (Suchocki et al 2016 ) |
|  | OSBPL8 | 3 | fatty acid traits  (Li et al 2014) |
|  | CSN2 | 6 | Milk protein (Taye et al 2017, Sahana et al 2014) |
|  | CSN1S1 | 6 | milk protein (Caravaca et al 2008, Kishore et al 2013,) |
|  | KCNIP4 | 6 | Milk traits (Taye et al,2017) |
|  | ST3GAL1 | 9 | oligosaccharides metabolism (Crisà et al 2016)s |
|  | ERCC8 | 16 | Fat percen (Tesfayonas 2014) |
| FST  (top 1%) | CTSK | 1 | Protein yield  (Suchocki et al 2015) |
|  | DNPEP | 2 | somatic cell count (Chen et al 2015) |
|  | PPP2R2A | 2 | Milk yield (Suchocki et al 2016 ) |
|  | CYTH4 | 3 | fat production  (De Camargo 2015) |
|  | ST8SIA1 | 3 | FAT trait (Nayeri et al 2016) |
|  | DPY19L1 | 4 | peak yield (Yodklaew et al 2017) |
|  | CUL1 | 4 | Protein yield  (Suchocki et al 2016 ) |
|  | PSPN | 5 | somatic cell count (Chen et al 2015) |
|  | ANAPC4 | 6 | Protein yield  (Suchocki et al 2016 ) |
|  | NT5DC1 | 8 | lactation persistency (Yodklaew et al, 2017 |
|  | VPS13B | 9 | total milk yield, fat yield, and protein yield milk fat and protein percentage (Liu et al 2018) |
|  | ATP6V1C1 | 9 | Protein yield  (Suchocki et al 2016 ) |
|  | METRNL | 11 | somatic cell count (Chen et al 2015) |
|  | MAD2L2 | 12 | Milk yield (Suchocki et al 2016 ) |
|  | TRMT6 | 13 | Milk yield (Zhao et al 2015) |
|  | XRN2 | 13 | Fat yield (Suchocki et al 2016 ) |
|  | PABPN1L | 14 | somatic cell count (Chen et al 2015) |
|  | TRAPPC2L | 14 | somatic cell count (Chen et al 2015) |
|  | GALNS | 16 | Protein yield  (Suchocki et al 2016 ) |
|  | ITGA2 | 16 | Milk yield (Suchocki et al 2016 ) |
|  | CCDC152 | 16 | lactation persistency and somatic cell count  (Zhao et al 2015, Chen et al 2015, Do et al 2017) |
|  | P4HTM | 19 | test day protein  (Ibeagha-Awemu et al 2016) |
|  | PSAP | 25 | Protein yield  (Suchocki et al 2016 ) |
| Both (FST  and Pi) | 5S_RRNA | 7 | Milk traits (Taye et al,2017) |
|  | U6 | 11 | Milk traits (Taye et al,2017) |
|  | U1 | 11 | Milk traits (Taye et al,2017) |
